# Systematic Analysis of the Physiological Importance of Deubiquitinating Enzymes

**DOI:** 10.1371/journal.pone.0043112

**Published:** 2012-08-24

**Authors:** Wei-Ling Tsou, Michael J. Sheedlo, Marie E. Morrow, Jessica R. Blount, Kelly M. McGregor, Chittaranjan Das, Sokol V. Todi

**Affiliations:** 1 Department of Pharmacology, Wayne State University School of Medicine, Detroit, Michigan, United States of America; 2 Department of Neurology, Wayne State University School of Medicine, Detroit, Michigan, United States of America; 3 Department of Chemistry, Purdue University, West Lafayette, Indiana, United States of America; Columbia University, United States of America

## Abstract

Deubiquitinating enzymes (DUBs) are proteases that control the post-translational modification of proteins by ubiquitin and in turn regulate diverse cellular pathways. Despite a growing understanding of DUB biology at the structural and molecular level, little is known about the physiological importance of most DUBs. Here, we systematically identify DUBs encoded by the genome of *Drosophila melanogaster* and examine their physiological importance *in vivo*. Through domain analyses we uncovered 41 *Drosophila* DUBs, most of which have human orthologues. Systematic knockdown of the vast majority of DUBs throughout the fly or in specific cell types had dramatic consequences for *Drosophila* development, adult motility or longevity. Specific DUB subclasses proved to be particularly necessary during development, while others were important in adults. Several DUBs were indispensable in neurons or glial cells during developmental stages; knockdown of others perturbed the homeostasis of ubiquitinated proteins in adult flies, or had adverse effects on wing positioning as a result of neuronal requirements. We demonstrate the physiological significance of the DUB family of enzymes in intact animals, find that there is little functional redundancy among members of this family of proteases, and provide insight for future investigations to understand DUB biology at the molecular, cellular and organismal levels.

## Introduction

**Figure 1:**
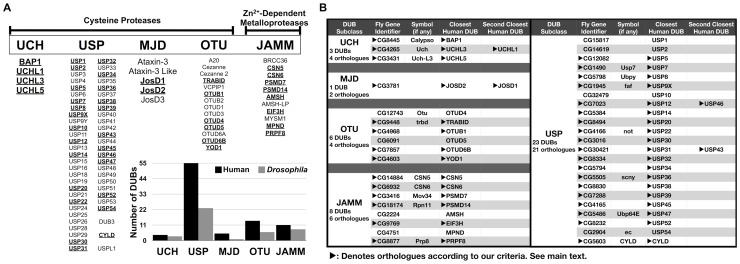
Human and *Drosophila* DUBs. A) Shown are human DUBs categorized into five subclasses based on homology at the catalytic domain [1], [3]. UCH: Ubiquitin C-terminal Hydrolases, USP: Ubiquitin-Specific Proteases, MJD: Machado-Joseph Disease Proteases, OTU: Otubain proteases, JAMM: JAB1/MPN/Mov34 Metalloenzymes. All DUB subclasses are cysteine proteases, except JAMM domain DUBs, which are Zn^2+^-dependent metalloproteases [1], [3]. Underlined and bolded: human DUBs that aligned with *Drosophila* DUBs (also see File S2). Inset: histograms show the total numbers of human DUBs (black) and fly DUBs (gray) that we identified for each subclass. B) Listed are all the *Drosophila* DUBs that we identified. Arrowheads highlight orthologues based on coverage and domain analyses (complete analysis in File S2). Not all fly DUB genes have symbols designated to them, indicating that they have not been previously characterized. Symbols were obtained from www.flybase.org.

Deubiquitinating enzymes (DUBs, also known as deubiquitinases) comprise a large family of proteases with approximately 90 members encoded by the human genome [1] (Figure 1A). DUBs regulate the post-translational modification of proteins by the small protein modifier ubiquitin. Protein ubiquitination controls many different cellular pathways and processes, from gene transcription to protein degradation, from cell division to cell death. Perturbations in ubiquitin-dependent pathways due to mutations in select DUBs are linked to malignancies and to neurological diseases (reviewed in [2]–[6]). Significant work has been conducted on the structure of several DUBs, their *in vitro* properties, molecular interactions and cell biology [2]–[6]. However, for the majority of this large family of proteases, it is unclear whether its members are physiologically necessary *in vivo*. It is also uncertain how much functional redundancy exists among a class of proteins with nearly 90 members in humans.

Protein ubiquitination occurs through the coordinated action of three types of enzymes: E1 (ubiquitin activating enzyme), E2 (ubiquitin conjugating enzyme) and E3 (ubiquitin ligase) [7]. Because ubiquitin molecules can be attached to other ubiquitins through any one of seven lysines to form chains, a target protein can be modified by a single ubiquitin or by a poly-ubiquitin chain [8]. Different types of ubiquitination have divergent effects on the fate of a protein, such as differences in localization (mono-ubiquitin) or its degradation (some types of poly-ubiquitin chains). DUBs mostly act by cleaving bonds between ubiquitin moieties or ubiquitin and another protein, thus editing ubiquitin chains, reversing protein ubiquitination, and recycling ubiquitin. However, DUBs with non-enzymatic functions have also been reported [9]–[11].

Studies in the fruit fly *Drosophila melanogaster* were among the first to investigate DUBs *in vivo*, including the DUBs Fat facets (faf) [12], [13] and Ovarian tumor (otu) [14]–[16]. While *Drosophila* is a genetically versatile organism with numerous tools that are publicly available, it has not been used extensively to elucidate DUB biology. In part, this could be due to lack of a comprehensive catalog of fly DUBs. Here we identified *Drosophila* DUBs and addressed the following: which DUBs are physiologically necessary in *Drosophila* and to what extent are they functionally redundant?

We conducted targeted RNA-interference (RNAi) screens in which individual DUBs were systematically knocked down in specific cell types or across all cells and tissues of intact flies. Most fly DUBs proved physiologically necessary for fly development, motility or longevity. There was little functional redundancy among these enzymes. To the best of our knowledge, our work comprises the first comprehensive analysis of *Drosophila* DUBs and provides clear evidence that this family of proteases is indispensible for animal physiology.

## Results and Discussion

### Identification of *Drosophila* DUBs

We used published lists of human DUBs [1], [3] to identify *Drosophila* counterparts through two sets of alignments. First, the amino acid sequence of each human DUB was aligned against the *Drosophila* proteome to uncover similarities. Next, through reverse alignments, the amino acid sequence of fly proteins identified by the first set of comparisons was used to examine domain composition and to uncover potential human orthologues (Figure 1A, File S1 and S2). For this study, we defined orthologues as protein pairs that are conserved at most domains, with at least 50% coverage (File S2). Exceptions to our criteria exist, as in the case of orthologues that have been reported previously (e.g. CYLD [17], USP36 [18]). According to our analyses, about 40% of human DUBs have *Drosophila* orthologues at the amino acid level (Figure 1B).

We found 41 fly DUBs, of which only 18 had been previously reported (Figure 1B). Out of the 41, 33 *Drosophila* DUBs fulfill our criteria as orthologues. Based on the number of members in each subdivision, the UCH and JAMM subclasses appear to be most highly conserved evolutionarily (Figure 1A). Nearly all fly DUBs aligned best with a single human DUB, except four that aligned with two human counterparts: CG4265, CG3781, CG7023, and CG30421 (File S2). These examples could be due to evolution-related specialization of certain biological processes that require specific human DUBs to perform divergent functions in the same cell, or the same functions in different cell types. Functional studies will determine whether the fly protein serves as an orthologue for both human DUBs.

### The Physiological Significance of *Drosophila* DUBs

We next examined the physiological importance of *Drosophila* DUBs by using the Gal4-UAS system [19] and RNAi to systematically knock down individual DUBs either in all cells and tissues or only in neurons (Figure S1 summarizes the Gal4-UAS system). RNAi-based approaches are powerful because they enable selective knockdown to explore the significance of individual genes *in vivo* and they provide the capacity to expediently test the physiological importance of genes either everywhere or only in specific tissues. Additionally, through different Gal4 drivers, UAS-RNAi enables gene dosage studies to uncover subtle effects that might be missed by null mutations or near-complete knockdown. RNAi-based screens were implemented successfully in the past to identify DUBs in specific cellular processes in mammalian cells (e.g. [20]–[23]).

**Figure 2:**
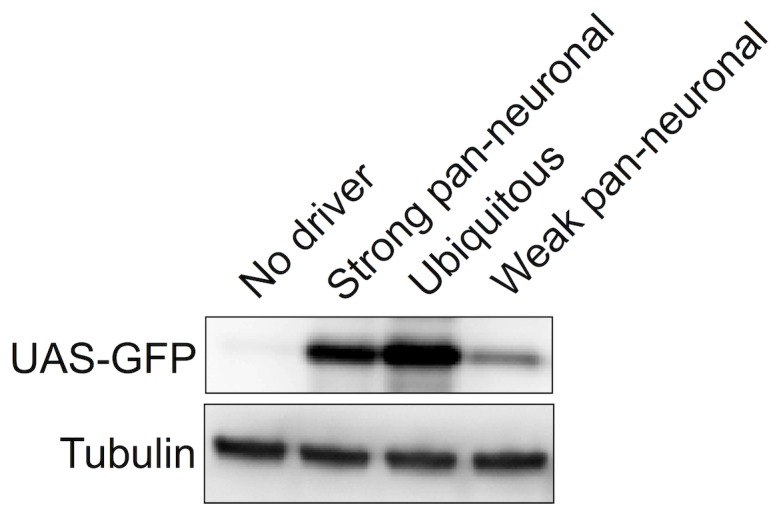
Gal4 drivers. Flies carrying UAS-GFP were crossed to flies carrying the Gal4 driver specified in the figure. Newly-eclosed adults heterozygous for UAS-GFP and the Gal4 driver were homogenized in SDS lysis buffer and loaded into SDS/PAGE gels. Western blots show how strongly each Gal4 driver expresses UAS-GFP. Blots were probed with anti-GFP and anti-tubulin antibodies. Ubiquitous driver: sqh-Gal4. Pan-neuronal drivers: elav-Gal4.

We used the following Gal4 drivers for our systematic genetic screens (Figure 2): 1) sqh-Gal4, which drives the expression of UAS-constructs everywhere throughout development [24], [25]; 2) a strong elav-Gal4 that drives the expression of UAS-constructs pan-neuronally throughout development; and 3) a weak elav-Gal4 that also drives the expression of UAS-constructs pan-neuronally. As shown in Figure 2, the strong elav-Gal4 driver expresses robustly, particularly when considering that neuronal cells do not constitute a majority of the total fly volume.

We examined the extent of knockdown for each fly DUB through quantitative RT-PCR (see Materials and Methods). The phenotypes that we monitored included fly development, adult motility (geotaxis response) and adult longevity (Figure S2). To address non-target specificity, we used RNAi lines with a specificity score between 0.99–1 (see Materials and Methods). Also, for 20 out of a total of 33 fly DUBs, we tested two or more different RNAi lines (Figure S3). Controls were offspring from crosses of Gal4 drivers to the isogenic backgrounds of RNAi lines.

**Figure 3:**
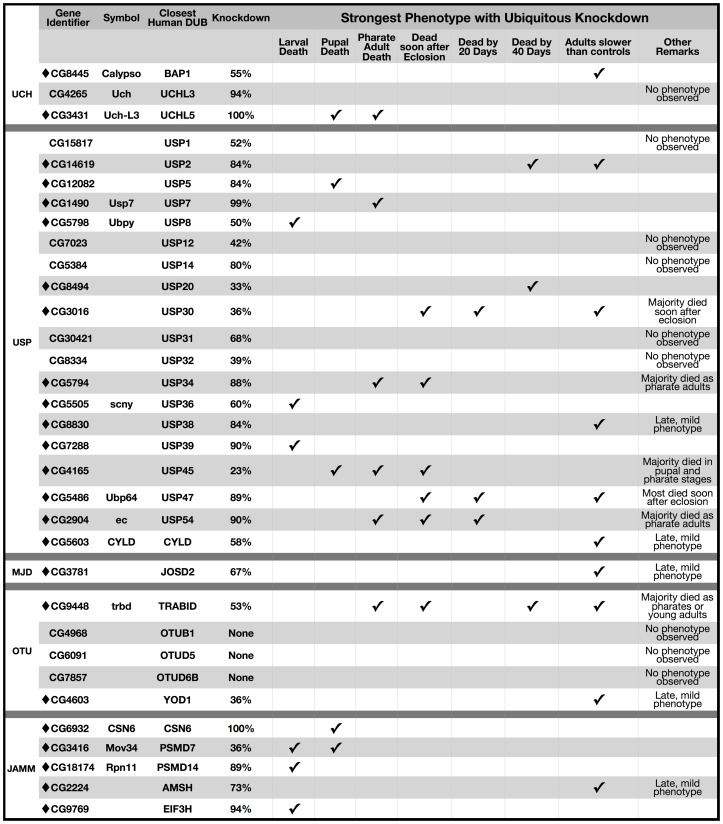
List of phenotypes when individual DUBs are knocked down throughout the fly. Listed are the strongest phenotypes associated with ubiquitous knockdown of individual fly DUBs. Diamonds highlight DUBs whose knockdown led to observable phenotypes. Experimental groups consisted of flies heterozygous for sqh-Gal4 and UAS-RNAi. Controls were heterozygous for sqh-Gal4 on the isogenic background of RNAi lines. “Dead soon after eclosion” category denotes flies that eclosed successfully from the pupal case but fell on food and died within a few hours. Late motility phenotype means it was first observed 20 or more days after eclosing from the pupal case.

As shown in Figure 3 and Figure S3, we achieved ∼80%–100% knockdown for 14 out of 33 fly DUBs tested when using the ubiquitous sqh-Gal4 driver. For most of the tested DUBs, however, knockdown was less than 80%. Nonetheless, ubiquitous knockdown of most fly DUBs had dramatic consequences for fruit fly development, adult motility or longevity, establishing a broad requirement for DUB functions *in vivo* (Figure 3). Out of 33 DUBs tested, 9 did not show a discernible phenotype when they were knocked down everywhere, and for three out of these 9 we did not observe any knockdown. Therefore, ubiquitous knockdown of 80% of tested fly DUBs had physiological consequences (Figure 3). This proportion is actually higher when considering DUBs whose pan-neuronal knockdown caused a phenotype even though ubiquitous knockdown did not, as discussed further below.

Even relatively low knockdown had physiological consequences for some DUBs. This includes the fly orthologues of USP20, USP30 and USP45, where moderate knockdown throughout the fly caused developmental lethality or earlier adult death (Figure 3). Reducing the mRNA of several DUBs by half, nearing hemizygosity, had physiological effects on the fly orthologues of BAP1, USP8, USP36 and TRABID. In other instances (e.g. CG4265) near-complete knockdown led to no discernible phenotype. Even with the latter exception, the DUB family of proteases is essential for *Drosophila* physiology.

#### Functional Redundancy

Individual knockdown of the vast majority of DUBs throughout the fly had dramatic outcomes for *Drosophila* physiology (Figure 3, Figure S3), indicating that there is little functional redundancy among fly DUBs. Based on our findings, redundancy is likely limited among human DUBs as well. This is an important notion because nearly 90 DUBs are encoded by the human genome [1] and one could assume functional overlap. Instead, according to our data, most DUBs might have evolved to fulfill distinct physiological functions by being expressed only in certain cells or tissues, by being produced during specific physiological states, by interacting with specific pools of partners, or by acting on specific substrates. This is not to say that there is no functional overlap at any level. We propose that many, or indeed most, DUBs perform at least some functions that are uniquely theirs.

Our *in vivo* analyses provide strong physiological support for studies conducted *in vitro* and in mammalian cell culture, where it was found that human DUBs have divergent structures [3], distinct substrates [2], [4], [26] and largely separate pools of interacting partners [27], indicating little functional redundancy.

#### Developmental roles for DUBs

**Figure 4:**
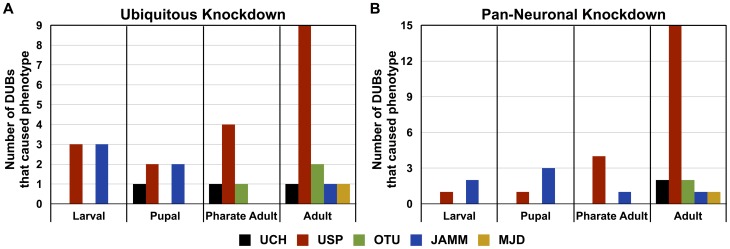
Phenotypic distribution by developmental stage. Histograms show the number of DUBs whose knockdown led to phenotype at each stage. A) sqh-Gal4 (ubiquitous) driver; details in Figure 3. B) strong elav-Gal4 (pan-neuronal) driver; details in Figure 5. In cases where two different RNAi lines targeting the same DUB led to phenotype in different stages, only the earlier stage was counted. Instances where knockdown of a specific DUB by one RNAi line led to defects in two stages were counted for each stage (e.g. knockdown of CG3416 led to death in both larval and pupal stages).

Many *Drosophila* DUBs are clearly required during development. Ubiquitous knockdown of over 40% of the tested DUBs caused lethality in larval, pupal or pharate adult stages; others were required for both adult and developmental stages or only in adults (Figure 4A, Figure S3). Based on proportionality, ubiquitous knockdown of JAMM DUBs had a greater impact during development than other subclasses (Figures 3 and 4A), highlighting the particular importance of this subclass of DUBs for developmental physiology. Perhaps JAMM DUBs are functionally indispensable for most cell types. For example, the orthologue of PSMD14, which is involved with ubiquitin recycling and is an integral component of the 19S proteasome [3], could be required more generally to assist with ubiquitin homeostasis. This orthologue can be compared to that of USP20, which may function only in specific tissues, cells, or stages. Unlike the JAMM subclass, USPs appear equally important during developmental stages and in adults (Figure 4A).

#### Cell Type Specificity

The importance of DUBs in neurons is becoming increasingly recognized, partly because mutations in a few DUBs are linked to neurological disease or neurodegeneration in mammals [5], [28]. The neuronal significance for DUBs in general, however, is unclear and much remains to be discovered about ubiquitin-dependent pathways and deubiquitination in the nervous system. Consequently, we investigated the importance of individual DUBs for the nervous system by systematically knocking down fly DUBs in neurons.

**Figure 5:**
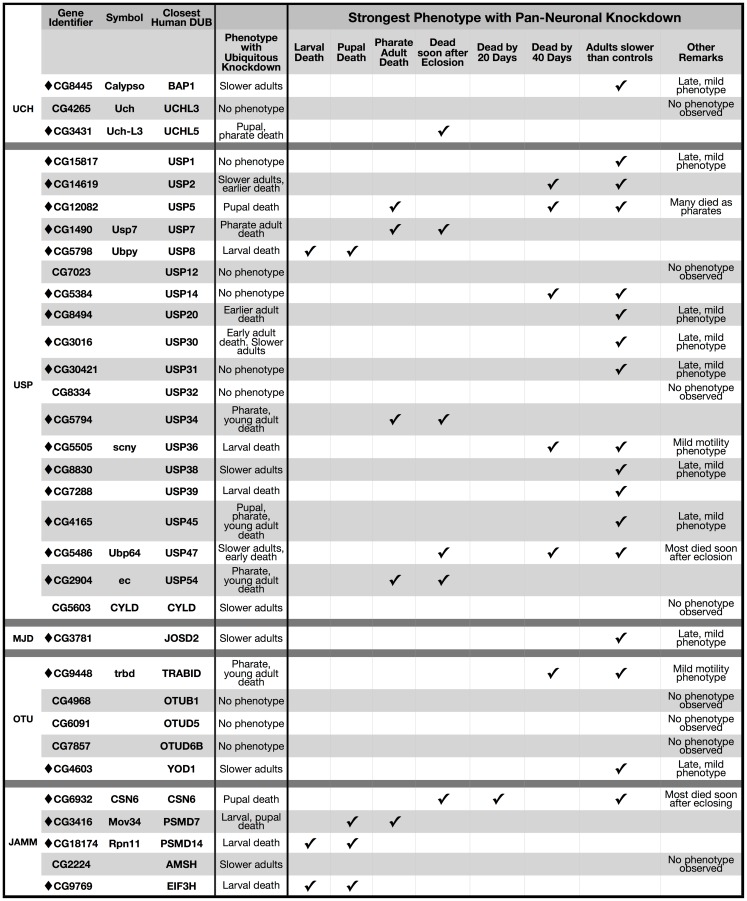
List of phenotypes when individual DUBs are knocked down pan-neuronally. Listed are the strongest phenotypes associated with pan-neuronal knockdown of individual fly DUBs. Diamonds highlight DUBs whose knockdown led to phenotypes. Experimental groups consisted of flies heterozygous for the strong elav-Gal4 driver and UAS-RNAi. Controls were heterozygous for the strong elav-Gal4 driver on the isogenic background of RNAi lines. “Dead soon after eclosion” category denotes flies that eclosed successfully from the pupal case but fell on food and died within a few hours. Late motility phenotype means it was first observed 20 or more days after eclosing from the pupal case.

Pan-neuronal knockdown of most fly DUBs caused defects during development or in adults (Figure 5). Out of the 33 DUBs tested, only 8 did not show discernible phenotypes when knocked down pan-neuronally. The distribution of phenotypic stages with pan-neuronal knockdown differed markedly from that observed with ubiquitous knockdown (Figure 4B). Pan-neuronal knockdown of ∼24% of DUBs proved lethal during developmental stages, compared to ∼42% of DUBs with ubiquitous knockdown (Figures 4B and 5).

For some DUBs, including the fly orthologues of USP7, USP8, USP34, USP47, USP54 and PSMD14, phenotypes from pan-neuronal and ubiquitous knockdowns were generally comparable, demonstrating the importance of these enzymes to overall neuronal development or function (Figure 5). For other DUBs, whose ubiquitous knockdown had dramatic developmental consequences, pan-neuronal knockdown led to considerably milder phenotypes, including the orthologues of USP39 and USP45. DUBs whose pan-neuronal knockdown caused milder phenotypes than their ubiquitous knockdown might be required by only a subset of neurons or select neuronal networks.

In a few other cases, including the USP14 and USP31/USP43 orthologues, ubiquitous knockdown did not lead to discernible problems, but pan-neuronal knockdown resulted in motility phenotype (Figure 5). This difference probably stems from stronger knockdown achieved in neurons by the pan-neuronal elav-Gal4 than by the ubiquitous sqh-Gal4 driver. However, this finding indicates the importance of USP31/USP43, USP14 and other similar DUBs, to neuronal cells. USP14 appears to function in part by maintaining ubiquitin homeostasis at synapses and its mutations cause ataxia in mice [29]–[32]. The functions of USP31 and USP43 in mammals are unclear. It will be of great interest to identify which types of neuronal cells, or networks, rely most heavily on which DUBs for ubiquitin-dependent processes.

**Figure 6:**
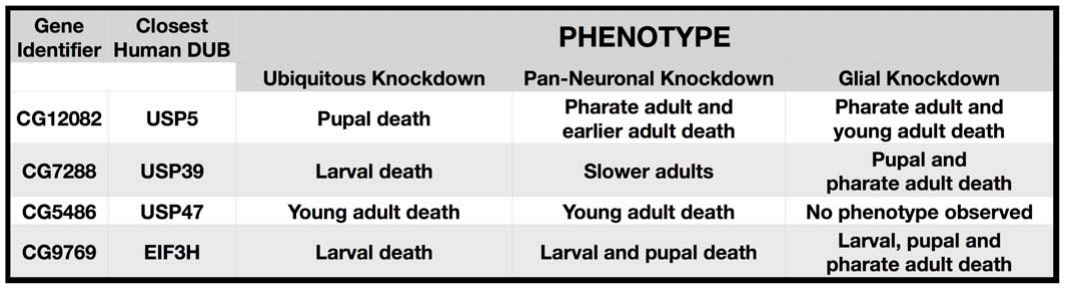
Phenotype when select DUBs are knocked down in glial cells. List compares phenotypes associated with knockdown of select fly DUBs in all cells and tissues, pan-neuronally or only in glial cells.

Since pan-neuronal knockdown of the orthologues of USP39 and a few other DUBs led to a considerably milder phenotype than their ubiquitous knockdown (Figure 5), we tested whether they are required for non-neural cells. Using repo-Gal4 [33], we knocked down the mRNA of select DUBs only in glial cells. As shown in Figure 6, knockdown of the fly orthologue of USP39 only in glia caused lethality during development, indicating that it is overall more important to glial cells than neurons. In other cases (e.g. the EIF3H orthologue), glial or neuronal knockdown led to generally comparable phenotypes. However, in the case of the USP47 orthologue, we did not observe an overt phenotype when this DUB was knocked down in glia, demonstrating the requirement of this DUB more specifically by neuronal cells (Figure 6; see also drooping wings phenotype discussed below).

**Figure 7:**
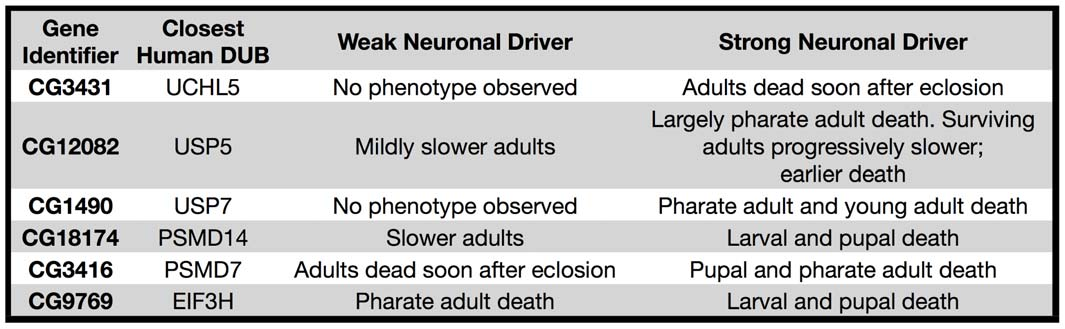
Gene dosage effects with pan-neuronal knockdown. List of phenotypes observed when individual fly DUBs were knocked down by pan-neuronal drivers with different expression strengths.

One of the advantages in using RNAi with the Gal4-UAS system is the capacity to examine gene dosage effects. For select DUBs, we used a weaker pan-neuronal driver to compare severity of phenotype to that observed with the stronger pan-neuronal driver (Figure 2). When we expressed hairpins through the weaker driver, we noticed less severe or lack of phenotype across all DUBs tested (Figure 7). The weaker driver presumably leads to a lower degree of knockdown. Thus, DUB requirement in neurons and other cell types need not be binary; different DUB levels might be required by specific cell types, populations or developmental stages.

A few points about the work presented thus far merit further discussion. Null mutations might uncover a physiological significance for DUBs whose knockdown did not lead to observable defects. Also, larger knockdown or null mutations might cause a more severe phenotype and a different distribution of phenotypes among developmental stages than what we observed. For example, null mutation of the fly orthologue of USP8 (CG5798) is embryonic lethal [34]. In our hands, ubiquitous knockdown of CG5798 did not completely eliminate its mRNA and instead led to larval or pupal death (details in Figure S3). Lower, or developmentally timed, knockdown could lead to milder phenotype. This example highlights the strength the Gal4-UAS-RNAi approach, which allows for the examination of pathways that are controlled by specific DUBs after bypassing earlier lethality and without the need to generate hypomorphic mutations.

Similar to null mutations, RNAi-based approaches cannot discriminate between catalytic and non-catalytic roles of DUBs. According to recent evidence, DUBs can play roles independent of their catalytic activity, in some cases by regulating protein complex formation [10], [11]. For some DUBs we can conclude that phenotypes stem directly from lack of protease activity; knockdown of proteasome-associated DUBs PSMD14 or UCHL5 would hamper ubiquitin recycling at the proteasome. For others, the generation of domain mutations will likely be required to define their functions.

Finally, for most cases where we used two different RNAi lines to target the same DUB, we observed either similar phenotypes or phenotype severity correlated with efficiency of knockdown (Figure S3). However, this was not as clear for the orthologues of USP36 and UCHL5. For CG5505, the USP36 orthologue, we observed a more severe phenotype with lower knockdown. For CG3431, the UCHL5 orthologue, we achieved comparable knockdown with two different RNAi lines, but phenotype severity was not identical. Perhaps there is a confounding effect from the site of insertion of UAS-RNAi. Nonetheless, because we observed clear phenotypes with all RNAi lines targeting CG5505 or CG3431, we conclude that these genes are essential for *Drosophila* development.

### Insight into Molecular Mechanisms of DUBs in *Drosophila*

To examine cellular effects from the ubiquitous knockdown of select DUBs, we analyzed the distribution and levels of ubiquitinated proteins through western blotting from whole fly lysates. Western blotting has been used previously to demonstrate that knockdown, knockout, or mutations in a single DUB can be associated with differences in the levels or distribution of ubiquitinated species in mammalian cell culture and tissue [29], [35]–[37].

**Figure 8:**
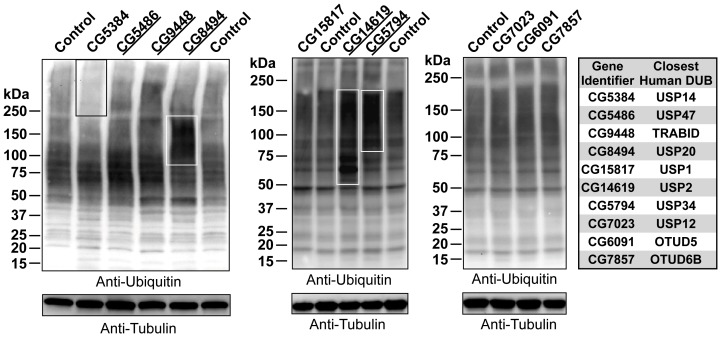
Differences in distribution and levels of ubiquitinated species in whole fly lysates. Shown are western blots of whole, newly-eclosed adult flies homogenized in SDS lysis buffer and electrophoresed in SDS-PAGE gels. Experimental groups were heterozygous for sqh-Gal4 and UAS-RNAi. Controls were heterozygous for sqh-Gal4 on the isogenic background of RNAi lines. Boxes highlight some areas with visible differences in ubiquitinated species. Western blots are representative of at least three independent repeats with similar results. Underlined: ubiquitous knockdown led to phenotype (Figure 3).

As shown in Figure 8, knockdown of several DUBs throughout the fly led to markedly different levels and distribution of ubiquitinated species compared to controls. Differences in ubiquitin smears involved multiple species (Figure 8). Generally, ubiquitous knockdown led to higher levels of ubiquitinated species, as in the case of CG8494 (USP20 orthologue), CG14619 (closest to USP2) and CG5794 (USP34 orthologue). Ubiquitous knockdown of these DUBs caused early lethality (Figure 3). USP2 and USP20 have been linked to TNF pathways in mammalian cells [38]–[40], while USP34 has been implicated in Wnt/β-catenin signaling [41]. Other cellular functions are also likely for these DUBs, but previous reports will inform future endeavors to identify substrates and cellular pathways through quantitative mass spectrometry using intact animals.

Ubiquitous knockdown of the USP14 orthologue did not result in discernible phenotype (Figure 3), although we noticed differences in intensity of ubiquitinated species in western blots (Figure 8). Sensitized tests, stronger ubiquitous knockdown or null mutations might uncover phenotypes that our assays did not. In support of this notion, pan-neuronal knockdown of the USP14 orthologue caused locomotion defects in adults (Figure 5). Western blotting does not indicate whether knockdown of a specific DUB leads to overall differences in ubiquitinated species because of multiple substrates or a single one. However, this analysis provides valuable information on global effects that DUBs can have on ubiquitin-dependent pathways.

**Figure 9:**
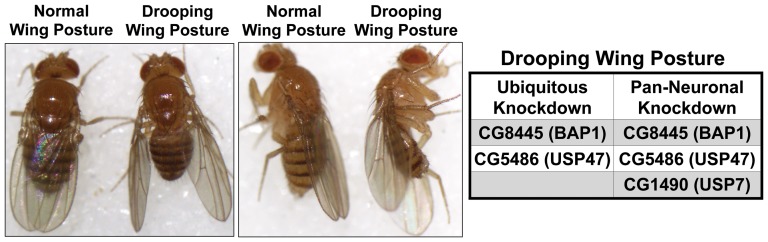
Wing postural defects. Shown are representative cases of flies with normal or drooping wings. Drooping wings was generally an age-dependent phenotype. Box lists DUBs whose knockdown throughout the fly or only in neurons led to this phenotype. Ubiquitous knockdown of CG1490 was lethal before adults eclosed from the pupal case.

Further insight into cell type specificity and function of some DUBs can also be gained from a drooping wings phenotype that we observed with the knockdown of select proteases (Figure 9). Drooping wings can result from diseased, malfunctioning or degenerating flight muscles [42], [43]. Two DUBs whose ubiquitous knockdown led to drooping wings were CG8445 (the orthologue of BAP1) and CG5486 (the orthologue of USP47). In both cases, this was a progressive phenotype that corresponded with decreased motility. Interestingly, drooping wings were also observed when select DUBs were knocked down only pan-neuronally (Figure 9). Abnormal wing posture as a result of neuronal knockdown implicates DUBs in neurons supplying flight muscles. Importantly, this phenotype identifies cell types that should be investigated first to elucidate the function of these proteases *in vivo*. Future work will determine the role of these DUBs in neurons.

## Conclusions

**Figure 10:**
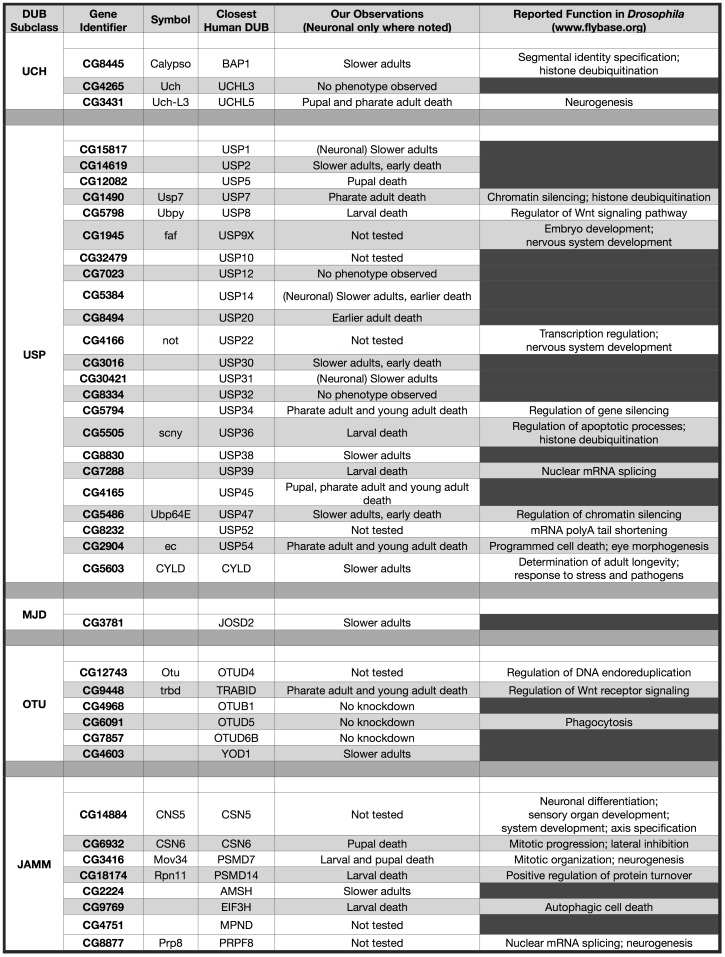
Comprehensive list of fly DUBs and their physiological significance. Listed are all the fly DUBs that we identified, highlighting previously reported functions and our current findings. Not tested: DUBs that we did not examine either because of lack of reagents, too many non-specific targets from existing RNAi lines, or because the function of these DUBs is well characterized in flies. Empty cells: as of this publication, no information had been previously reported for these DUBs.

We have presented a comprehensive list of *Drosophila* DUBs and examined their physiological significance through systematic knockdown screens in intact flies. We found 41 fly DUBs, most of which were not characterized previously (Figure 10). Thirty-three of the DUBs identified fulfill our criteria for human orthologues. The vast majority of fly DUBs proved indispensable for *Drosophila* development, adult motility, or longevity (Figures 3, 5, 10). Some DUBs were necessary for neuronal cells, while others were required by cells other than neurons (e.g. glia; Figure 6), providing critical insight into cell type specificity for these proteases. Our data demonstrate that the DUB family of enzymes is necessary *in vivo*. Our genetic screens also lead us to conclude that there is little functional redundancy among DUBs. Future studies will extend our work to provide mechanistic and molecular details into the biology of this large class of proteases that controls organismal development, function and disease.

## Materials and Methods

### Fly stocks, crosses and observations

Flies were reared and maintained in standard cornmeal/molasses/yeast medium in diurnally-controlled, 25°C environment. *Drosophila* RNAi stocks were purchased from the Vienna *Drosophila* RNAi Center (VDRC) and Bloomington *Drosophila* Stock Center (BDSC). We thank Dr. Norbert Perrimon (Harvard Medical School) for providing the *Drosophila* RNAi stock TRiP-CG5798. Gal4 stocks have been previously described [24], [25], [44]. Figure S3 lists the stock number for each *Drosophila* RNAi line and the extent of knockdown we achieved through the ubiquitous sqh-Gal4 driver, as assayed by quantitative RT-PCR (see below). To address non-target specificity, we only used VDRC lines with a specificity (s19) score of 0.99 or higher. The specificity score is determined as follows: s19 = ∑ON-target matches/(∑ON-target matches+∑OFF-target matches), and is reported at www.vdrc.at. Similar scores were not available for non-VDRC stocks, but the lines we utilized do not target other genes (as reported by BDSC; flystocks.bio.indiana.edu).

Four or more separate sets of crosses were set up for each RNAi line with each Gal4 driver, yielding similar results. At least 200 flies per genotype were examined. For experimental groups, parents from RNAi lines were crossed to parents from Gal4 drivers and heterozygous offspring containing both Gal4 driver and UAS-RNAi were collected and monitored. Controls were heterozygous offspring from isogenic non-RNAi lines crossed to Gal4 drivers, and in cases of balanced RNAi stocks also from balanced sibling controls containing Gal4 driver but lacking the UAS-RNAi construct. Qualitative examination of adult motility was performed through the geotaxis response: vials containing 20–25 adult flies were tapped down lightly and fly movement up the vial wall was compared to controls. Groups were considered slower if they lagged behind controls approximately 1/5^th^ or more of the total distance travelled in 15 seconds or when the controls reached the top, whichever occurred first. “Mild motility phenotype” denotes flies around this margin. Locomotion was examined every two or three days for a total of 40 days. Because motility decreases normally with age, we monitored same-age cohorts. Fly death was monitored daily. Longevity of control flies and those without a longevity phenotype was 60–70+ days.

### Amino acid alignments and domain analyses

The amino acid sequence of each human DUB was obtained from Ensembl, using the longest isoform when multiple were predicted. Amino acid alignments against the *Drosophila* proteome were conducted using BLASTp from NCBI. Amino acid sequences for fly DUBs were retrieved from FlyBase and are listed in File S1. Domain architectures for human and fly DUBs were compiled from UniProt, SMART, PROSITE and InterPro. Most DUB domains could be found on UniProt and PROSITE, MIT domains on InterPro and coiled coil domains on UniProt and SMART. A pairwise local BLAST was obtained using the ClustalW algorithm to validate identification of fly domains by percent identity and E-values (cutoff of 0.001).

### Quantitative RT-PCR

Total RNA was extracted from young adult flies, except cases of larval, pupal or pharate adult lethality, where RNA was extracted from stages preceding death. With the exception of larvae, total RNA was extracted using TRIzol Reagent (Invitrogen). For larvae, RNA was extracted using RNAqueous®-Micro Scale RNA Isolation Kit (Ambion). Extracted RNA was treated with TURBO DNase (Ambion) to eliminate contaminating DNA. Reverse transcription was performed with the High Capacity Kit (ABI) and DUB knockdown was quantified by using the PlusOne real-time quantitative system with fast SYBR green (ABI). Rp49 and actin were used as control primers, with similar results. Primer sequences are listed in File S3. Similar to phenotypic observations, animals heterozygous for sqh-Gal4 and UAS-RNAi were compared to animals heterozygous for sqh-Gal4 on the isogenic background of UAS-RNAi lines of the same stage.

### Western Blotting and antibodies

15–20 flies per genotype were frozen in dry ice and homogenized in boiling 2%SDS/100 mM DTT lysis buffer by mechanical disruption. 50 µL of homogenizing buffer were used per fly. Homogenates were boiled 10 minutes, sonicated for 20 seconds and centrifuged at top speed for 15 minutes. Supernatant was loaded into SDS/PAGE gels and transferred onto PVDF membrane for western blotting. Western blotting was conducted and imaged as described previously [37], [45]. Antibodies used were: mouse monoclonal anti-tubulin (Sigma-Aldrich) at 1∶15,000; rabbit anti-GFP (Santa Cruz Biotechnology) at 1∶500; rabbit anti-ubiquitin (DAKO) at 1∶500. Peroxidase-conjugated secondary antibodies (Jackson Immunoresearch) were used at 1∶10,000.

## Supporting Information

Figure S1 **Gal4-UAS approach to RNAi.** Flies encoding a Gal4 driver are crossed to flies encoding UAS-RNAi targeting a specific DUB gene. In the resulting offspring, the Gal4 driver binds to UAS sequences and drives the expression of inverted repeats in a tissue-specific manner. Hairpin RNAs that result from the expression of the inverted repeat are processed and bind to DUB mRNA, leading to their destruction. Schematic was redrawn from a similar diagram posted by VDRC on www.vdrc.at.
(TIFF)

Figure S2 **Phenotypic observations.** Diagram depicts the developmental stages of the fruit fly and what was monitored in adults. Developmental time is not drawn to scale.
(TIFF)

Figure S3 **RNAi lines used and phenotypic observations.** Complete list of the fly DUBs that we targeted with RNAi and phenotypic observations from each line. Also shown are the stock numbers for lines from VDRC and BDSC, as well as extent of knockdown achieved by the ubiquitous driver sqh-Gal4. ND: not determined. Diamonds highlight DUBs whose knockdown led to discernible phenotype. *Drosophila* stock HMS TRiP-CG5798 was a kind gift from the laboratory of Dr. Norbert Perrimon (Harvard Medical School) before the line was deposited into BDSC.
(TIFF)

File S1 **Amino acid sequences of fly DUBs.**
(PDF)

File S2 **Human and fly DUB domain designations and comparisons.**
(PDF)

File S3 **Quantitative RT-PCR primers.**
(PDF)
