# Supplementary figures and images for: Systematic Analysis of the Physiological Importance of Deubiquitinating Enzymes
Source: PLoS One. 2012 Aug 24;7(8):e43112. doi: 10.1371/journal.pone.0043112 (PMC3427330; doi:10.1371/journal.pone.0043112)

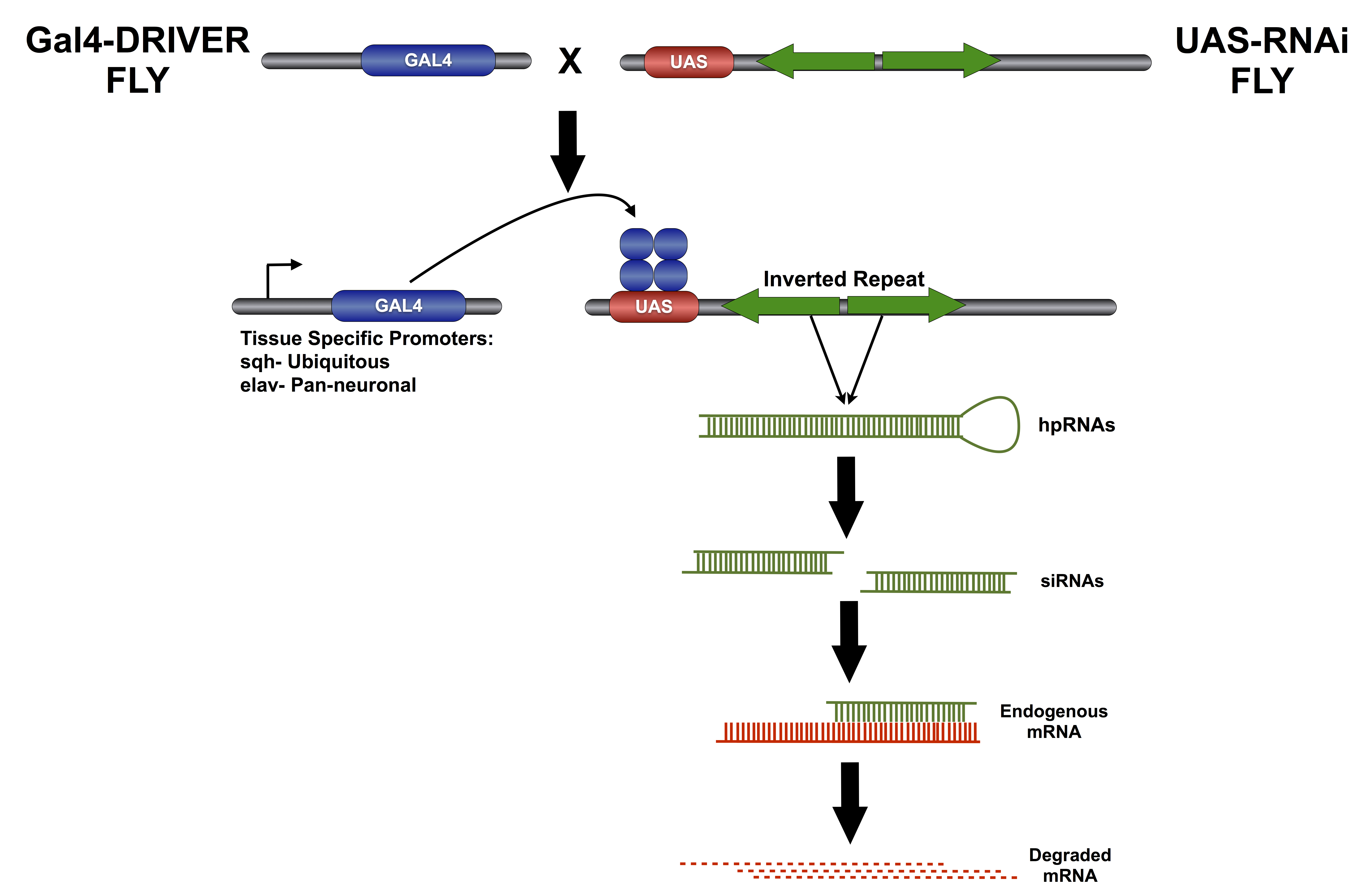

Supplement: Figure S1 — Gal4-UAS approach to RNAi. Flies encoding a Gal4 driver are crossed to flies encoding UAS-RNAi targeting a specific DUB gene. In the resulting offspring, the Gal4 driver binds to UAS sequences and drives the expression of inverted repeats in a tissue-specific manner. Hairpin RNAs that result from the expression of the inverted repeat are processed and bind to DUB mRNA, leading to their destruction. Schematic was redrawn from a similar diagram posted by VDRC on www.vdrc.at. (TIFF) [file pone.0043112.s001.tiff]

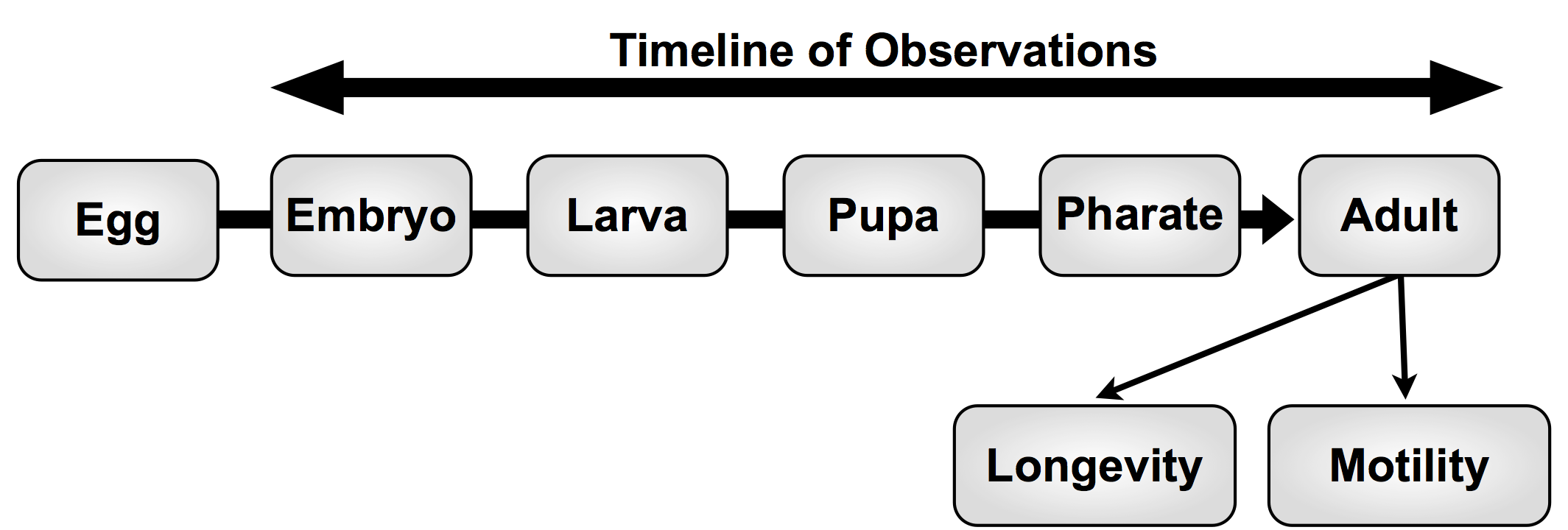

Supplement: Figure S2 — Phenotypic observations. Diagram depicts the developmental stages of the fruit fly and what was monitored in adults. Developmental time is not drawn to scale. (TIFF) [file pone.0043112.s002.tiff]

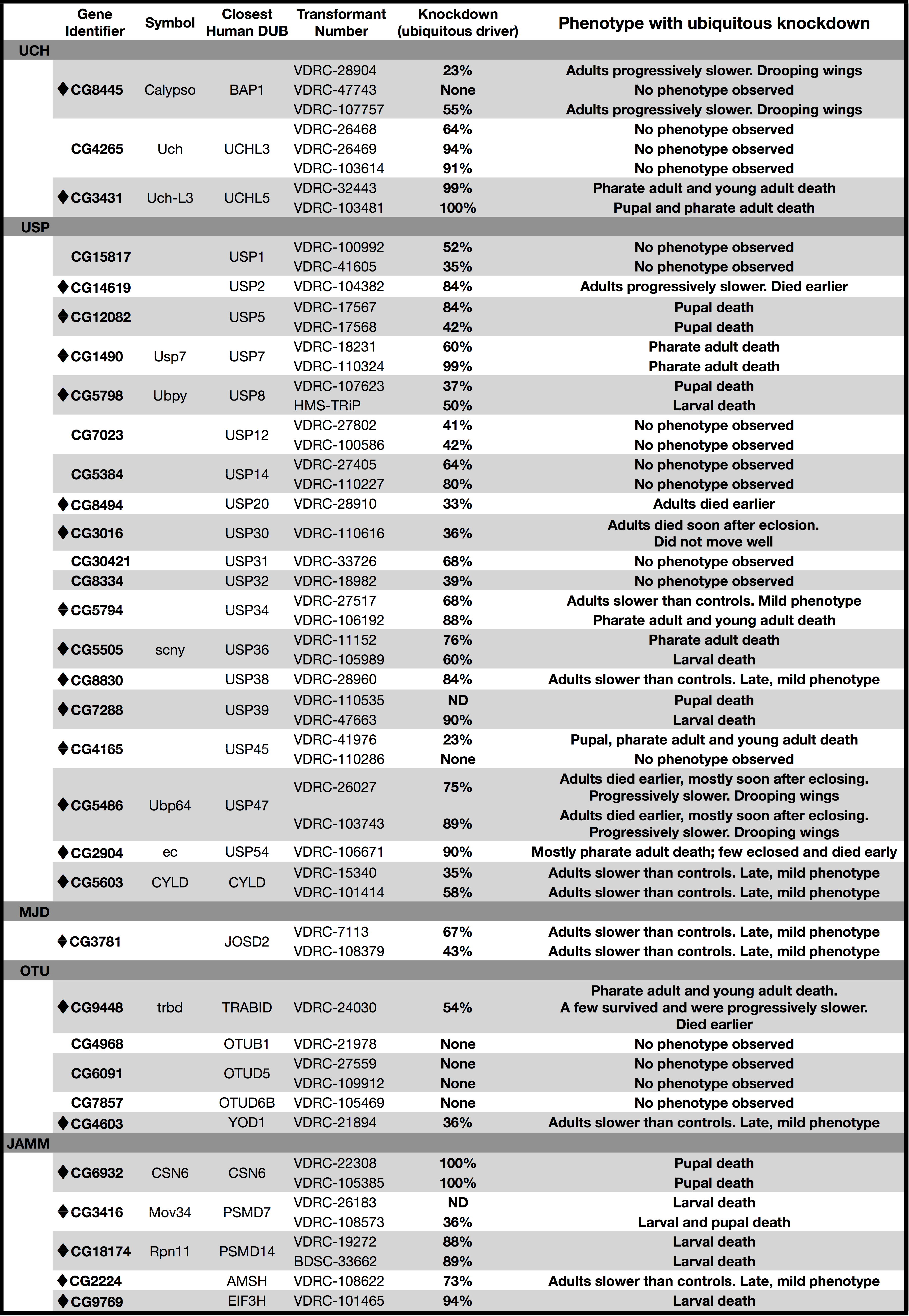

Supplement: Figure S3 — RNAi lines used and phenotypic observations. Complete list of the fly DUBs that we targeted with RNAi and phenotypic observations from each line. Also shown are the stock numbers for lines from VDRC and BDSC, as well as extent of knockdown achieved by the ubiquitous driver sqh-Gal4. ND: not determined. Diamonds highlight DUBs whose knockdown led to discernible phenotype. Drosophila stock HMS TRiP-CG5798 was a kind gift from the laboratory of Dr. Norbert Perrimon (Harvard Medical School) before the line was deposited into BDSC. (TIFF) [file pone.0043112.s003.tiff]
