# Supplementary material for: Systematic Analysis of the Physiological Importance of Deubiquitinating Enzymes
Source: PLoS One. 2012 Aug 24;7(8):e43112. doi: 10.1371/journal.pone.0043112 (PMC3427330; doi:10.1371/journal.pone.0043112)
Supplement: File S1 — Amino acid sequences of fly DUBs. (PDF) [file pone.0043112.s004.pdf]

Fly Gene Identifier	Amino Acid Sequence (www.flybase.org) - longest isoforms were generally used in cases with multiple isoforms
**CG8445**	MNAAGGGSGAQAAAVAAGNNSLSHNALLSTASGATTMPMAQLADGWLELE<br>SDPGLFTLLLKDFGCHDVQVEEVYDLQKPIESPYGFIFLFRWIEERRARR<br>KIVETTAEIFVKDEEAISSIFFAQQVVPNSCATHALLSVLLNCNENNLQL<br>GDTLSRLKTHTKGMSPENKGLAIGNTPELACAHNSHAMPQARRRLERTGA<br>GVSSCRFTGEAFHFVSFVPINGQLFELDGLKPYPMNHGGWEDSEDWTDKF<br>RRVMAERLGIATGEQDIRFNLMAVVPDRRIAITHKLKMLRTNQAIVSGTL<br>QKLLKADEQGESGNGDSQRPDTPTTLLEPSAFTARDLQSLLKNLDTEIAI<br>NEQHLADENDRRHMFKVDASRRTHNYDKFICTFLSMLAHQGVLGELVSQH<br>LLPSKKVSGQGAANRISKQSTTASAGGSTAAGTASTPKTQQQQAAAAKNG<br>KSPSKTPGRRRKGRNKCRKRK
**CG4265**	MLTWTPLESNPEVLTKYIHKLGVSPAWSVTDVIGLEDDTLEWIPRPVKAF<br>ILLFPCSETYEKHRAEEHDRIKEVEEQHPEDLFYMRQFTHNACGTVALIH<br>SVANNKEVDIDRGVLKDFLEKTASLSPEERGRALEKDEKFTADHEALAQE<br>GQTNAANHEKVIHHFIALVNKEGTLYELDGRKSFPIKHGPTSEETFVKDA<br>AKVCKEFMARDPNEVRFTVLALTAAQQ
**CG3431**	MGDGAGNWCLIESDPGVFTELIREFGCDGAQVEEIWSLDSESFKNLEPIH<br>GLIFLFKWVQEDEPAGKVVLDRENIFFAKQVINNACATQAILSLLMNLDH<br>EDIKLGETLTNFKEFCQCFDPYNKGLTLSNASQIRTVHNSFARPTLFELD<br>TKNQKKDDDVYHFVGYMPIGGRLYELDGLREGPIDLGEIKSEQNWIDVVR<br>PIIEKRMQRYSDGEIHFNLMALISDRQRIYEQQIEKLLNPAPNAMDTEED<br>RQAEISSLRTYIEYEIQKKKRYKVENVRRKHNYLPFIVELLKILGENGQL<br>MPIYEKAKQRALEREQAQRKKDTD
**CG5798**	MAKLKKLHMSSNLDDLEKMSIIPDLRSKGMKILLNTARKLYMTAEEYRLD<br>GDEELAYITYMKYFNMLTAIHKKSDYPSHKTTVRQMLGDTESNRRIMDTL<br>EEIINSLRHRYAQQHQAPEPIAPDLVSNGRAGVDSPITQPTQYARLGLIT<br>CQDLYRRMQEKSVLVMDCRPSADYEASHLTYYCAFNVPEELITPGMSAGR<br>LQARLSSSAKASWASRSVKDSVVLMDWNTKDAQPATNTAISTLLDILKNW<br>DPDVTYRAPIQIVEGGYEYFIMMYPTHCTNPSVQAPQQNNNDIETIDDIE<br>YPSIHDITMKEDISAKDFRPRPDFNRANKPAATRVNEQGISRPSPPAKPI<br>AEIMRDQAEFLQRAEQNDEQLEKASKMWKRQAAEGDGLNATEDQELHFRI<br>LQLESKAQDYIVENNRLREELSRIQELHNVTQQLSQKEVEATRNIESKIR<br>ERQRLDEQHELERQERERLLAIARETKKHYKSPTPSGPPSPGRNLEDVHV<br>VSDSLESLLQLTGDPDPTIAPNKAEIPTFDRAMKPQPRNVERTSQRVRDF<br>SPVIGQNVGRGLTGLKNLGNTCYMNSILQCLSNTPQLTEYCISDKYKNYI<br>SRSNKTNGQVIEEVAALIKELWNGQYKCVASRDLRYVVGQYQKIFRGVDQ<br>QDSHEFLTILMDWLHSDLQTLHVPRQREMISASEKAWLEFTKAKESMILH<br>LFYGQMKSTVKCVACHKESATYESFSNLSLELPPNSNVCQLNQCMDMYFS<br>GERIHGWNCPSCKTKRDAIKKLDISKLPPVLVVHLKRFYADPSNSGSYMK<br>KQNYLRFPLENLDMNPYIARAESRAVTPKTYQLYAVSNHYGTMEGGHYTA<br>FCKSANYGKWFKFDDQVVSALDSSNVVSSAAYILFYTWLPPMQVPL

**CG8334**	MLYAACGGTQRGISFNDLLCGLVLITRGTQAEKTKFLWNLYCNDAGTFII<br>KSDYVRNVNLAPFESVSLFAQSERVNFEQFQDWIIKHRNATVLSKWLLSD<br>NCVSLTSELETPTFYQSLAGVTHLEEKDIGDLEKEFWRLKNTSQNGQIDL<br>QFLGPLISPPIPKNALAGLFNAFDENRDGHIDFKELCCGVSAACRGPGVE<br>RTRFCFKIFDVDRDGVLSHDETLQMINVLLLVAKENQESQQYKDLTKQLV<br>ISDLLEFGQRRSPDGTPSKLTRDNVSLTAEDFMLWTVQCDLRLMQPLLDL<br>IFELCHIVFGLWPQCKHMENDIVRGWLRREERRPYRVGQFWYLITHDWWL<br>SWMQYTQHTTHTCDYCKRTASQRTAVDEALVCDESFNTHSLEQHDSYSLG<br>SGTGSASGSGSASSGISAGRHCGPVRPGPIDNSNLITANPFRNVRTLTGE<br>GGHLKRDTPLVQNHDFELVPKSLWKALNRWYGDNLPLPRQVIQPPNSDVE<br>LELYPLNLRILLHQAQPSQTGVGGGTQLGSWGSTVSGGYGVLASGGGYAA<br>IAVSSVLQPPKRYLAYTAAFSRLATVRQVGEFLCEQLRLKSEDIRLWHVP<br>QLDNGAILLEEDAMCLKELLIRDNDQLLLEIRNKDLTWPEELGSLATAQC<br>GQGAGTPGDRRRLTRSSIMSVHAPGATGLHNLGNTCFMNAALQVLFNTQP<br>LAQYFQREMHRFEVNAANKLGTKGQLAMRYAELLKEVWTATTRSVAPLKL<br>RFCVNKYAPQFAGGGQHDSQELLEWLLDALHEDLNRVMEKPYSELKDSNG<br>RPDKIVAAEAWSQHHARNQSIIIDLFYGQLKSKVSCLGCGHESVRFDPFS<br>LLSLPLPVENYIYFEVLVILLDGSVPIKYGFRLNSDCKYSHLKHKLSTMC<br>SLPPNLMLVCELWNSQIRQVLNDDEKLRTQSAKELYVYQLPEQSMRTRSN<br>SGLSMHIEQGLKDIQRSSALITSAQDSLSSLSTLQTSSHRASSRVLCNGH<br>VSGLDVEGEAEVGTDVSQCNSNSNYNPIVSTYSGNGSGDNQVHELLPDEA<br>GKDSELAPQRRNCCYSRMIYRRVVFDCECEIRQESASVCFSIESLILSSS<br>PENTFMHGAAAQQKRVSSAKLLHTESNTSSMSYTNHSGENSMESSLTEPI<br>PLADLEPVSSRNGSGGEDCSYRTSPNDSSGLSTGHTLGASLDVDEQAEEG<br>NAEDHDQPDQITTSQPETSSGVYSRRSSQPPHKAGKYLVAVHRKITRHDS<br>YFLSYHKTRPSLFGVPLLIPNSEGGTHKDLYCAVWLQVSRLLSPLPATTE<br>QANHAADCDDSLGYDFPFTLRAVKADGLTCAICPWSSFCRGCEIRCNNDY<br>VLQGALPPINAAASNTSTPKMNAKFPSLPNLEAKRTPEYTASLSYTPTTK<br>YFEDFTIAIDWDPTALHLRYQSTLERLWVDHETIAISRREQVEPVDLNHC<br>LRAFTSEEKLEQWYHCSHCKGKKPATKKLQIWKLPPILIVHLKRFNCVNG<br>KWVKSQKVVHFPFDDFDPTPYLASVPQETILRHKELLELKNDAEMTMATN<br>EVVSELDEIDAPSKEVKEELPNQTGSTKATASPPPTGNILRQSKTKNAVR<br>RQRLISTSLTKTPIVDGEFEDYHQHRLKPDVDQFDPRYRLYAVVSHSGML<br>NGGHYISYASNATGSWYCYNDSSCREISQKPVIDPSAAYLLFYERKGLDY<br>EPYLPNIEGRTLPNTASVPLEVDETEGELKKLCSIS

**CG12082**	MEELRKHLSKVNVPCASGAGSPPIYKDECVYSYDNPETPTGLYVCLHSFL<br>GFGEAYVREYADKTGNRVFLHIQRVKTIKEGADMEAECAESEAGPERKIT<br>RLAIGVEGGYNESDMAKKYEIKDTYSIVVAPHLDKKLPYPDPELPMRVTQ<br>SVEAILAADSAIAKLEKATLMGTWDGEVRQASKYADNLQQLDNGKRIPPS<br>GWQCEKCDLTNNLWLNLTDGSIMCGRKFFDGSGGNDHAVEHYRVTGFPLA<br>VKLGTITADGKSDVFSYPEDEMVLDPHLERHLSHFGINMAAMKKSEKSMV<br>ELELDINQRIGEWTALTESESELQPVAGPGYTGMRNLGNSCYINSVMQVL<br>FVIPDFQQRFVGTGAERYFKEFPSDPANDFNIQMAKLGTGLQSGKYSSIA<br>ENTLDTDHSTGISPAMFKNIVGKNHPDFSTKQQQDANDFYLHLLTLLDRN<br>SRNQTNPADALKFLLEDRVECLASHKVKYNTREEYSFRLPVPLDKATNLD<br>EVREFQERKKAARETGQRLPDRDIVRHKVPLQACLERFFGPELIEQFYST<br>AIGSKTNARKITRLATMPDCLMIHVGKFTLGDDWVPKKLDVSVDMPDELD<br>LSNWRSAGGLQPGEEALPEPATEEVKFAFDEAVMSELLTMGFPPEACKRA<br>CYHTKNSGLEAASNWLMEHIADEDISEPFVVPNNSIGDCAANQFVANPES<br>LAMLMSMGFDERQAVAALKATDGNVERATDWIFSHADSIGVEDAAPAANS<br>SAAAASSTPNKTNYRDGRGKYRLVAFISHMGTSAQVGHYVCHIRKKGEWV<br>IFNDSKVAKSQNPPKDLGYLYLYMREQ
**CG1490**	MEIETDQSIEAMDTQDTQEVEILTSDLQQTQQQRNSPPQLPKFKNLIQPQ<br>LHAVGAVTQLPSENGNMPPQQLLADSSSTSFGDGEAMGIDDESKEDQFRS<br>ETTFSFTVENVVQLKSQRLSPPVYVRMLPWRIMVIPNDRALGFFLQCNGE<br>NDSPTWSCNAIAELRLKCHKPDAQPFTRARIKHLFYSKENDYGYSNFITW<br>QELKDSEKSYVHNNSITLEVHVVADAPHGVLWDSKKHTGYVGLKNQGATC<br>YMNSLLQTLYFTNSLRLSVYRIPTEADDSSKSVGLSLQRVFHELQFGDRP<br>VGTKKLTKSFGWETLDSFMQHDVQEFLRVLLDKLESKMKGTILEGTIPGL<br>FEGKMSSYIKCKNVDYNSTRYETFYDIQLNIKDKKNIYESFQDYVAPETL<br>EGDNKYDAGVHGLQEASKGVIFTSFPPVLHLHLMRFQYDPVTDSSIKYND<br>RFEFYEHINLDRYLAESENTLADYVLHAVLVHSGDNHGGHYVVFINPKAD<br>GRWFKFDDDVVSSCRKQEAIEQNYGGMDDEISFHAKCSNAYMLVYIRQSE<br>LDRVLGDITESEISSDLVERLDLEKRIEMARRKERGEANTYVSVHVILEE<br>NFEEQHKRRLFDLEKVHPRVFRIKQNQTVDELVDLFVRGFGVSRQRMRMW<br>NLCTAQTQKFSHFDFVAEGSRTIEQISTSQKPWVIWLQLAWTDVPGPLPP<br>FNPKTESLLFLKYYDPRNKRLNYIGCTQQPHTRRLIDLVPDVNSKLGFEP<br>DTELTIYDEYADKKLVNLNEPIESALFIPQDHLQGHILIFERENVDAKLD<br>LPTVGDYFLDLVYRIEIIFSDKCNPNEPDFTLELSNRYNYDQLANAVAER<br>LNTDPQKLQFFMCINNYKETAGNAVPYTFKGTIKDLVSYTKQSSTKRIFY<br>QRLSLSIHELDNKKQFKCVWVSSDLKDEKELVLYPNKNDTVKGLLEEAAK<br>KISFAENSRRKLRLLKISNHKIVAVCKDDIPLDTLLKSNESITTAQGAQK<br>TFRIEEVPAEDMQLAENEFLIPVAHFSKELYNSFGIPFLTKARQGEPYGA<br>LKQRIQRRLNVQDKEWENYKFCVISMGHNADVNDNTPVDLEVYRSWTSGQ<br>LPFFGLDHINKSRKRSSLNFSEKAIKIYN

**CG7023**	MGANVSQLEREIGSDLFPPNEHYFGLVNFGNTCYSNSVLQALYFCKPFRE<br>KVLEYKAKNKRPKETLLSCLADLFYSIATQKKKVGSIAPKKFITRLRKEK<br>EEFDNYMQQDAHEFLNFLINHINEIILAERNAGPSNGNPKATNQGGSTSA<br>MASSIASKSSSTSNSNSNSNSTTNSNGNSSNSTGSLNANTSVLDASGSLT<br>ATTTPIISGNGTGTNGANSEPTWVHEIFQGILTSETRCLNCETVSSKDEN<br>FFDLQVDVDQNTSITHCLRCFSNTETLCSDNKFKCDNCCSYQEAQKRMRV<br>KKLPMILALHLKRFKYMEQFNRHIKVSHRVVFPLELRLFNTSDDAVNPDR<br>LYDLTAVVIHCGSGPNRGHYISIVKSHGLWLLFDDDMVDKIEASTIEDFY<br>GLTSDIHKSSETGYILFYQSRDCA
**CG5384**	MPAFKVKVKWGRELYTDIVVNTDEEPILFKAQLFALTGVQPDRQKVMCKG<br>GILKDDQWNLQIKDGAVVLLLGSKESVPEVPATPVKFIEDMNEAEAATAM<br>RLPAGLTNLGNTCYMNATVQCLNAVPELRTALSTFSNDGTDTMSTAFSIS<br>SAMKSIFAQMEKGTTVTPIVLLQALHRASPQFAQTGENGTYRQQDANECW<br>AEILKMLQQKLRPKNQEPSNTVQKRHSSFIDQFFGGTFEVKMSSEEDPDE<br>PSTVTSENFLQLSCFISMDVKYMQSGLKSKMKEQLVKKSETLGRDAKYIR<br>TYLVSRLPAYLTVQFVRFQYKGKEGINAKVLKDIKFPIDFDAFELCTPEL<br>QNKLCPMRSKFKDLEDKKMEVDVVKRNEPNEEKKDVKYEQFWFDDDLGSN<br>NSGYYTLQAVLTHKGRSSSSGHYVAWVRSSGDVWFKFDDDEVSAVATDEI<br>LRLSGGGDWHCAYVLLYAPRRLEKL
**CG8494**	MSLSSNPNPHNPYVEHRNRTIHSSQGHTLANGYAMGDPRSSRCHDKLFSG<br>SLKSLARREDSNASDSEGTDTAKGSGTGLVGLQNIANTCYMNSALQALSN<br>LPPMTHYFINCSDLVEYIAEQSARRCKPGGLAKSYRRLMQEIWQDVDDPK<br>EFIAPRGILYGIRTVHPMFRGYQQHDTQEFLRCFMDQLHEELTEQVSMLP<br>QTQNQPQYQSLQQQQPSETDDENDDEAAPASLSHASESEYDTCESSMSER<br>SAEVLLKTEYFVTPCRTNGSNSGLPEGHSVQLQQAPLQHQQKNASSAEQK<br>PIEAARSIISDVFDGKLLSSVQCLTCDRVSTREETFQDLSLPIPNRDFLN<br>VLHQTHSLSVQSLNAAETSARTNEGWLSWMWNMLRSWIYGPSVTLYDCMA<br>SFFSADELKGDNMYSCERCNKLRTGIKYSRVLTLPEVLCIHLKRFRNDLS<br>YSSKISSDVYFPLEGFDMRPYIHKDCKSEVAIYNLSSVICHHGTVGGGHY<br>TCFARNTLNGKWYEFDDQFVTEVSSELVQSCQAYVLFYHKHNPQMKLVRD<br>EAMTLSTSHPLCDSDIQFYITREWLSRLATFSEPGPINNQEMLCPHGGIL<br>HSKADVISQIAVPISQPLWDYLYRTFGGGPAVNIIFECEICKRAAETLSR<br>RQQYELNEFTKYNGLQNEFDSTAIYAIAMPWLRSWQQFSRGKTHKDPGPI<br>TNEGIAAPTENGSATVSCVRLGSDYAQLNARLWRFLHNIYGGGPEIILRQ<br>ALSDEDDAEEIEIIDQDDECDDDEDQDLEGEGEDEEIAAASYQHNHSDTE<br>SNLGIRNTPSPSPSTSPSPSLTGTRQPMETESDLRPSSSKSKRSRSKMKV<br>SALRLNMRNRGKRNRSAFKQYAEMFGAKGNYNAHSNAEPVVSEKDEKDKD<br>NDRTVAFPSEYSLPISIPFQSDNFQVNGVHEKSRDKGKLRNSSKSGSTAA<br>TKENVTLQKFVTLREANGPSDETDI

**CG3016**	MESEKILMAAGVTVAAVVGAFVFWGPSGSRLRQRRGQIAGLHNFGLTCFL<br>NTLLQAMAACPQFIAWLQLYNNASPDRKSLITSMLNTLEVVNGTHATLRG<br>DPYSPGAVLRALNALGWVIPQEEHDAHELFHVLLTCLEEEAIRPQPLGCL<br>SDALPTDNDDNSSLAGTATPVGGFRSFSSMAAGLGASQRIGDQPNRPSSA<br>MLTDFLNMEYDESTSLQRLVRSEAHTPDSPASVCERDGNDRLGSVLLDAV<br>SPGTPFGFPLVSNPDSLATPMLGGERSSRPRLPQSQQQDEGLNRRVSSS<br>CRSLERLHRGPGRVSIWSNMMPSQVAHPFQGAMGAQIVCNGCGSKSAVRY<br>DKFDSITLNLPPQRRTGLSLGHLLSEYITSEDLSDVKCDSCNETTTHTKS<br>VTFAKLPACLCIHVARTVWLPTGQVCKRKDYVHFPESLSMAPYSFVQPHL<br>NSQAGTPWGSTMSLYSSSLPMNNGVGGGEGFGTMFPKNLYRLLAVVVHSG<br>EANSGHFVTYRRGSLRNAHRWYYTSDTIVREVSIDEVLSVPAYLLFYDRG<br>QQRQLNLR
**CG5603**	MNSKSDYEAEKEKKLTITNRFGDSLENYELSNESEHKTHVYPKKIPNRKN<br>GILKNTDANHSAVDNQHLEDVDLADILGTNWPKRAGPAAMILNNKSKTDP<br>SNSVDLILKPASPILKIEPEEPLRFTIADYQPLIEIPGTELAIGSLVEVS<br>NPGVCEDLYGVVRWIGIPPGPQKNVLVGIEVEDESNLKNVVASDGRHNGV<br>RLFTCHDGRAIFVPANRCTADRRFADVDNSISANRVSSNHAKKFGVADCP<br>AIYGSIPPLQIHNSDELASICGKFKGIQGHHNSCYLDATLFSMFTFTSVF<br>DSILYRRPGPQDIRNYSEVQKVLRDEIVNPLRKNVFVRSDRVMKLRELLD<br>QLSSVSGLTCEEKDPEEFLNSLLSQIMRVEPFLKLSSGQDSYFYQLFVEK<br>DEKLTLPSVQQLFEQSFHSSDIKLKEVPSCFIIQMPRFGKNYKMYPRILP<br>SQVLDVTDIIENSPRQCSLCGKLAEYECRDCFGSLQAGSGLECTAFCPKC<br>LKTFHSHIKRTNHVSKKIYSPKEFKIMAEHMVVPRLYMELFAVVCIETSH<br>YVAFVKSGSGPDAPWCFFDSMADRKGEQNGYNIPEITCVPELTQWLSEEG<br>ARSINETSTNDKVLPEHAKRIFCDAYMCLYQSTDIMMYH
**CG3781**	MESPSARTLGNSLGDDSGNGNENGNGSGNGNTTMMPHGIYHERQTRHLCG<br>LHALNNLFQGPDMFSKSELDDYCTTLTPRNWLNPHRSWIGWGNYDVNVIM<br>YALQQRNCEAVWFDRRRDPHCLNLSVIFGFILNVPAQMSLGYYIPLPFHM<br>RHWLALRRLNGSYYNLDSKLREPKCLGTEQQFLEFLATQLQMDHELFLVL<br>DEETDCKDKSQQRWLLPQFRD
**CG9448**	MCDTKDDAQKWKCETCTYENYPSSLKCTMCQASKPLLNEDIFRLSPAQES<br>CTVAEEAAAVEVAVMSPTPSSTCYSLQPQSQARQSNVADSEKWPCKVCTY<br>LNWPRSLRCVQCCTKRGGEAIERGKKDMDNEADGDRAGEALQALRISGSE<br>ENLANKPVQLIGATASHRLSLSRGIDDATHLNNLANASHNQSQSQHRQPV<br>LQQQMQLQLQPQQQRESSSSAAVPPQQQKQCYVSKWACNSCTYENWPRSI<br>KCSMCGKTRERЕISGSQNDLHASSSLNSQEENQQQLQQPNVDTVSVNNSF<br>NKKHIYQLGSSETINNCDTLQERQERRQRQIRRQVDWQWLNACLGVVENN<br>YSAVEAYLSCGGNPARSLTSTEIAALNRNSAFDVGHTLIHLAIRFHREEM<br>LPMLLDQISGSGPGIKRVPSYVAPDLAADIRRHFANTLRLRKSGLPCHYV<br>QKHATFALPAEIEELPIPIQEQLYDELLDRDAQKQLETPPPALNWSLEIT<br>ARLSSRMFVLWNRSAGDCLLDSAMQATWGVFDRDNILRRALADTLHQCGH<br>VFFTRWKEYEMLQASMLHFTLEDSQFEEDWSTLLSLAGQPGSSLEQLHIF<br>ALAHILRRPIIVYGVKYVKSFRGEDIGYARFEGVYLPLFWDQNFCTKSPI<br>ALGYTRGHFSALVPMEPFTRIDGRRDDVEDVTYLPLMDCELKLLPIHFLT<br>QSEVGNEESMMRQWLDVCVTDGGLLVAQQKLSKRPLLVAQMLEEWLNHYR<br>RIAQVITAPFIRRPQITHYSSDGDSDEE

**CG4603**	MTGSFSVKLKSKKGQFIVNDLNEHTTLGELKTKIVQATDIEATQLHVLVG<br>YPPKPLDLSQQQEQRALKAVGINSGETLIVEEKAAPAPAAPVPGGTTVED<br>DEALARRLQAEEEAQLLQETAGGPVAQAADYQLPVAPTESGPNGDFNGIL<br>LKKVVPADNSCLFTSIRFVLNGKVDNEGSEMMRHIIAQEVAADPQSYNDA<br>VLGKSNAEYCAWIQKADSWGGAIEVSILSNYYGIEIDVVDIQNAIINRFG<br>EDKNFGLRVFLLFDGIHYDPLYMETSPSAAPATIFPVEELGVYQQAEQLA<br>NEAQSSRQYTNVDKFTLRCMQCDVRLVGQVQAQEHAKQTGHKNFGEI
**CG4968**	MEPFTHNDGNRDELIIQQKRDIEKEISDTTPLVSEQLPLTCLYAEYSGDE<br>IFTAKIQDLSKKYKFIRRTRPDGNCFFRAFAYSYLEYLISNTSAYQEFKK<br>LAEESKEKLVQLGFPSFTLEDFHETFMEVIQRVSPDNAGGHSTVQDELHK<br>IFNEQGYSDYVVVYLRLITSGKLQEEADFYQNFIEGDLTIEAFRHLEVEP<br>MYKESDHIHIIALCTALGAGVRVEYLDRGEGGTVKAHDFPEGSEPRIYLI<br>YRPGHYDILYPN
**CG2224**	MSKAVNNMSMGDVEPQERMKHLSHCGNLIEVDKNMPVTRYYRSGTEMLRM<br>ANVYLREGNHENAFILYLRYMTLFIEKIRQHPDYGSVKAEVRDINRKIKD<br>EIMPTTEKLRAKLLTHYQREYEQFLASKEAERVKELERERERERERQRQK<br>EREKAGSSAIPSLIPANLHVLIDEGNQPSAPDLGLLDQVVYPNDFPTGAN<br>RSLPGSGLLLPAASEAAADKTTNSKPSFDRNQKPSYNRTDSLLAGSLRLV<br>YVPGDTMEVFLKLALANTSKNIETCGVLAGHLSQNQLYITHIITPQQQGT<br>PDSCNTMHEEQIFDVQDQMQLITLGWIHTHPTQTAFLSSVDLHTHCSYQI<br>MMPEALAIVCAPKYNTTGFFILTPHYGLDYIAQCRQSGFHPHPNDPPLFM<br>EAQHIRMDNQAKIKVIDLRR
**CG18174**	MDRLLRLGGAMPQAAPPTDAPVVDTAEQVYISSLALLKMLKHGRAGVPME<br>VMGLMLGEFVDDYTVQVIDVFAMPQTGTGVSVEAVDPVFQAKMLDMLKQT<br>GRPEMVVGWYHSHPGFGCWLSGVDINTQQSFEALSERAVAVVVDPIQSVK<br>GKVVIDAFRLINPNMLVLGQEPRQTTSNLGHLQKPSVQALIHGLNRHYYS<br>ISINYRKNELEQKMLLNLHKKSWKDGLTLSDYNEHCSINEDTVAEMLDLA<br>KNYNKSLEDEEKMTPEQLAIKNVGKQDPKRHLEEKVDKVMQNNIVQCLGA<br>MLDTIVFK
**CG6932**	MEQMEVDVDMSAKPSTSSSAAAGSSMAVDKTADQNPQPQGNIMAAAGTSG<br>SVTISLHPLVIMNISEHWTRFRAQHGEPRQVYGALIGKQKGRNIEIMNSF<br>ELKTDVIGDETVINKDYYNKKEQQYKQVFSDLDFIGWYTTGDNPTADDIK<br>IQRQIAAINECPIMLQLNPLSRSVDHLPLKLFESLIDLVDGEATMLFVPL<br>TYTLATEEAERIGVDHVARMTSNESGEKSVVAEHLVAQDSAIKMLNTRIK<br>IVLQYIRDVEAGKLRANQEILREAYALCHRLPVMQVPAFQEEFYTQCNDV<br>GLISYLGTLTKGCNDMHHFVNKFNMLYDRQGSARRMRGLYY

**CG9769**	MSALNLTVRVHPVVLFQVVDAFERRNADSHRVIGTLLGSVDKGVVEVTNC<br>FCVPHKEHDDQVEAELSYALDMYDLNRKVNSNESVVGWWATGNDVTNHSS<br>VIHEYYARECNNPVHLTVDTSLQGGRMGLRAYVCIQLGVPGGKSGCMFTP<br>IPVELTSYEPETFGLKLLQKTVGVSPAHRPKTVPPMLDLAQISEASTKLQ<br>SLLDLILKYVDDVIAHKVTPDNAVGRQLLDLIHSVPHMTHEQFTQMFNAN<br>VRNLLLVITLSQLIKTQLQLNEKLTFLPTA
**CG3416**	MPSQEVSVNKVIVHPLVLLSVVDHFNRMGKIGNQKRVVGVLLGCWRSKGV<br>LDVSNSFAVPFDEDDKDKSVWFLDHDYLENMYGMFKKVNARERVVGWYHT<br>GPKLHQNDIAINELVRRYCPNSVLVIIDAKPKDLGLPTEAYISVEEVHDD<br>GSPTSKTFEHVPSEIGAEEAEEVGVEHLLRDIKDTTVGSLSQKITNQLMG<br>LKGLNAQLRDIKQYLQRVGDSKMPINHQIVYQLQDIFNLLPDITNDQFTG<br>TMYVKTNDQMLVVYLASMVRSIIALHNLINNKLANRDAEEGKSDSKEAKE<br>KNKDSKDKDNKETKDKDGKKAEEKADKGKDEGGKGSRK
**CG5505**	MPVSMAVCETANVVNAALRESLGGNSSAGSSTDQAKSGEDTNGSLQNHIV<br>ANAKRILMAKIEYEEVPNYHESVLENLKSKYIVIKPGNPGAINGFSGKNN<br>TGKLVGANGHDNNGARKQAEHPNNQSHHINHHNHQHPTSNPNELPKPKRV<br>LYPRENIRIGWKQSERKWQVGTGMINVGNTCYLNSTLQALLHIPALANWL<br>VSEQAHLADCNVAEPGSGCIICAMTKTLLATQSNQSAVRPFLIYSKLKQI<br>CKHMVVGRQEDAHEFLRFLVEAMERAYLMRFRNYKELDQLVKETTPLGQI<br>FGGYLRSEVRCLSCNHVSITFQHFQDLLLDIRKADSLEDAFEGHFSRERL<br>EDMGYKCEGCKKKVSATKQFSLERAPITLCIQLKRFSMIGNKLTKQISFK<br>SRIDLSKYAARSQAAQAQPLTYRLVSMVTHLGASQHCGHYTAIGSTDTGS<br>FYNFDDSYVRPIAMHSVCNTNAYIMFFELDLSQAASPAANRPNGVRLTNG<br>HSTTPVPAATVSSPSPTRFIGPQLPAGGANGYTNGNAQKTAIQFKQQNQQ<br>SPQNGLQLGTGKFQDTAKPPLVGAHAKGEATSAPTANGNKSSSPSSNSSS<br>NHKSINQQQYLPISSDDEDIEDEMKPRPTTAQLPSMPNMTENHTEPKAKS<br>PVKIQVKTPVKTPLKSLVPYESASEEEEAPLPNPRKRPSGEDSSESDQES<br>GQTNGHSKTNGSHTNGSASSSVHVNNSKQKTDAIDEIFKSLKKSADSDED<br>DDEEEPSIQLTNGWHPQKQSQSQSKAPPSPKTPPSPAVIKSKTGIWKVTR<br>NDEVDAIEDDVDVVVVEGSPVKIPTPNKNHRNPFSSSKPSTDSPATPGAK<br>RQKLLNGSALKSHQQPRVGNGYQSNATSNGSTINELLKQSYRGYGSPVLS<br>WNGKPAELEKETFELVCAKRIAGHGSVEGSDIVEGSVAVDAAVTSGSDSK<br>DVVVIAVALLVDAREQRQRDIDDDEENEMDRGRQRKVKSGSAKGNNASNS<br>TPGYNPFQEYEGQKRWNKNGGGGGFPRFYNQNYRQNFQQRNKFKFNRFGG<br>PGSAKFQQQRALQRHLSAGGGFSRRQPSAQQQQQT

**CG5486**	MTDKESEQCTVSVFDQTPGSEQKKINVVVRSHFTVKRVIDLIGTQFSYEK<br>FELLLQPHDNKDLVNLNALESQLMYEVAGFEPQLKNHLILLPSGSWDGDV<br>TKRFELPIKRVVVKKVMKSDGEKAKSPATGEKKKRVVGEKTKKKPASGSS<br>SPSKAKTTSEDSLAKTSISSESSPEKTSKIKTTAAKISKPGSEKAPRASP<br>EECPELSTEINSKNTSSESPVAKKTAKVTSKPTLELLSPIKPSSPIKELD<br>CEPVDTLSKQQLSEQLQLYPQGRNLISPVDDAPSDLFISDAEQLSDDDLA<br>LGASASPTMLGPGYDYGAPTGDSDVEGVTGVTDPSTIGTDDGTYPALSNF<br>YRRKYGGDELRAWQRVNTTGADFVSSATTETEAEARQASLGPRGYVGLVN<br>QAMTCYLNSLLQALFMTPEFRNALYRWEFDNDNEAKNIPYQLQKLFLNLQ<br>TSPKAAVETTDLTRSFGWDSTEAWQQHDIQELCRVMFDALEHKFKNTKQA<br>NLISNLYEGKMNDYVKCLECNTEKTREDTFLDIPLPVRPFGSSSAYGSIE<br>EALRAFVQPETLDGNNQYLCEKCKKKCDAHKGLHFKSFPYILTLHLKRFD<br>FDYQTMHRIKLNDRVTFPQTLNLNTFINRSGNSGEQNSQLNGTVDDCSTA<br>DSGSAMEDDNLSSGVVTTASSSQHENDLNDEDEGIDMSSSTSKSAKQGSG<br>PYLYELFAIMIHSGSASGGHYYAYIKDFDNNEWFCFNDQNVTSITQEDIQ<br>RSFGGPNGSYYSSAYTSSTNAYMLMYRQVDAKRNELVAKVADFPEHIKTL<br>LPKLHSEEETRVSRLGRHITVTDLALPDLYKPRVYFYNPSLKKMKITRVY<br>VSQSFNINLVLMSAYEMLNVEQFAPLSRCRLVAYNSSMDTIIQSLESCTD<br>PALTELRAAQNYSLDFLLEYRAEDQEFEVYPPNGITWYVFKVDLSTMAMD<br>GPFLVYSAAREREASDVLRRSIALRLHISEQQFLLATVRATVPKAFVSYD<br>PHPTPEALQHLQNMANTQFKSITYFYLNVPNTDAATLEMLGVPTVESVEC<br>ASGGDVVDAAMMNGVAPGHMSSSNDYDWRRYKRDLVEPMSQPSPSHGHES<br>NSEDSSLSDGDRTLVETDNMAHRGGGDSQVSSTSHSPQLSSPEDEAASHD<br>AMMRVHAYCNGNGSYAAADVVDPLLLPTSTNHFFYATKVECVDVVGTGSS<br>SGHQSDEEAQLRKPTRAYKLLVGTHMRMGAFKKHIEQLIQVPAAHFKLQR<br>KHDNNLSNNQNNSLVHLIEGETLTVELGKTLEPDEFKAKIHFLRLADIDN<br>ETSKLPCVCEWVYNANTTAEQAKKELVAKLHRIDAKYATLSVQNCRIWLK<br>GGRIPIKILSDDETLYCDMRSSIAAEFIVQECEEEVDPQPKDDSLTLFVR<br>RWCPAKLEFGKFQEITLDQDSEIRLSLSQISDIPIDKLSYMKLNSNFPCT<br>SISALSVNESSSWYSVPTTLDKYPLNSTQTGNIYLYKDRTVPARELTLEE<br>RRLMNAREKARLDRVGCVSTTRYAQRRERALKIYLDSPEKSSNVTASAPM<br>DVHVNN

CG5794

MCEVCADFQNLLELYEVRVASSDLKFQLLLKSEIETTFNYIQSWPQRQCM
CLYRDTKNYDRFNLVVQSLICLTVQHLKHIDHLIDNYKRLTASAAQVVAQ
AQQQREQQRDEASAQAEAKESSAPAEEPKKEEPSGSAGEEAQGSGDGPAK
KPPVGPCTPPPPQTANPQHKSHLQYTEEPWILPEVEKLLVLVSKVFLLNF
PLYIAHKHGMHSRLDDLQAEEAHHLALICDLHDNDLPIYLLRNVSLFCNS
GGFGAMSLCFEHPDLPVSTAHSMTAAVSNVKLWLNYHCNTQLFVPLRSRI
LQYMCKLSDQSLRSAATRAMADFVWSSMRDPLDVAVNFDTEGLALAFKYF
TSTTLTMRLAGMAQINAHINLFNEICTTETVNEVELFGQRIANWLTENHI
VQHLFGPNLHVEIVKQAHVLLNFLAVENQISEEDIKLIWQATQLKHCSKT
IFDILPSLVKNLTPRPAMHLYSLLCRMDPKEHTEQSIYIASALTKQLWTR
DTSRSQMNLMQDHLLGSNVTASSSDSGSIEGSNTEDDHVGADDSSIASGG
GGGGVGNKSPIDGVTPCKQARHRRHICDPTTEKGKQISPEDMAKVDLVNK
RIVNIIDNTSSEEELQSRAALELQLHRSRKKTSNKRRRQKTNKQIILPHE
LVEIWDGVEDVPSSDEADGEADGDGEGELLADSDECSDGATSQLVPDAVL
KHLQGEGPFIRAIETNINELLSGAENDGSYSSPMSNKSEKNLADFDDEDV
SPCEEELAQLVSSRANCSDVPPAFAAAAAAMMVAQSAMALQKSGESNVAA
AAAAVAAAAAAATGTAQQTLVAMNRQASVAAAAAVVAAAKAKSDSDVDLMD
VVSGGKQHHSPKASQGSSTSGSTPVQPSFKLNDVCQPGNTLLWDLLQDDK
IGQLGESLALEAEKALATLLCFSMDRQLRTKFIEGCLYNVANNRSVIVSL
RLLPKLFASFQQFRPSDTHSMTMWAERNHRMMQCFFNNIRHYARRHAEVL
ITQNGEQQQQQLGGQLYSHKTQVSVRLQFLSSIFSTVGSPKSFRLTLEQL
DALWEWLAHDPECADCYFSWLQAQAKGGDQHALGIEALQHLYLKKLPELR
PEEFSMVALGLFQQLCSFARIAMAEYDNHSDQISASASAVGMYHLWKIAL
RAQSNDVSLAAIQYINMYYMGQQLRLEKEFVSQCMENLVQAATALESIDD
ENALMRVQRGLLLLNTHLDTFRRRYAFHLRRWAIEGKGIGSHSNLKNEGA
GPPLRIVLQQAGLSEKSLLQMHACDLIADLKAEVSKWWESLQTGLAAPVL
GLLLSDGPLRIITQGQELTSDYDERSLGDAGFKDNQIVYVSLGGRGARRK
ESNLEHPSMLPPPPKECLPTVLLLQPKYFEKLFCLMQTLGDMQPQASTVN
PQHHTKAQLLSRRVWDILAMLPTNPHILDAFKSLVTDLSELEQLDAGGEE
EQLATKRKQIKQKFRDLLDPNNLQKFMYSLHIVESLALTSSRRGESNGNV
AMGNTPEQVRVKKSSMGRRRNSNEQPPPPPPEVKMSKEQLCELEAPLTPT
PSTGLQDVETEASSSSGGDKENQPKQHSKRQKKGETFEQEKERPVGCSTP
PSPTPPPPALSVVERGDNKWSEAFVKCGGLRHLYEIFSEGQLQQSAHPKE
LALNEWRHDCLASLLRILWLLGFEELQSADAHVLMSRPHPFMLQLMEVPQ
CLTRLSSILNDEVHQQHQASSANPLVFPYQFQHLRTGFWGRAQLIQFAMN
ILVSFVHASAEARRLLWAPTGTDHCRWLQKFILEDPEPAVRREICAGLYR
ICLGNAHSYRLLLAPLLHKLIALLPLAEQMSSGNQHTQFLLSEEGKDPYG
PACRDYFWLLARLVDTLSPEMVAEEHIDIEMLCESISQSILTREYYELRH
GYQDDGLVGLLNLMSNLIKYDTTFKYTPKALSFIEQLIGFLFDMPSPADR
QKPKCKSASSRASAYDLLVELCRGCATNYAYLHGRLLAQHKSGPKQPYPW
DYWPRDEGRAECGYVGLTNLGATCYMASCVQHLYMMPQARAAVLRVPPNA
ARKHGPTLLELQRMFAYLLESERKSYNPRSFCRVYQMDHQPLNTGEQKDM
AEFFIDLVSKLEDMTPDLKHLVKRLFCGSLSNNVVSLDCGHVSRTAEDFY
TVRCQVADMRNLQESLDEVTVKDTLEGDNMYTCSQCGKKVRAEKRACFKK
LPQILCFNTMRYTFNMVTMLKEKVNTHFSFPLRLNMCHYVEKTLMPQQYK
EERERRQKEKEGADGSGDGNDNEKAEATLDDDIEECYEYELVGVTVHTGT
ADGGHYYSFIKERTKTSYHTHERWFLFNDAEVKPFDPSQIAAECFGGEMT
SKTYDSVTEKYLDFSFEKTNSAYMLFYERRLPEHLQRRHSELLVTPTPSP
TVEEKSEAEEPTKMETSSSEIKADVDVEVEVEEKDKEKPAQTDTESKETP
AKEEIADDKSKQDEPEEKKIEKQSREGEEKSETDEKPTEMTTVSTEEEKQ
PTANCDNHQQNNNSNSKASNDQQPSTSKAAQKLQLFRPLLNKELEDWIWQ
DNRQFLQDRNIFEHTYFNFMWQICGHIPQSLISETDVTCMAAKLSVSFFI
ETFIHAKEKPTMVPWVELLTKQFNASQEACEWFLSHMSQEPYWPVQVLIQ
CPNQMVRQMFQRLVIHVIQQLRASHAHLYLEVETDEDDKELIGQASCVTR

**CG4165**	MVKKRQADSRDHDCSTDSGNEDLHHRKGLGSPGQSDGATPTTASCQHIKK AVDAARLRRLLKSTGLLYECSQCQKLGKTAGSAAGAGASEGAVGPGGNPV TFEFDNTLWLCLKCGSQLCGRARHKHALEHYQTPHSDSHALAMNTRSFDI WCYECDMKICSNLRKNLLECVELVKKLAQKPPTSTVTPSTPTISYIEEKL KAALEHLTPIVPMTGGSFDDSSSRGSLAAAGGGGGVGSSRNRQVAIPMPP PEPSSGLSTSDSLTSVPGMAKRIDQYSATTNGNTGNKRLLTLETPRIENE RLPRVRGLTNLGNTCFFNAVMQCLAQTPFLLSVLKELSEPGEEFILPGGT FTIKDKGDIELPMIKGTLSSWGGLTAALANALEELQAGGSVFTPRKLFDR LCVKCPQFTGGDQHDAHELLRQLLESVRNEDLKRYQRVILQNLGYKDQDV NSVSEEMRQKCKIYGNQAGDRILRPEQVFRGFLVSTLTCQDCHNVSSRHE YFLDMSLPVAVEKPQPPQRRKPSPELSLTSSSSSVTPSTGQPTINTKFTD GSVNFTASSPSFFLHAHEAASLGPSKSQVKKEKERQRKAKRAAKKRQKSS LNLNGNDSGNGNELAESVDQDDASLASLGAGDGQANDGLEQTNGQTEDST TSSVTTSEHSDADVEDNLVEDTAAPSTNNVPSSTASLTAPSKTYMDSNGN AQPPGEKRDDTPEHMDKDSLEEDENDSGIATSPAPTATNSSTSTSATGNN NSVAGSGLSGSSGALEDPLAPASLVTAGLSEKGASVIRQVSVGAEQGASN GTEDADGEAKAIEQPEKTPSQAQAMAQAQARTKRVRTQSYSDWSTTIAPR YQCEDGECSVQSCLNNFTAVELMTGQNKVGCDSCTQRINGSDPKAKSVNT NATKQLLVSSPPAVLILHLKRFQLGPRCIFRKLTRPVSYPNLLDIAAFCG SKVKNLPNIDRKQKKLLYALYGVVEHSGGMYGGHYTAYVKVRPKVAPGDK RWKFLPHGSKAELDQDDDQLKKLEELLAKEKAREQHLKVLDDSDDFSNSS SNSSTSDESQTPATPLEEQQTQQAQQPQQPQQLEEAANVRAPPGKWYYVS DSRVQEVSEDTALKAQAYLLFYERIY
**CG7288**	MAQTQAPAAKRMKLEKTPANPVEEDETPSVFNPKYRVCPYLDTINRNLLD FDFEKLCSISLTRINVYACLVCGKYFQGRGTNTHAYTHSVGEAHHVFLNL HTLRFYCLPDNYEIIDSSLDDIKYVLNPTFTRQEISKLDQLQPKHSRTVD GVLYLPGVVGLNNIKANDYCNVVLHALSHVGPLRDYFLQKQSYAHVVRPP GDSVFTLVQRFGELMRKMWNPRNFKSHVSPHEMLQAVVLWSSKRFQITEQ GDPIDFLSWFLNTLHRALKGNKHPNSSILYKIFLGEMKIYTRKMPPVELD DAAKAQLLATEEYKDQVEDKNFIYLTCDLPPPPLFTDEFRENIIPQVNLY QLLSKFNGTAEKEYKTYKANFMKRFEITRLPQFIILYIKRFTKNTFFLEK NPTIVNFPIKHVDFGDILGMRQRDKDVKDTKYNLVANIVHDGDPKKGTYR AHILHKANGQWYEMQDLHVTEILPQMITLTESYIQIYERCPNSS
**CG7857**	MCDGDVAIAELESRLDEVSLEDIGARHRRERKDLQAKLQAMKKNAPKNNK NKRKEFLEEMARLEGELEQRHKAELKAAEAMEAPVLVEPVVKEPAEKPET EVTDDDGIEEKEEQLAPNQRVSKAQKRRDKKAKEARAREAEIKTELQNAA NQPTPKLIELQQITAKLSQRQLSLHNIPSDGDCLYQSIRHQLIVNALPGH SVQELREETANYVRAHKDSLISYMIHPETGDILNDQQFEQYCHDIAKTHA WGGHIELKAISSLLRVPIEVIQAEGAPTLLGQEEFGGSPLIICYHRHIYQ LGAHYNSTVPAA

**CG15817**	MHEYDVNERKEEPASLPPTQNETTEINSQLRQDQQNQEQENPLRQQDNSH<br>PPQENPINQQEKDTLQVSVLYPLVVCAQLADSQLVLTADTPKLSPVKKRQ<br>RRVYVADTLRCYSLRSRQEPATKDPLEGGLKQPDVVPPPPPVQAPSPTPT<br>ESTEYTPPEQFKSEFWNRIEEAQEPKPVGKKRRTPGGLKRTRGNKLLKEL<br>EIQSPETAAAIAAAQAELNAAGKSGAAGARSSRKRTNTMSLYDRRFQTTT<br>EERRVANGYGGNAARSVTGSSSNEIESQEPQSHQYYGPNGGSNAAGAGAG<br>LNGSAAALNHAPSMGTLCNIGNSCYLNSVVYTLRFAPHFLHNLHHLIQDL<br>NVVQQTIVRQQTARSASLGKNVSAAQLEHARSWSSKDLATSTDQYSGQNG<br>GVNSGNGGSGSSKSTHQSVTEKLHELYNNLHGNEMADSTEPYHADTLLHA<br>IQDVNATFEGNQQQDAHEFLMCVLNCIRETNQSLIKAIGECPEVIANGYI<br>ANPDEVDTGEGQDRTDSTASQNLNAGNGSLATSQTTTTKTSFFSRKSKRK<br>DEVKPSKSTRVQSPLKENSPTAGGITGAGTAHATANSLFYLNTVDLSGAS<br>STSGSASTSASGVVSTSAALPTPPQATKYSSDDEMNSATVLKDKMRLEER<br>IRELNLNFFSSDFEGIVVLTTKCLSCETITRQKQGMLDISVPVPISGYDN<br>ADLQDKPSTYIQNSCITKEYFRGENKYSCNQCTGYTEAIRSISYEVLPRL<br>LVIQLNRFSGGMEKVSTYVPTTFTLPCFCATCCELGEGNKLHVYKLYSVI<br>THVGATLTVGHYIAYTCFLDLASDYVNCPKDRRNTMTNSQTMASQAVPSN<br>ENAAPNNGSSSVASTPVIAAASALASSGNILMKKMKFGRSKASSSGDMSK<br>NVKQVNGIISKNITNGIGKLSISTTCQGVNCCAMRLCCTQQTSSSNSTSC<br>SDFSEESLQNGSNSNLSFGGSGSTYPTGYGSTGRGGVRANYAHGGPDPIW<br>YMCDDDKIKAMTQREFEELLSPTRKITITPYLLFYARFDLQPQKATPPMP<br>GSSTTPPPPSSAQSSWSNENVSSGSHKI
**CG14619**	MMLDIKKTRGFHKIPQASKLQSTTKTSSVVATSASSQNEVPSPGGSAGSK<br>VGATNPVRAANQRFFLVSSYKDPTFLKAECDLAHAHVTTSKVKTTLQPHR<br>RSRAGEDSRNNNYNTSRAPTLINMRRPSLFNGNQQPTTTNSTTINNTTSR<br>NTTSNTSNGVLKYSVRSTTATATSTSTRNYGKLKPLNNNQTTAGVAMMNG<br>HTNNNNNNTRNSSNINNGGNNNMQRQQQHDDISFIDSDDPPATGGPEAG<br>ISTTKTSICYFKPITPPLQLRHEQNQVQQQEEQPQPSSSKSASHRYPRPK<br>STIIASAHSNFAASFEKFSGGLYRQTNGETNIESKTSSNARRYGIDSLSI<br>KASIEKFNNLSGQKRQNPGSGSGIGPGSATASGLGGGRLSVASRSNHGSQ<br>AGGSSSNLQQRYSSDLDNIRVAAGYSSSLTRAAYRTTATMNSVSTPVAVT<br>SELGGPISGDGGETATAMGQPTSKVTVRGAHSNRQPIECDSVVLTNKASK<br>DATTAATPTVATATATHTPATSSVSTVTVTAAAPNSASDSTLARSGTGSS<br>STARSVLPPMTPTSSRYWDRDSGTSRSSIGTSSALNSSSLKHNSDDGYKT<br>ASSSRDEKSEGLCGLRNIGNTCFMNSVIQCLSHTQELTRFLRSHHGSRSL<br>STKDQQILHEFAKLIQEMWTANVHTVTPMELKRAFSTKHRMYSDYNQQDA<br>QEFLRFFLDSLHSALNSGVKGETLNIDDNLSDNKKADLTWEWYTRHENSL<br>VRDLFVGQLKSTLKCTTCGNTSVTFDPFWDLSVPLPSSSRCKLEACLDLF<br>IREEVLDGDEMPTCAKCKTRRKCTKSFTIQRFPKYLVIHLKRFSETRWSK<br>LSNIVEFPTSDSELNMGSYGANSNSNVHYSLYAISNHMGSTAGGHYVALC<br>KHPVSRKWHEFNDNIVSDALSENHLVSSSAYILFYERT

**CG8830**	MAVKQNNSEAGDNASDNAGGAGNGIPPNAKPMSANGMANAEENVLPKGMD<br>TGSPAGESLNSNRSGKRDIKALLAYLQSIDMPEQGHNVMAICERVIVDLA<br>RLQIPRGASCPRLKEDVCQVGAFLATRNEKFHQMFYLRVVYELITKSPFE<br>PPPSCAVAIVFQLFDSTQILEAVHSLLEQNVQDSSIKKTVNLLCDWITYC<br>TFCSTLNLWVLALLKGLRDQGKMALLDEIAMDNIEKLFHVMIFPALRQKA<br>APVVFHMLSTINQTPEVFHKILPRIPRVLQYVKNQSTTMDEIGLETKKCL<br>QQLVDLTSALMLRFYDQDELYVATKKALQTYEPSPNCVALAKAMHENAQP<br>WGRRNARVGLVNLGNTCYMNSVLQALAMTSDFSRQILLIECNSVLLMKVQ<br>QQIALMHHSLRYELTPSRVLNATRPPSFTPGLQQDSSEFLGYLLDLLHEH<br>EINSSSVTGHSVGPPKTGREVDDVPALLSEDILSSGVIPYNSKDHELSSG<br>SNSDNCNHKPTPTPPATPTKATNGLKQQGQQVDQAKPPSTIDKTFAGKLS<br>TTYRCLNCGWESRNEDSFRELQLSFPDDKEDCGATNYSVQDLIEYYCSPE<br>KLDGDNQYFCPQCKKLCDAERHIGVTQAPKNLILTLKQFKYDQKYHFRTK<br>LMHKVFHDESVTVKMSAKDSLQEMSTVHYDLYAGVVHAGYSMDSGHYFTF<br>AADQAKNWYKFNDNVVTHSKPEEMHNLTSPNTPYILFYKMCGHSNESNSV<br>TASCSSSMVSGSNDQVVSVPLSPPLKLEELPRRLRDYVRKDNHVYNEELK<br>MQRFKRGNSGHGNAFVSRHNFDGDGDEDDKAPPPPGGCGGNGLGMNINRF<br>VF
**CG6091**	MTIKPVSAPPSAKRVAGNVDPDNKEAQVVVVEQSVVSQTHNHAHNHRNVV<br>IDGQSPQRRGEVYDELARTHRCSPHKSTRSKRREHHEAHAHLYKRDRLER<br>EKLVHPTAAAGSGAPGVSGSKCNSPPSTSTSGSSSPSAVGRNSPEHLGLG<br>CTTVPTAQVQMSTSTAAANLLKTVEETFSGYNSGDEHHQPKERLIPVEEW<br>QRRDLEFAKCMEQRGYELKPVEEDGACLFRSISLQIYGDEEMHDVIRQHT<br>MDYIHENREYFGQFVTEDINSYIQRKRARDAHGNHIEIQAISEIYSRTVE<br>VYCYQSNPINIFNSEQSQAGYPPLRLSYQRGSHYNAILDPYNATVGVGLG<br>LAGYKPEIQTKEAVRLSEQLEIEQTMFEDKLKTTDWEATNEAIEEQIARE<br>SYLQWCRENTQRSRNSNTAAGSATSSTVTSAEALTDSDASPSKYSACGGT<br>GSSGNPPVALTSGSGGDGAGPAFSLSPKTLNQFSHKLPQEVNELGGYDSD<br>ATDMSSTSSVGHSGGSSSPSTAASQRSGKGSKSQRRATALRKKRRHETRE<br>TTLASKSAEALEAPLRKSPKRDSAPSVARADTPEAEQRPSTSKQSSHSPQ<br>KNVDSKSPTEQSYSSFYQELLEASYANEGANESEMLQQAIQMSTRDYMED<br>QKRKFLFGP

**CG30421**	MGTTKERREQSEGDAATTHLTTTATATTGAIVAAGVTTTLPATKTTTTAA<br>DTSTTNAAKKIRRTFSMPRNPFRWTRKFKTAVAVTSSAGAENDNESGKNN<br>GARERRGSHTAISSCVGGGRGRSGSIVSLSSYEAPFISSTNNNTTTASNG<br>NNNNSSSSNINSKRTRALRRSSFRKFLNRIAQHLSSTVNVVSEEKHSPAN<br>RIAFIEIVPFYTGCWRRKEANKHGQPSVSGSSSGGGGGAGGPGGGERVPP<br>IGGYQWPADQTPGVMGLKNHGNTCFMNAVLQCLSHTDILAEYFVLDQYKA<br>DLKRRNKINSRKFGTKGELTEQLANVLKALWTCKNESDHSTSFKAVVDRY<br>GTQFRSSTQHDAQEFLFWLLDKVHEDLNTASKRRYKSLKNSYGRSDEVIA<br>AETLANHIRCNNSFVQAVFQAQFRSSLTCPRCEKQSNTFDPFHCISVQLP<br>QLTQQTIFVTVVYMTRLPRQVRMGLRVPAGSPIVALREQLQADTGIEGSR<br>MVLVDLNAEGFTRVFYDTQPVETLSSIETIYCIEVPAATPVAKAATPSET<br>GDKSAPTAVTPSPAAAQSQADLLLLVANVYRAKDGADITRFGAPFSMKAP<br>RDCSYQDLQKRMLREMAPLLKPEVFSYATPLAEMFRIRLQDPSADPDTYL<br>EQVEHPLLTEMIDMALSVLSSEAGPQHIKLLLEWSSPEAHFISKQQDTEE<br>AVVEHESVARLSASKPSDTAALTLEQCLEHYTKAETLSAEDAWRCPHCQQ<br>YLPVVKTLGLWSLPDILVVHFKRFRQHQSKGPQAAKLTTMVKFPLTAFDM<br>SPHLARGVHESASSSLGMGTGIGLGLGLGSNPWKKARSVDSRSSTLNSRC<br>DAKDTRYDLYAVCYHQGDTLETGHYTAACKNPYDRQWYKFDDQRVSKVPE<br>DDIEQDIINNEAYMLFYQRRSVDAGECSGSSSNSGDHWVSRIAPASSSSA<br>SSTSGKEREPVKLEENTESPAAVEVTEKAQSKPVDIVCQDKAKGTADGPD<br>ADQEQTTVPHDLQIEELAFADADVDTSLGETIKAEVNLAAKDEPKKVEVE<br>EATPIFAMDEDGSEARQEKKEECVKKEDELSEAPDVSELTAESKTNGHVS<br>SSSSSSSSESSLSSRSSPRSLSTSVTAASTLQSKFNGHMSAAPALFNGNG<br>NTSQLSSSLQLSRHVLHNHQWHGSRSSVSAVENATQHQQQQAAASLSLRH<br>SFSTSSNYTETLSSLLRKSANTCSKDTLLFIDQQSHHHTAGLIEDDDDSY<br>MGRSLWISPVTPHKLITVSPKN

CG1945

MTFDTRRHTTGQPGSTAPSSSSSTTSTTTTTTSPAQSAGSGSGIGTGTGT
VANSSLPGGGSGSLDGNQDQQPATDSQSSDDVAASLSANSVDSTITIVPP
EKLISSFPTTKLRSLTQKISNPRWVVPVLPEQELEVLLNAAIELTQAGVD
HDCEPCVEFYRNGLSTSFAKILTDEAVNSWKNNIHHCILVSCGKLLHLIA
IHMQRDNPYLLDLLAIVFDPENKFNTFNAGRQPECFAAPDYIWGQLDSNK
MYARPPPEPKNARGWLVDLINRFGQLGGFDNLLERFNIGLELLKRNQNKC
TGKNISVEGRVENGAQDNRLTLALIHSLLRPFGQCYELLMPATIAKYFMP
TWNVVLDLLDSFTDEELKREVKPEGRNDYINGIVKSARLLASRLTGQEEL
IRDLEMFRLKMILRLLQVSSFNGKMNALNEINKVLSSVAYFSHRSQPLPH
CMPEDEMDWLTADRMAQWIKSSDVLGVVLKDSLHQPQYVEKLEKIIRFLI
KEQALTLDDLDAVWRAQAGKHEAIVKNVHDLLAKLAWDFTPEQLDHLFEA
FQASMTTANKRQRERLLELIRRLAEDDKNGVMAQKVLKLFWTLAHSQEVP
PEVLDQALGAHVKILDYSCSQERDAQKTIWLDKCVDELKSGDGWVLPALR
LIRDICCLYDTTTNHAQRTQTSTNRQQVIERLQNDYSLVILVTNSLTAYM
EKVRQMVTDSPGLDATRILIDGRFPHHVQIAERLEFLKFLLKDGQLWLCA
DQAKQIWHCLAVNAVFPADREECFRWFGKLMGEEPDLDPGINKDFFENNI
LQLDPHLLTESGIKCFERFFKAVNSKEDKLKAIHRGYMLDNEDLIGKDYL
WRVITTGGEEIASKAIDLLKEVSTALGPRLQENIAEFHEMFIGECCSRLR
THYGNIVILGKTQLQEELDAPDQSDNTNDESKDSKMRFIEAEKMCRILKV
LQEYVKECDRSFSGDRVHLPLSRVTRGKNTILYIRFQNPGRSIDDMEIVT
HSNETMAAFKRNLLKRIKGTSTANIKVDLFYANDEMIGVSDEINPLYQYT
IRDKMNLTAKLTPVGTGLASSPDSSSDSSTGSPPRPCPDMQRVESESTLP
GVIISQNYQYTEFFLKLYQLGSDLEHGRLRDSAKVLLHLLPCDRQTIRQL
KIMCKVPKAAVTVAVTGDKIAKDEEEKLYPTEQAGIEDEEEHCTPEQMFL
HPTPAQVLYNLSVLHGLLIPALDPLGESALLVQSAWMHSGCAHFVLELLT
KNNFLPSADMHTKRASFQCVLRLAKLFLYIVGSVLSRVGDEPMICDLDNG
SRSQVDILKQNFSTMPSSSQGTLRAISAKLAVILAREMLSASPEGDRCRT
LFSSTLQWSCPDISTIKAVVQLAWASSCGNLQALGNSSGDFEDEVIVPDG
QDFSMCKEALEVLTISFILNPSANEALTSDPNWPKFITSIVLKNPLRHVR
QVASEQLFLASTYCAGDRRPFVYMVNLLVGALKTLVPQYESTCAEFFSVL
CRTLSYGCIYNWPLQISEGLLGDEIKWLQRIRENVHATGDTQVHEELLEG
HLCLAKELMFFLGADSKAQLNELIHELIDDFLFTASREFLHLRRHGSLRQ
DTVPPPVCRSPHTIAAACDLLIALCQLCVPNMKLLTNTLIDFVCTDTDPL
REWDYLPPVGARPTKGFCGLKNAGATCYMNSVLQQLYMVPAVRVGILRAH
GAATTDGEDFSGDSDLTGGGLGSALFSGPASALVSLPSSSSTIEDGLHDV
RKNYHVVILKHVQAIFAHLGHSALQYYVPRGLWTHFKLLGEPVNLREQQD
AVEFFMSLLESLDEGLKALGQPQLMNATLGGSFSDQKICQECPHRYSKEE
PFSVFSVDIRNHSSLTESLEQYVKGELLEGADAYHCDKCDKKVVTVKRVC
VKKLPPVLAIQLKRFEYDYERVCAIKFNDYFEFPRILDMEPYTVSGLAKL
EGEVVEVGDNCQTNVETTKYELTGIVVHSGQASGGHYFSYILSKNPANGK
CQWYKFDDGEVTECKMHEDEEMKAECFGGEYMGETYDNNLKRMQYRRQKR
WWNAYMLFYTRCDQTPVQYEPSVEQLSLAESRNMVLPLPKPIERSVRHQN
IRFLHSRSIFSVEFFNFIKKLVSCNLLSARSNKITPAAEELSLLGVQLAS
QFLFHTGFRTKKSLRGPVMEWYDALSHHIRSSALVRKWFANHALLSPPSR
LGEYILMAPSPDVRTVFVKLVVFFCHFAINDEPLTGYDGANLCEQVLISV
LRLLKSEAADYGKHLPHYFSLFSMYVGLGTREKQQLLRLNVPLQFIQVAL
DDGPGPAIKYQYPEFSKLHQVVSHLIRCSDVSEKCQSSNQNARPLSNPFK
DPNVAHEELTPLSTECMDLLFNRTGYIKKVIEDTNVGDEGLKLLQYCSWE
NPHFSRAVLTELLWQCGFAYCHDMRHHTDLLLNILLIDDSWQHHRIHNAL
NGVAEEREGLLETIQRAKTHYQKRAYQIIKCLTQLFHKSPIALQMLHTNS
NITRHWSIAVEWLQGELDRQRGIGCQYNSYSWSPPAQSNDNTNGYMLERS
QSAKNTWSMAFELCPDEVSEKTDENNEPNLETNMDENKSEPVAQPGGVLE
GSTGGTEQLPENKTPTTSSPSTAAWPARGDSNAIPRLSRQLFGAYTSTGS
GSTSGGSAPTSALTTTAGSGANSETESSAQETTGETTINGLTNSLDQMEI
TAKKKCRRVIIKKLVESKDEEDATTATTAATTEVTTSPATAIATAATLEP

**CG32479**	MASTSSSASTTAGSSGAAGGGAATPISTPSTPQPSGVSSGPGNNGNNNNN<br>HHNNTLVSPSGNNNLMTPVQAFTPTGQPVYNPPVYMSAHGGHPHAGAHYP<br>AMIPAPMPSNHVYVNNVTANVNLHGWPHGVPAYMPPGGQHHYIGHGDMPQ<br>EQGNTVMPHLQPVPINLAPQGGSPNPHGPRMGGGRMRERGRPRGGGPRRN<br>DYHGPRHLQQQQLITLPPQQQLPPPDGANPGLQLQPDQIGQQLAPPEAHM<br>QHLQQYYAAPPTYAYGPYPSPYFTPQHANVQPPPNATAAQQATGTPLFIS<br>GPLMYNPAWFNHGGYIYPMMHPAEYQYVPEDAGQPGVDERGAQPDGGMTQ<br>IWHQGPMYAEDFEALQQQQQQQQVAVHPNGGAVSVADELNHNSSSLPSSE<br>TSSMISPNYPIYDPQMHEMQHQMGVMQIFDDGQMAALQPIHPGAGPLPQY<br>EDELAECGAPPPGPIPVAWTAAMPLPPDAVQPALLPPPPHHILQQTSPLL<br>IEQPAQLVQAQPPQPPTPTPQQQQQQQHQEQTADKSLTQNNNEVVALDEK<br>LANEKQQQIDFHQQQQEQQQQQQQQQQQQLHPLAQQQQQEEQQQPQQLQ<br>PLQTEKPPTKVPNLVVATVAPQLQQPPLQQHQQQQTSPVVVSPKQQQQQS<br>QAAHQNSYHDQHPLPQQQQPQQQQPQPQRKPSQQYNQAQQQQVRRKYSSE<br>YNHHHHQHGSSDTGIQTTKTMSWTNSAQHKKSTQSVSVTASPNSVNNGPG<br>VGAAGKANYSHTTKTFTNQQHYQQQHYHTQNSYNNNSSGGGSSSNSSSSS<br>SNNQPQVRNYGTMKMPSSPVAWTAPTPERKNSQQAAAAPAAATASAAPVA<br>ITTVTTSTASITATPNQFQPETGSSSASPAVAAAQQQQPAAQQHAQAAQT<br>GAAAAPSKSHSNYASSKKHQSYEPVVLSSVVATSSSANPQPQLNLAPPAP<br>PASQNSSDSSQLSWASLFASNKPVAKVAPYEASKIAQQQPSHPVLQLAPP<br>QPAQVAAAAPVAQLSSPPQNLPLPTAHQQSQLPAPVPAPTAPLVTPGALS<br>YSAASAQAVPAPSPASASVKPLKPEPPRPVQQQLDEWTSKYAEYLTRHKT<br>NLASISLRPRGLTNRSNYCYINSILQALLGCSPFYNLLRSIPKQAAVLSE<br>VKTPTVNAMMSFMTNFSSLPSGLRLRLNNLNKGSQSKGKDDFVGSDLQCD<br>MAFEPTEIYKLWNDSREEHVEGRQEDAEEFLGYVLNKLNDEMLEVIKLID<br>KPTPQQNGQEPAEPEDGGDVWQMICNNRNKGSVTRQTDFGRTPVSDIFRG<br>ELRSRLQREGEHSTDVIQPFFTLQLNIEKAASVKEALEILVGRDQLEGVT<br>GSKTKQEVVAWQQMTLEKLPVVLILHLKYFDYRSDGCTKILKKVDFPVEL<br>KIDAKILGSKKTSQKQRAYRLFAVVYHDGKEASKGHYITDVFHTGYSSWL<br>RYDDSSVKPVSEKHVLQPHTPRVPYLLYYRRSDTLPPQQQQTQQQNGGGS<br>GGVVGSSSSSSNAGDNK
**CG4166**	MSETGCRHYQSYVKEHSYDTFRVIDAYFAACVNRDARERKAIHCNCFECG<br>SYGIQLYACLHCIYFGCRGAHITSHLRSKKHNVALELSHGTLYCYACRDF<br>IYDARSREYALINRKLEAKDLQKSIGWVPWVPTTKETNLLLANARRRLVR<br>PNQTIGLRGLLNLGATCFMNCIVQALVHTPLLSDYFMSDRHDCGSKSSHK<br>CLVCEVSRLFQEFYSGSRSPLSLHRLLHLIWNHAKHLAGYEQQDAHEFFI<br>ATLDVLHRHCVKAKAEHESKSNSSGSGSGTNSSNSSSSHCYGQCNCIIDQ<br>IFTGMLQSDVVCQACNGVSTTYDPFWDISLDLGETTTHGGVTPKTLIDCL<br>ERYTRAEHLGSAAKIKCSTCKSYQESTKQFSLRTLPSVVSFHLKRFEHSA<br>LIDRKISSFIQFPVEFDMTPFMSEKKNAYGDFRFSLYAVVNHVGTIDTGH<br>YTAYVRHQKDTWVKCDDHVITMASLKQVLDSEGYLLFYHKNVLEYE

**CG8232**	MDYVYCGTDPIGASEDILSVYDAGSAPGNGHFSPSFNGFNIGTTDPEYVE<br>LVPVLADGGEHFGVSSVAFDDYEELLWMGNQGGHVTSYYTNSMQKYTSFQ<br>VHATDIVRDISTLDSGVLALTQTSLRHQIRRGLPKFTFKSNNMKEMVSML<br>QLSPHRLVMAGLQDELIDFDLRTLKETRIEHVGAGGCTVLRKNSRYLFAG<br>DQLGTVTLRDLNSLSVQHTIKTHTNILSDFSVQGNLLISCGYSGRQNNLA<br>IDRFLMVYDLRMLRLIAPIQVMIDPQMLKFLPSLTSQLAVVSSYGQVQLV<br>DTVELSEPRVSMYQINTNGSQCLSFDISSSSQAMAFGDQSGHINMIAAVQ<br>TPQPQFNLYSRSTEFADVVPQLPMVSITDTNFPLSSVMLPHLTTGTQWFS<br>DWPEELLRYRYHRPKTIDPEVLSNMKMQGPIGYSPNPRTARRNQIPYVIE<br>QGGVCSPNGNGTAAATKAENGVKIIPRRYRKVELKYTKLGTQDFDFDQHN<br>QTCFAGLEATLPNSYCNAMLQILYFTDALRVKLLEHSCIKEFCLSCELGF<br>LFNMLDKSTASSPCQASNFLRSFRTVPEASALGLILTDRSSNVNLISLIQ<br>NWNRFILHQMHYEIFDSSKNASTYSGSVQTSTNAENAGSSETSGSSDLYD<br>SISDENSKEDDRERSKINAETDISKIFGTKQICINRCIKCQEEKIKESIL<br>LACNLSYPNHIKDSDQYFNFGTILKRSLSSEKSIQAFCERCKKFSPTNQS<br>VKVTSLPQILSINCGLNNEKDITFLKRQLNRCSEKTTVDAAASLSTSKPC<br>RYGANCSRSDCHFMHPDRKSPSHTSQPNAVNNSPNGRQKSWFPLTFTMGI<br>NDQGEVQVQTQSDASSGKSEQEEETEKPPTKGLDNNRMYALHAVVCQVDD<br>GTQKNLVSLINVQRPYHTMKLAESADDPQSQWYIFNDFSISPVSPQESVW<br>FTLDWKVPCILFYRHVEDDSESASTTSSTVTESEETIPSESSSGSPTNLS<br>NPFLEEIVSPMLGNLSADATLQPLQSDEMPQSGDLVAMDAEFVTLNPEEN<br>EIRPDGKTATIKPCHMSVARISCIRGQGPAEGVPFMDDYISTQEKVVDYL<br>TQFSGIKPGDLDANFSKKRLTALKYSYQKLKYLVDVGVIFVGHGLKNDFR<br>VINIYVPSEQIIDTVHLFHMPHHRMVSLRFLAWHFLGTKIQSETHDSIED<br>ARTTLQLYKHYLKLQEEKKFANALKNLYERGKQLQWKVPED

**CG2904**	MIKFRYKRKEPTNVVGVAAPTAGAAALGSGASSTPGTSPTSPVSVPVPVQ<br>AHHPHPQLGHLISNNGGTLPRSSPRKLILDGADKSATLTRQQSKQKSVSA<br>LAKVASSVELAVMGYNSTGNGNSNGHINSAGNSERDRCINAVIELFQQLQ<br>TSSEPALCPEPLRRALASGPLAGRRFPLGCLGDAAECFELLLHRVHSHIS<br>PDDGDSCESSACIAHRRFAMRVIEQSVCKCGANSEQLPFTQMVHYVSASA<br>LTSQKSLALQSHQQLSFGQLLRAAGNMGDIRDCPNTCGAKIGICRALLNR<br>PEVVSIGIVWDSERPAADQVHAVLKAVGTSLRLGDVFHQVSEPRWAQQTQ<br>HELVGIVSYYGKHYTTFFFHTKLKVWVYFDDANVKEVGPSWEGVVDKCSR<br>GRYQPLLLLYAVPQQPTVGNGVGIGHVDVAMPASAPSIAGSVVRRAVTPS<br>PEKPSLGNTRRAITPTPLRTPPNDYQNLSVIQKNIFPSNLAMGTDETDAY<br>ISRKTVEHVLSAQYQNLSVIQDKIGGGNGVGVGQQSVDKDGGFLSRKPIE<br>AVLSAQLARRQHLQLQRSHSAESSSGHASSNGSSPPSDGLTMPEHLNQPR<br>RRDSGNWSGDRNSASSASSTTLDNPYLYMVAKRGVAAVPQSPTRHGLPYD<br>PGYDSFSLSSTDSYPPKHALNPQLAKIPEAAMGVVPNGGAMPHGHGNGQL<br>ALSGDCEKLCHEADQLLEKSRLVEESHDLETALVLCNAAAGRARAAMDAP<br>YSNPHTMTFARMKHNTCVMRARSLHRRILVEKGAETEAMPELKHMREGST<br>SSVKHVRQNSKDRTLEKEQQQLQFQQIQQIQQIHQIQQQIPASIEIYATL<br>PKRKSPLKALASASACDDNAIEYEQEKQLATSPGKPERESRSLFGRKDKE<br>KEKRSRSEDRNKLTREFSLTETLLVNAKDTLKKHKEDKDEKKEKDKSGKK<br>QHKIRRKLLMGGLIRRKNRSMPDLTEAVDDSTANAGITHHPHYPAQGVTT<br>AGTLLGTSVDDSAVGLGKHGHGMSGYISEGHFDYSGSGSASNSGAGSNPN<br>PNLERSKLMRKSFHGSGRQLTMVPKVAPPPPMRTSSTLTPQQQQQQQQQF<br>HHEANLSNLSAMSSNTSISEDSCQTIITTCAQVHSEQSPLKDLQMLVAQD<br>ELPLPPPMELPPYPSPPHSSCHSRQASEDFPPPPPPELDLEPLNQQLSQL<br>QALEAASKQQRQQLESLEGTTSILAQLQQRQHLLKLRKEQANHPGAATTD<br>ATWLKELQAKQANDLRAMQRKLEASSPSSVRDLTHRFEQTSIRSYASQEL<br>LSQPGQSQQLGQLRTQMNGLPNGNAKMDVDEVDAMPAVRNSLPAALSAHQ<br>LLPKPKYEMTQSQIAEEIREVELLNTMVQQTLNQNGAAALPKRVKKKSVS<br>FCDQVILVATAGNEEDDDFIPNPILERVLRTAQHPNEKVTAQMIQQQQQN<br>MMRLQTEAQPRQQQVQQLQQPSPMLGRPAGATADMQRYVQRMQQQQQQQQ<br>QQQQQQQQQQQQQQQQQQQQQQQQQQHQELYRQQQLHRTSMSGIASQQHL<br>QQLDAQYTTMPRPLHHSQLGSYSVTLQKHQQLQQQQQQQQQQQAPQQLSL<br>GYFSNGSVATSEQVPLPSPYQRVPLPHGYQPAAAGPYYPLPNAQQHLQQQ<br>QLMAKPVQKKVSFEPGTKGEADCLPPPPPPPQKNGLPETGSAGSGHPGAA<br>PGASGAGGTTSAGITAIPTRVYNNAIVKASAKAVECNLCRKRHVIAPAVY<br>CTNCEYYLQMLNQRR

**CG12743**	MDMQVQRPITSGSRQAPDPYDQYLESRGLYRKHTARDASSLFRVIAEQMY<br>DTQMLHYEIRLECVRFMTLKRRIFEKEIPGDFDSYMQDMSKPKTYGTMTE<br>LRAMSCLYRRNVILYEPYNMGTSVVFNRRYAENFRVFFNNENHFDSVYDV<br>EYIERAAICQSIAFKLLYQKLFKLPDVSFAVEIMLHPHTFNWDRFNVEFD<br>DKGYMVRIHCTDGRVFKLDLPGDTNCILENYKLCNFHSTNGNQSINARKG<br>GRLEIKNQEERKASGSSGHEPNDLLPMCPNRLESCVRQLLDDGISPFPYK<br>VAKSMDPYMYRNIEFDCWNDMRKEAKLYNVYINDYNFKVGAKCKVELPNE<br>TEMYTCHVQNISKDKNYCHVFVERIGKEIVVPYESLHPLPPDEYRPWSLP<br>FRYHRQMPRLPLPKYAGKANKSSKWKKNKLFEMDQYFEHSKCDLMPYMPV<br>DNCYQGVHIQDDEQRDHNDPEQNDQNPTTEQRDREEPQAQKQHQRTKASR<br>VQPQNSSSSQNQEVSGSAAPPPTQYMNYVPMIPSRPGHLPPPWPASPMAI<br>AEEFPFPISGTPHPPPTEGCVYMPFGGYGPPPPGAVALSGPHPFMPLPSP<br>PLNVTGIGEPRRSLHPNGEDLPVDMVTLRYFYNMGVDLHWRMSHHTPPDE<br>LGMFGYHQQNNTDQQAGRTVVIGATEDNLTAVESTPPPSPEVANATEQSP<br>LEKSAYAKRNLNSVKVRGKRPEQLQDIKDSLGPAAFLPTPTPSPSSNGSQ<br>FSFYTTPSPHHHLITPPRLLQPPPPPPIFYHKAGPPQLGGAAQGQTPYAW<br>GMPAPVVSPYEVINNYNMDPSAQPQQQQPATLQPAPLSVQSQPAAVYAAT<br>RHH
**CG14884**	MDSDAAQKTWELENNIQTLPSCDEIFRYDAEQQRQIIDAKPWEKDPHFFK<br>DIKISALALLKMVMHARSGGTLEVMGLMLGKVEDNTMIVMDAFALPVEGT<br>ETRVNAQAQAYEYMTAYMEAAKEVGRMEHAVGWYHSHPGYGCWLSGIDVS<br>TQMLNQTYQEPFVAIVVDPVRTVSAGKVCLGAFRTYPKGYKPPNEEPSEY<br>QTIPLNKIEDFGVHCKQYYPLEISYFKSALDRRLLDSLWNKYWVNTLGSS<br>GLLTNTEYTTGQIMDLSEKLEQSENFLGRGTDVNEKRSEDKLSKATRDCS<br>RSTIELIHGLMAQIVKDKLFNKVGLGK
**CG9124**	MANRANRHAARTEDSDNTINYVQCDGLAVMKMVKHCHEESSNMDLAQGAL<br>LGLVVDKCLEITNCFPFPKSGDETMDEEMYQLTVMRRLRRVNVDHLHVGW<br>YQSSDVGNSLSMALLESQYHYQTSIEESVVVVYDTQKSSRGFLCLKAYRL<br>TPQAIQMYKDGDFTPEAFRTLKVGYENLFAEIPIVIKNSPLTNIMMSELN<br>ELLPEDKGHNFLDLGTATVLENQMRSLIERVDELYQEAVRYNKYQQVVFK<br>QDTEKHRALAKLAAENAVRTSKGEPTVPEEEVIKQFRPMTAPNRLTATIT<br>SGQINTHAQHIAQFCSQSLAKLFITESLQIAKEAKETK

**CG8877**	MSIPPYMIPQNAWAAQLMAQQAYAAAHAQQAQLHAQQQMANQIQQIPPPG<br>APLPPAGGHTNGIPIPVGGQGPGLGQIPTPKPDILTEEKLQEKALKWQHL<br>QSKRFAEKRKFGFVDTQKEDMPPEHIRKIIRDHGDMTSRKYRHDKRVYLG<br>ALKYMPHAVLKLLENMPMPWEQIRDVQVLYHITGAITFVNEIPWVIEPVY<br>IAQWGTMWIMMRREKRDRRHFKRMRFPPFDDEEPPLDYADNVLDVEPLEA<br>IQIELDNDEDNAVYKWFYDHRPLVDTQFVNGTTYRKWNLSLPQLATLYRL<br>ANQLLTDLVDNNFFYLFDPKSFFTAKALNMAIPGGPKFEPLIKDHNVGDE<br>DWNEFNDINKVIIRQPIRTEYRIAFPYLYNNMPHFVHLSWYHTPNVVYIK<br>TEDPDLPAFYFDPLINPISHRNANSKIQEPLPDDDEDFTLPDDVQPFLQD<br>TPLYTDNTANGIALLWAPRPFNMRSGRSRRAIDVPLVKCWYKEHCPPGHP<br>VKVRVSYQKLLKYYVLNALKHRKPKPQKKRYLFRSFKATKFFQTTTLDWV<br>EAGLQVCRQGYNMLNLLIHRKNLNYLHLDYNFNLKPVKTLTTKERKKSRF<br>GNAFHLCREILRLTKLIIDSHVQYRLNNVDAFQLADGLQYIFAHVGQLTG<br>MYRYKYKLMRQIRMCKDLKHLIYYRFNTGPVGKGPGCGFWAPGWRVWLFF<br>MRGITPLLERWLGNLLSRQFEGRHSKGVAKTVTKQRVESHFDLELRASVM<br>HDIVDMMPEGIKQNKARTILQHLSEAWRCWKANIPWKVPGLPIPIENMIL<br>RYVKMKADWWTNTAHYNRERIRRGATVDKTVCKKNLGRLTRLYLKAEQER<br>QHNYLKDGPYISPEEAVAIYTTTVHWLESRRFAPIPFPPLSYKHDTKLLI<br>LALERLKEAYSVKSRLNQSQREELGLIEQAYDNPHEALSRIKRHLLTQRA<br>FKEVGIEFMDLYSHLIPVYEVEPLEKITDAYLDQYLWYEADKRRLFPPWI<br>KPSDTEPPPLLAYKWCQGINNLQDVWDVGEGECNVLLESRFEKLYEKIDL<br>TLLNRLLRLIVDHNIADYMTAKNNVVINYKDMNHTNSYGIIRGLQFSSFI<br>TQYYGLVLDLLVLGLHRSSEMAGPPQMPNDFLTFQDTVTETAHPIRLYCR<br>YVDRIHLFFRFSAEEARDLIQRYLTEHPDPNNENIVGYNNKKCWPRDARM<br>RLMKHDVNLGRAVFWDIKNRLPRSVTTIGWESTFVSVYSKDNPNLLFNMS<br>GFECRILPKCRTQNEEFTHRDGVWNLQNEITKERTAQCFLRVDDESLGRF<br>HNRVRQILMASGSTTFTKIVNKWNTALIGLMTYFREAVVNTQELLDLLVK<br>CENKIQTRIKIGLNSKMPSRFPPVVFYTPKELGGLGMLSMGHVLIPQSDL<br>RWSKQTDVGITHFRSGMSHDEDQLIPNLYRYIQPWESEFIDSQRVWAEYA<br>LKRQEANAQNRRLTLEDLEDSWDRGIPRINTLFQKDRHTLAYDKGWRIRT<br>EFKQYQVLKQNPFWWTHQRHDGKLWNLNNYRTDMIQALGGVEGILEHTLF<br>KGTYFPTWEGLFWEKASGFEESMKYKKLTNAQRSGLNQIPNRRFTLWWSP<br>TINRANVYVGFQVQLDLTGIFMHGKIPTLKISLIQIFRAHLWQKIHESIV<br>MDLCQVFDQELDALEIETVQKETIHPRKSYKMNSSCADILLFPAYKWNVS<br>RPSLLADTKDTMDNTTTQKYWLDIQLRWGDYDSHDVERYARAKFLDYTTD<br>NMSIYPSPTGVLIAIDLAYNLHSAYGNWFPGCKTLIQQAMAKIMKANPAL<br>YVLRERIRKALQLYSSEPTEPYLSSQNYGELFSNQIIWFVDDTNVYRVTI<br>HKTFEGNLTTKPINGAIFIFNPRTGQLFLKIIHTSVWAGQKRLGQLAKWK<br>TAEEVAALIRSLPVEEQPKQIIVTRKGMLDPLEVHLLDFPNIVIKGSELQ<br>LPFQACLKVEKFGDLILKATEPQMVLFNLYDDWLKTISSYTAFSRLILIL<br>RALHVNTERTKIILKPDKTTITEAHHIWPTLTDEEWIKVEVQLKDLILAD<br>YGKKNNVNVASLTQSEIRDIILGMEISAPSAQRQQIAEIEKQTKEQNQLT<br>ATTTRTTNKHGDEIITSTTSNYETQTFSSKTEWRVRAISATNLHLRTNHI<br>YVSSDDIKETGYTYILPKNILKKFVTISDLRAQIAGYLYGVSPPDNPQVK<br>EIRCIVMPPQWGTHQTINLPNTLPTHQYLKDMEPLGWIHTQPNELPQLSP<br>QDITTHAKIMQENSNWDGEKTIVITCSFTPGSCSLTAYKLTPSGFEWGSK<br>NTDKGNNPKGYLPSHYERVQMLLSNKFLGFFMVPAQSSWNYNFMGVRHDP<br>NMKYELQLANPKEFYHELHRTSHFLLFSNLEDGGDGAGADREDVYA

CG4751

MENGVEHGVDESGENQVLSSSDGEGDCDGDGEVEGEVLQPPPPPPLQITN
DCVGEGVQAEEGERAPATTGAVDVTPDPGPPPTDGAAPVAILEDNMCDKD
VDSDAGDEDNDDETKENYEGFNGTGRTVTLQTLMAANVLQPGLGLMTIEY
LGQKFVGDLLADGKIKSHETETIFLTPSAWAMHCKRIINPDKKSGCGWAS
VKYKGKKLDAYKNTYLRKCALQKETPLDDCELDAERKTDTPEIVVKRTVF
AHNTVSNRNVVHDANMLIESVPFTSVGKLQPFLITVNSSALLLADFHCHL
TVREVCGYLGGTWDMNTHTLSITKTYPCRSTRFDRQRAGEVERDIQKMMI
QDQLLLVGWYHSHPKFQAEPTLRDCDAQLDYQIKMRGASDLTYTPCVSLI
ISPYYDENPTLESVVKCIWIVPPNENRQSMEYGRPMLMQYSVLPDKEIPE
EVRSEIQLCVDYYSQYRSEMVKFRNIYNNDVTYNEKLKNTLYPKFPSKQS
DKALWNWICAVLDCEQEDDFIPPKTIKIIDNDDLEVKEEDKPVVLMDLSG
DVKINPPKEEQFSEAMGGLEDSGRKAEEESNAQAEQKASELKVMSLQEQL
CMPSGLNMNPVRMLSPLATPNPTSLPPVLPNLGAPVLPATPSQLLPPQVP
AVTAPPAITPAVTTSALTSALNASPRDSPITIQSNSASPAKFEVPVRASP
SPAKSDTSSHASTSRTRNSPAPSPGKFSVSDIARNSPSITPNKYEAAAAA
LVPPAAACLPTANDLMAASLAQLAGQLPPNFLQGDLAALFQQQRKDYGSS
SLNQLAAAAAKVGGSKQSNASASSGLNDPNVAAAVAAYSNSFNMPLPTGV
GIGGSGNSNAHSSSKSKSERSSKSSSSSSSSNSSSTSNSYKTKLMKELDE
LKNDPLKMSELIRSPEYAALLLQQAEALGATTLGTLGFGSDYSYLTGAGL
GVPAANALTGGQSSNSSSSKSSKSSPAAAAAAALSADYNNLIQASKLLGY
DSYMQQSKQSNDLNAFLQQQMAVAAASIPPPPQTASSGSSNSSSSKKQQQ
LQQQQHQQQQQQQQQQAAAQADYTALLQTYTKLFDPNNQFAAAMSSNKHM
AGAHNELSALLSSGVGVGGGASGGGSKQKQKDIQSDMLNQLLQLEKQDSE
IKALLYRQNKAAADLDALFATPSGAVVGAGSSANAMKSGSSAGNSGVSGM
SSPSSLSNQAAYYNALAQEKMQDYAAFFQQQHGKYGIPDPLSKTTLAANN
MFMTPSALFKIQQESLSAMMMKPPKSTTPSSARTRESSASPALERLTPTK
SASSGGSGGGGSNSNSGGKYNFSAVDLAISSVPSNTPSPAPSDGSSGSSH
RRPSPDIGRLYGELAPPGALLGSGGVPKKRMEFASVADLAAPPPAKMPKN
NMGDDILNLSHD
