# Supplementary material for: Systematic Analysis of the Physiological Importance of Deubiquitinating Enzymes
Source: PLoS One. 2012 Aug 24;7(8):e43112. doi: 10.1371/journal.pone.0043112 (PMC3427330; doi:10.1371/journal.pone.0043112)
Supplement: File S2 — Human and fly DUB domain designations and comparisons. (PDF) [file pone.0043112.s005.pdf]

# UCH Domains

**FlyDUB: CG8445 (Calypso)**

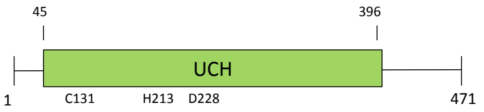


**Closest Human DUB: BAP1**

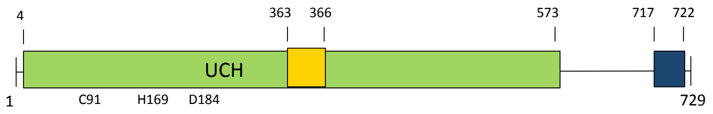


HBM-like motif
Nuclear localization signal

Coverage – 73%
E-value – 2e-107
Max Identity – 61%

**FlyDUB: CG4265 (Uch)**

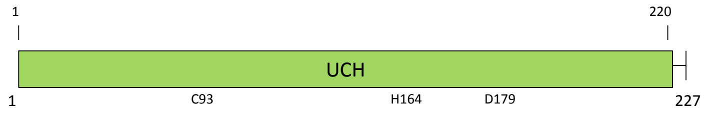


**Closest Human DUB: UCHL3**

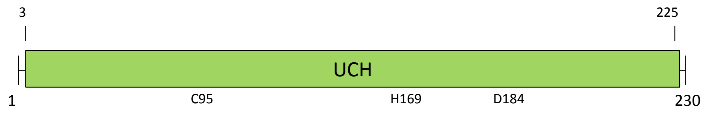


**Second Closest Human DUB: UCHL1**

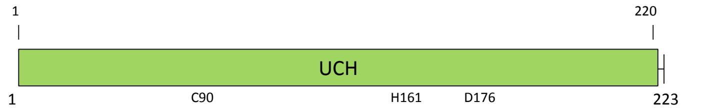


UCHL3:
Coverage – 97%
E-value – 2e-77
Max Identity – 51%

UCHL1:
Coverage – 98%
E-value –1e-66
Max Identity –45%

**FlyDUB: CG3431 (Uch-L3)**

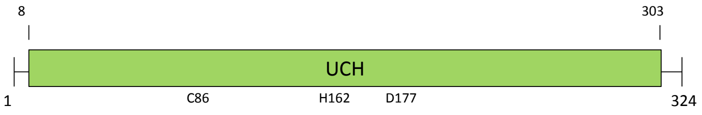


**Closest Human DUB: UCH-L5/UCH37**

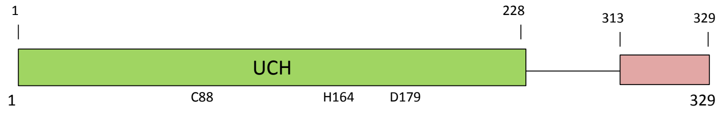


ADRM1 interacting region

Coverage – 99%
E-value – 2e-144
Max Identity – 60%

# USP Domains

**FlyDUB: CG15817**

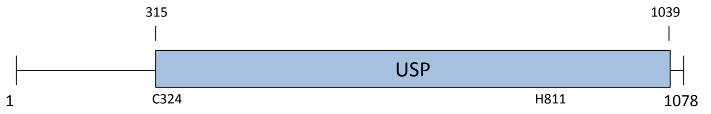


**Closest Human DUB: USP1**

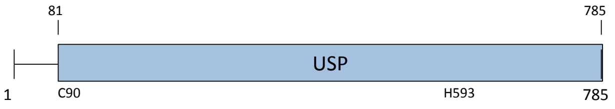


Coverage – 41%

E-value – 4e-18

Max Identity – 41%

**FlyDUB: CG14619**

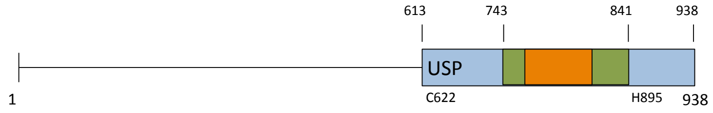


**Closest Human DUB: USP2**

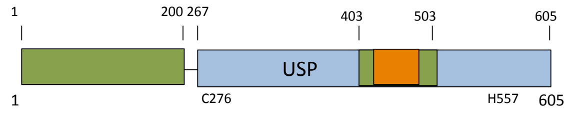


Zinc-binding residues:
425, 428, 476, 479 in USP2
765, 768, 814, 817 in CG14619

MDM4 interacting domain: E-value 2e-33, 56% identity

Coverage – 34%

E-value – 1e-91

Max Identity – 47%

**FlyDUB: CG12082**

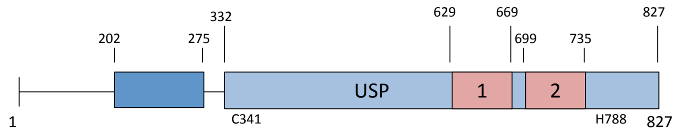


**Closest Human DUB: USP5/isopeptidase T**

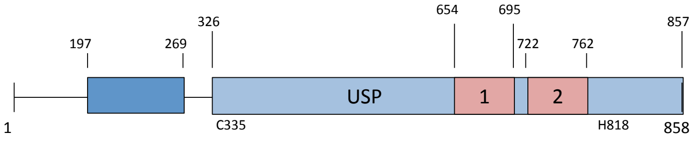


UBA domains:
1 E-value 2e-13, 57% identity
2 E-value 3e-10, 51% identity

UBP-type zinc finger: E-value 3e-35, 77% identity

Coverage – 99%

E-value – 0.0

Max Identity – 51%

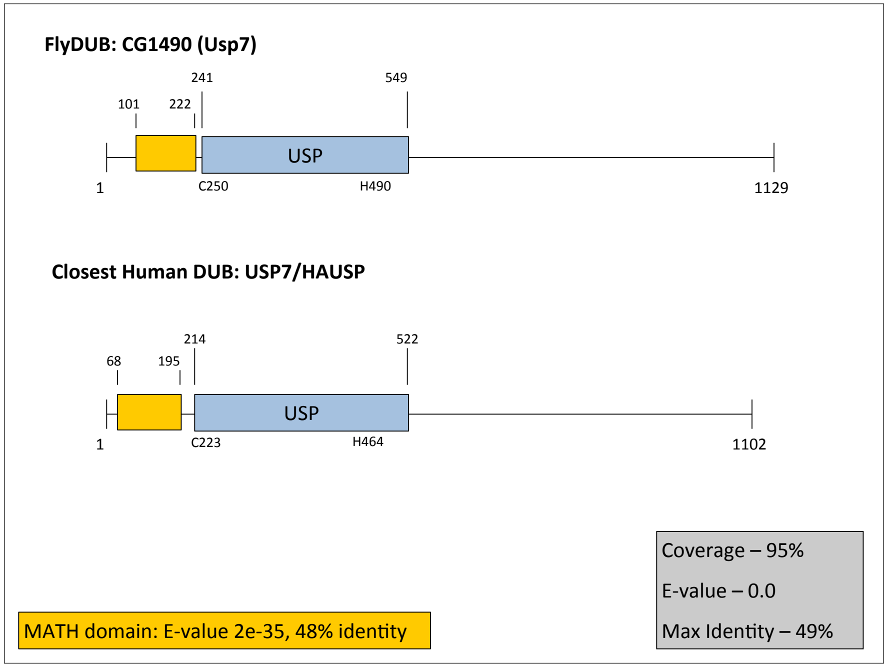
FlyDUB: CG1490 (Usp7)
241
549
101
222
USP
1
C250
H490
1129
Closest Human DUB: USP7/HAUSP
214
522
68
195
USP
1
C223
H464
1102
Coverage – 95%
E-value – 0.0
Max Identity – 49%
MATH domain: E-value 2e-35, 48% identity

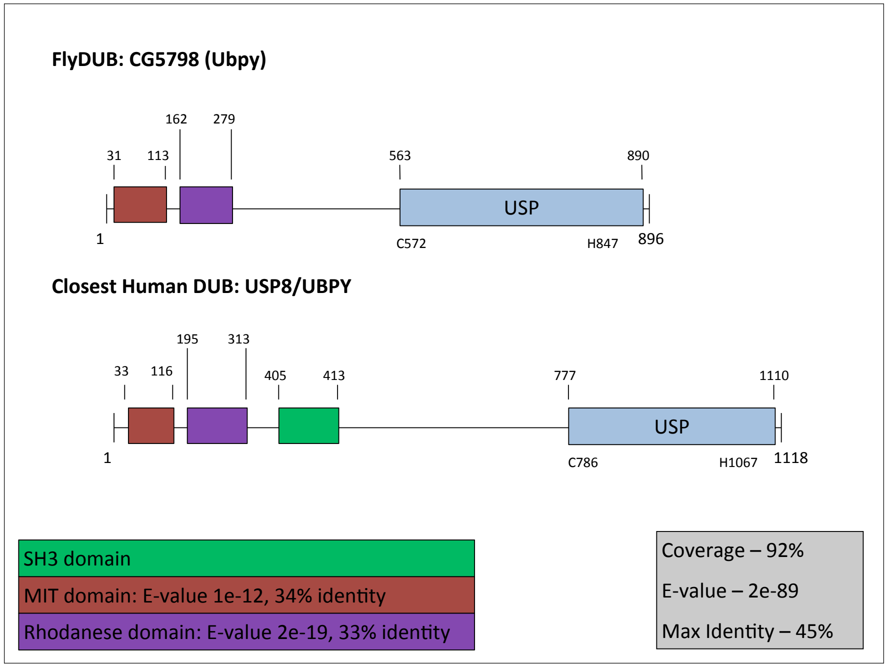
FlyDUB: CG5798 (Ubpy)
162
279
31
113
563
890
USP
1
C572
H847
896
Closest Human DUB: USP8/UBPY
195
313
33
116
405
413
777
1110
USP
1
C786
H1067
1118
SH3 domain
MIT domain: E-value 1e-12, 34% identity
Rhodanese domain: E-value 2e-19, 33% identity
Coverage – 92%
E-value – 2e-89
Max Identity – 45%

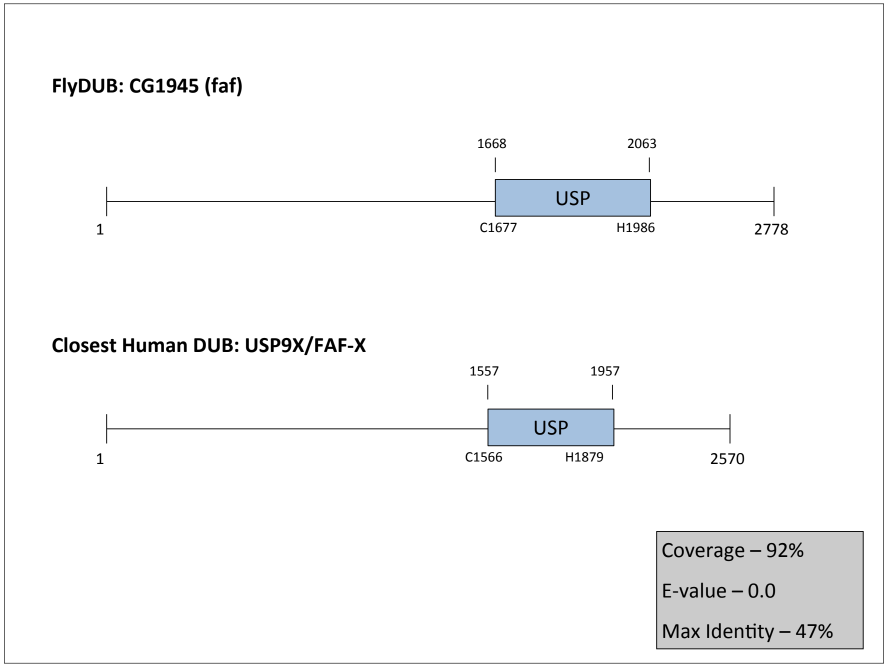
FlyDUB: CG1945 (faf)
1668
2063
USP
1
C1677
H1986
2778
Closest Human DUB: USP9X/FAF-X
1557
1957
USP
1
C1566
H1879
2570
Coverage – 92%
E-value – 0.0
Max Identity – 47%

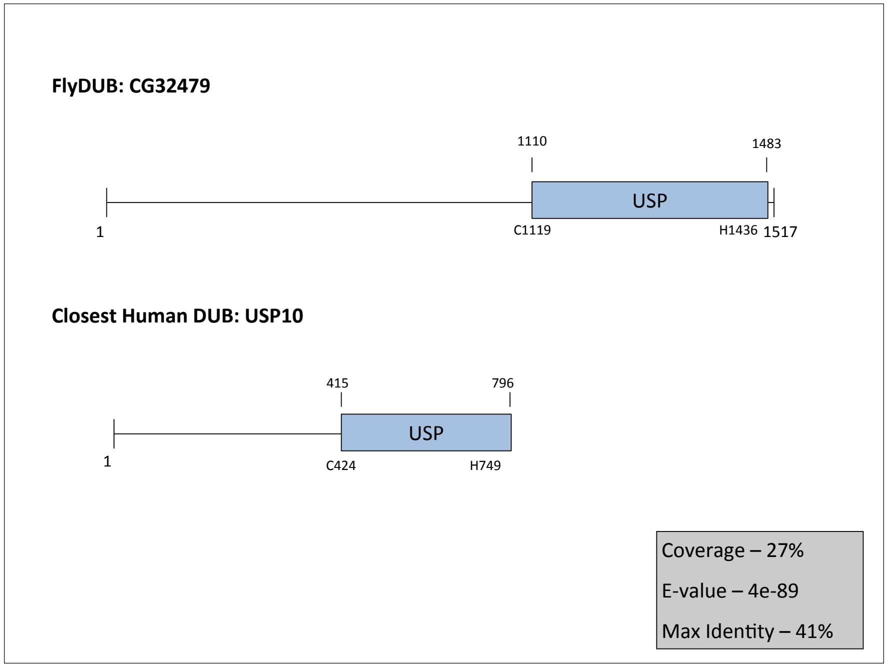
FlyDUB: CG32479
1110
1483
USP
1
C1119
H1436
1517
Closest Human DUB: USP10
415
796
USP
1
C424
H749
Coverage – 27%
E-value – 4e-89
Max Identity – 41%

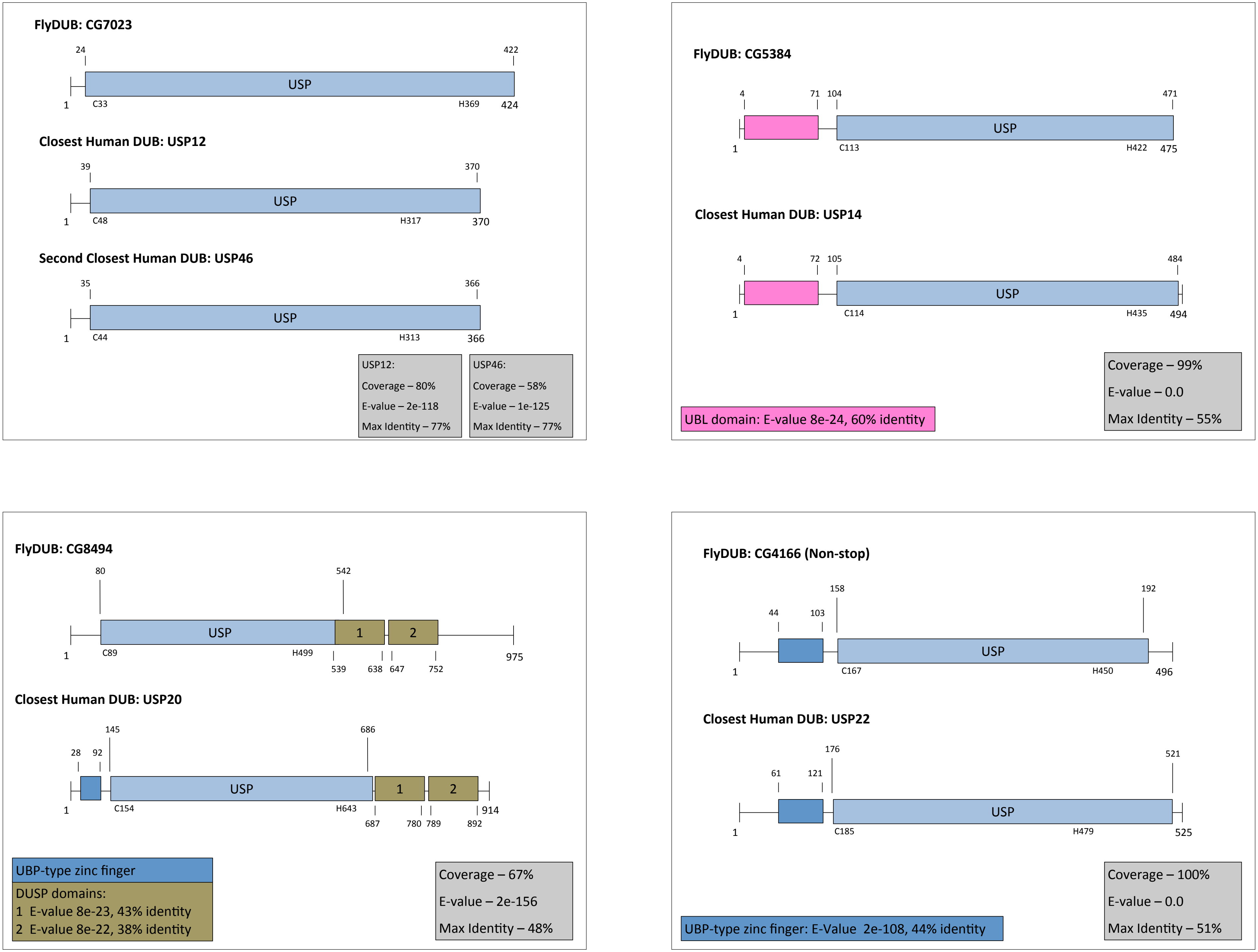
FlyDUB: CG7023
24
422
USP
1
C33
H369
424
Closest Human DUB: USP12
39
370
USP
1
C48
H317
370
Second Closest Human DUB: USP46
35
366
USP
1
C44
H313
366
USP12:
Coverage – 80%
E-value – 2e-118
Max Identity – 77%
USP46:
Coverage – 58%
E-value – 1e-125
Max Identity – 77%
FlyDUB: CG5384
4
71
104
471
USP
1
C113
H422
475
Closest Human DUB: USP14
4
72
105
484
USP
1
C114
H435
494
Coverage – 99%
E-value – 0.0
Max Identity – 55%
UBL domain: E-value 8e-24, 60% identity
FlyDUB: CG8494
80
542
USP
1
2
1
C89
H499
975
539
638
647
752
Closest Human DUB: USP20
145
686
28
92
USP
1
2
1
C154
H643
914
687
780
789
892
UBP-type zinc finger
DUSP domains:
1 E-value 8e-23, 43% identity
2 E-value 8e-22, 38% identity
Coverage – 67%
E-value – 2e-156
Max Identity – 48%
FlyDUB: CG4166 (Non-stop)
158
192
44
103
USP
1
C167
H450
496
Closest Human DUB: USP22
176
521
61
121
USP
1
C185
H479
525
Coverage – 100%
E-value – 0.0
Max Identity – 51%
UBP-type zinc finger: E-Value 2e-108, 44% identity

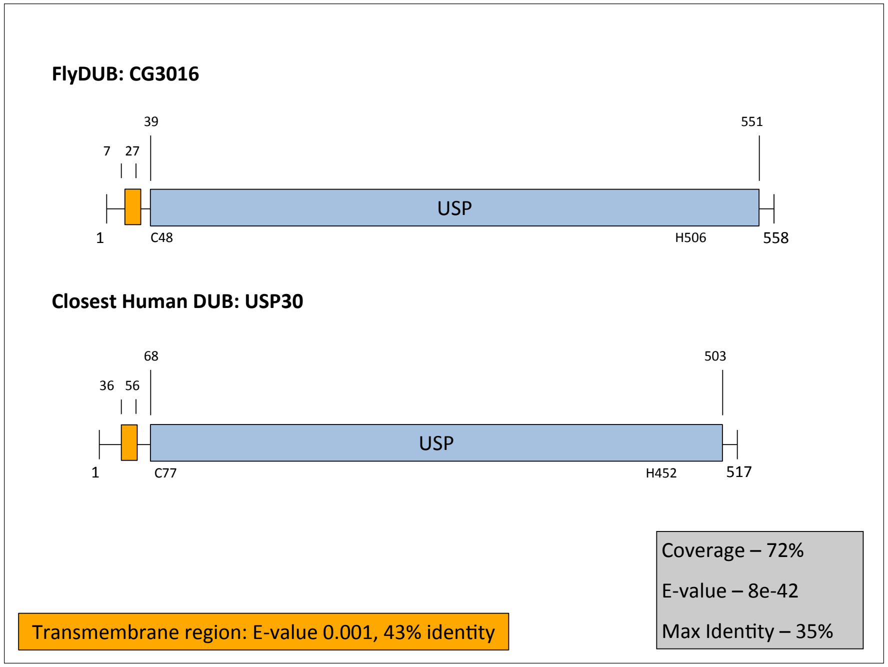
FlyDUB: CG3016
7
27
39
551
USP
1
C48
H506
558
Closest Human DUB: USP30
36
56
68
503
USP
1
C77
H452
517
Coverage – 72%
E-value – 8e-42
Max Identity – 35%
Transmembrane region: E-value 0.001, 43% identity

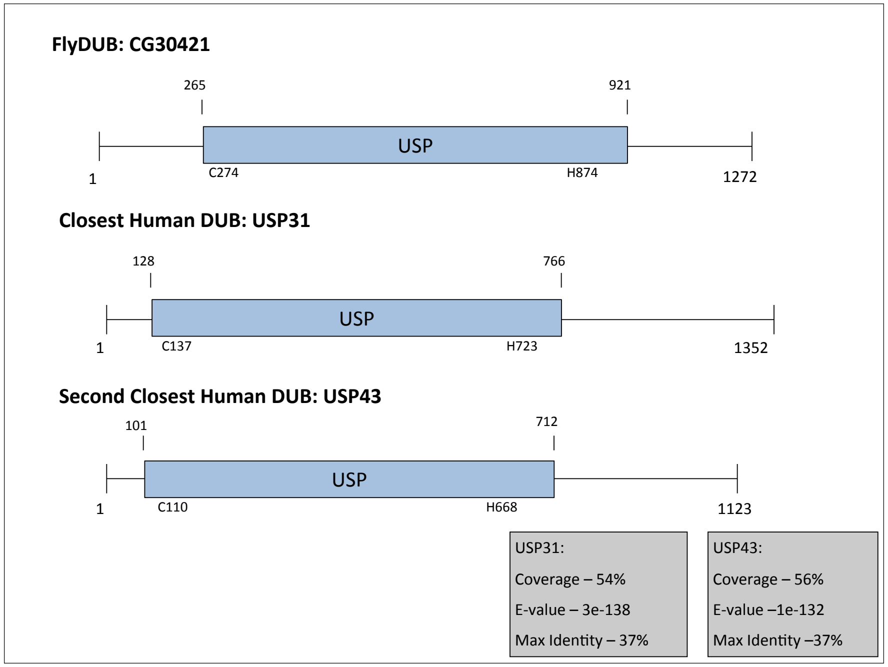
FlyDUB: CG30421
265
921
USP
1
C274
H874
1272
Closest Human DUB: USP31
128
766
USP
1
C137
H723
1352
Second Closest Human DUB: USP43
101
712
USP
1
C110
H668
1123
USP31:
Coverage – 54%
E-value – 3e-138
Max Identity – 37%
USP43:
Coverage – 56%
E-value –1e-132
Max Identity –37%

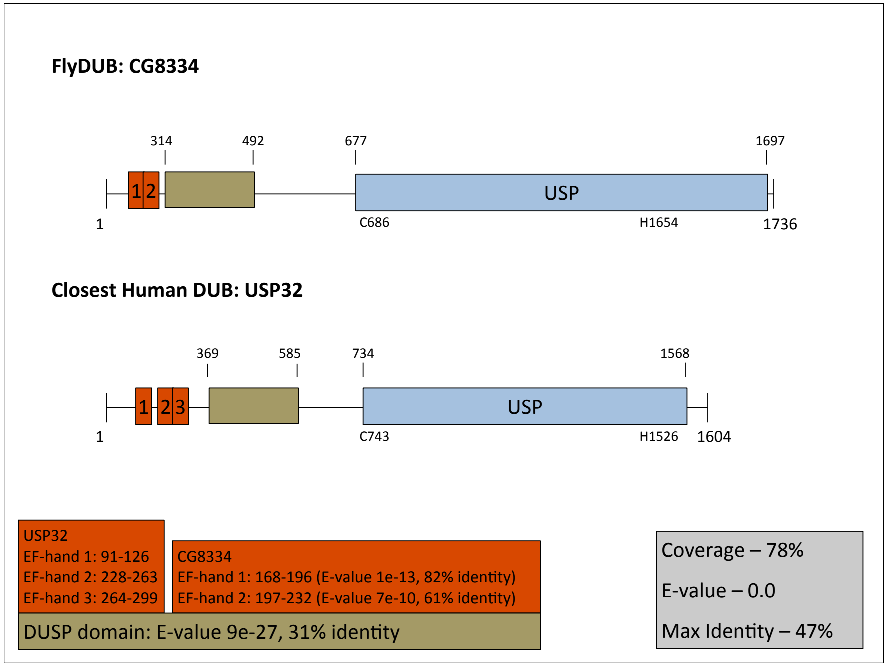
FlyDUB: CG8334
314
492
677
1697
1 2
USP
1
C686
H1654
1736
Closest Human DUB: USP32
369
585
734
1568
1 2 3
USP
1
C743
H1526
1604
USP32
EF-hand 1: 91-126
EF-hand 2: 228-263
EF-hand 3: 264-299
CG8334
EF-hand 1: 168-196 (E-value 1e-13, 82% identity)
EF-hand 2: 197-232 (E-value 7e-10, 61% identity)
DUSP domain: E-value 9e-27, 31% identity
Coverage – 78%
E-value – 0.0
Max Identity – 47%

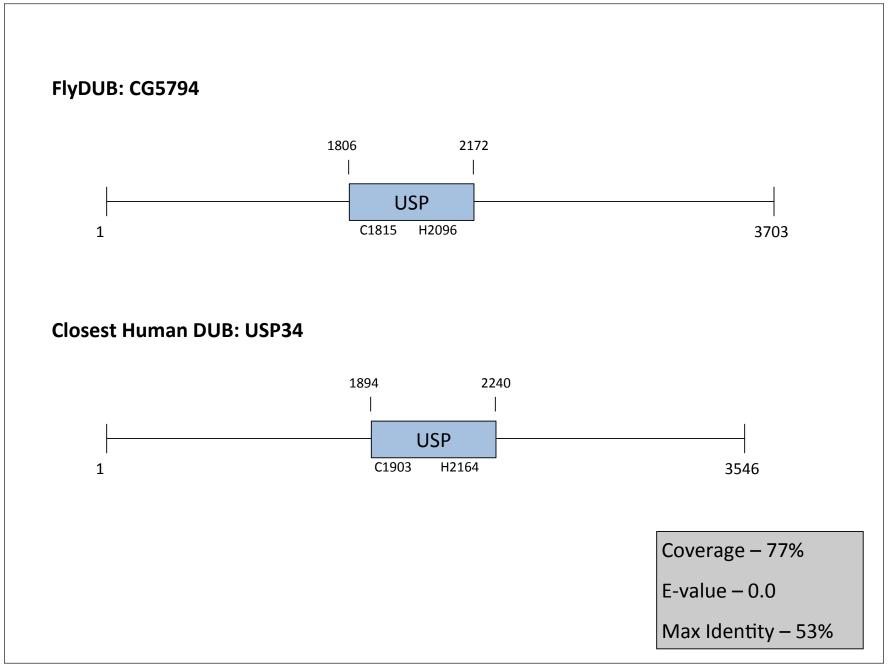
FlyDUB: CG5794
1806
2172
USP
1
C1815
H2096
3703
Closest Human DUB: USP34
1894
2240
USP
1
C1903
H2164
3546
Coverage – 77%
E-value – 0.0
Max Identity – 53%

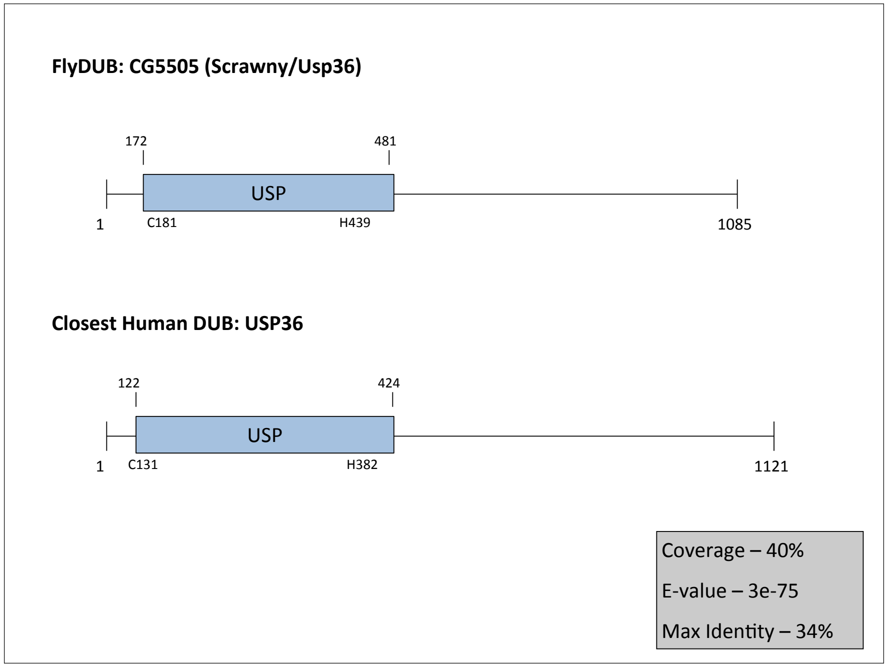
FlyDUB: CG5505 (Scrawny/Usp36)
172
481
USP
1
C181
H439
1085
Closest Human DUB: USP36
122
424
USP
1
C131
H382
1121
Coverage – 40%
E-value – 3e-75
Max Identity – 34%

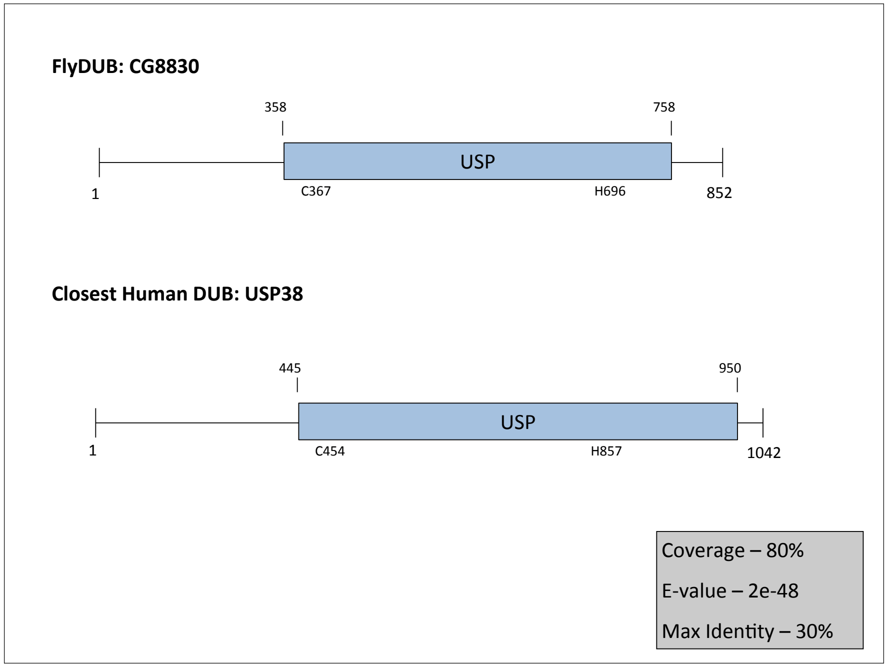
FlyDUB: CG8830
358
758
USP
1
C367
H696
852
Closest Human DUB: USP38
445
950
USP
1
C454
H857
1042
Coverage – 80%
E-value – 2e-48
Max Identity – 30%

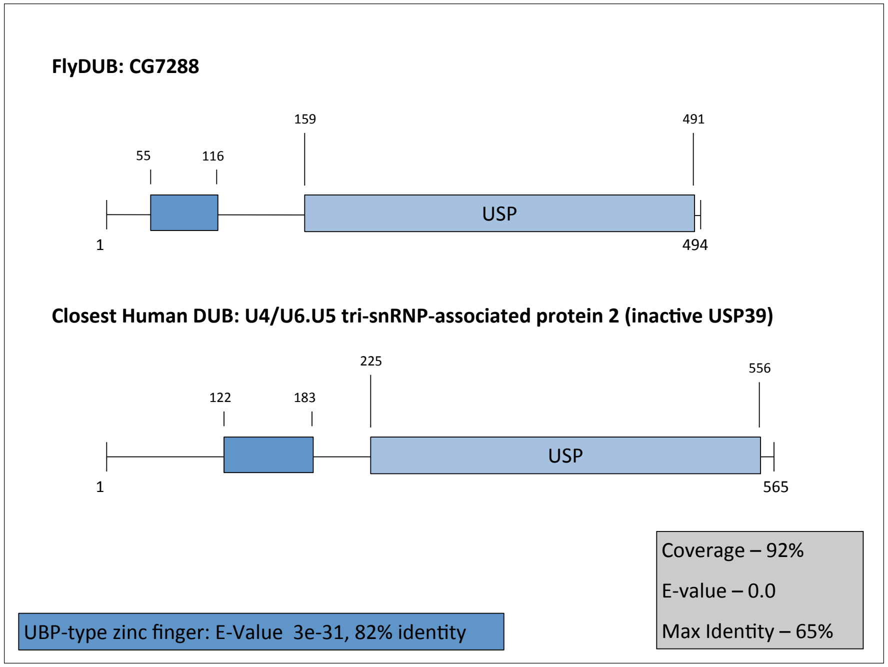
FlyDUB: CG7288
159
491
55
116
USP
1
494
Closest Human DUB: U4/U6.U5 tri-snRNP-associated protein 2 (inactive USP39)
225
556
122
183
USP
1
565
Coverage – 92%
E-value – 0.0
Max Identity – 65%
UBP-type zinc finger: E-Value 3e-31, 82% identity

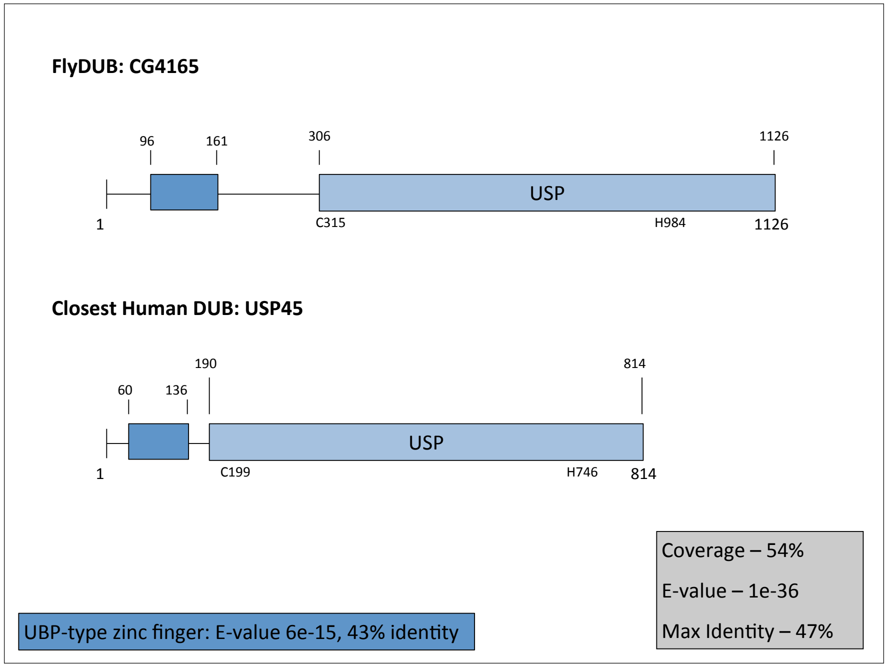
FlyDUB: CG4165
96
161
306
1126
USP
1
C315
H984
1126
Closest Human DUB: USP45
190
814
60
136
USP
1
C199
H746
814
Coverage – 54%
E-value – 1e-36
Max Identity – 47%
UBP-type zinc finger: E-value 6e-15, 43% identity

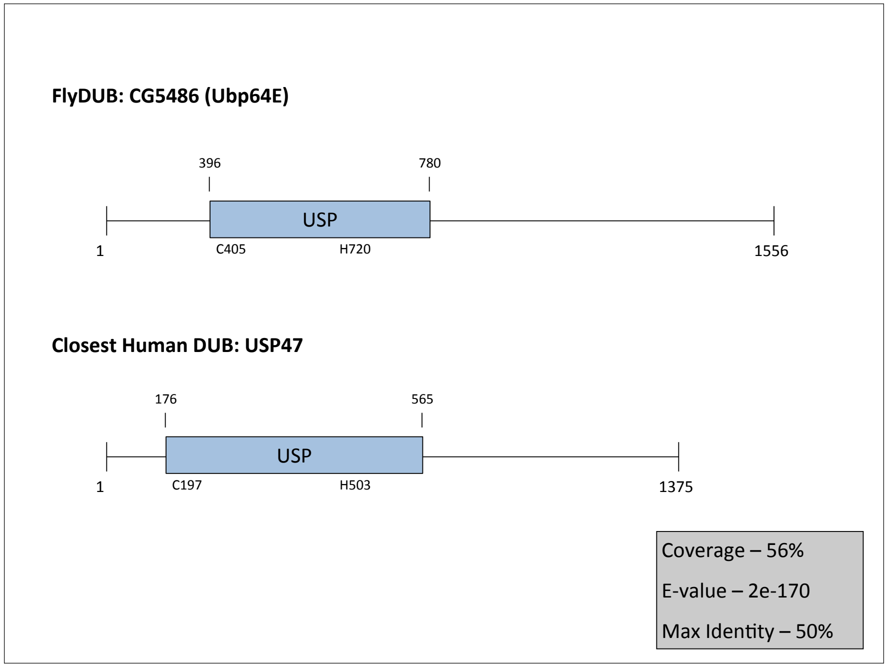
FlyDUB: CG5486 (Ubp64E)
396
780
USP
1
C405
H720
1556
Closest Human DUB: USP47
176
565
USP
1
C197
H503
1375
Coverage – 56%
E-value – 2e-170
Max Identity – 50%

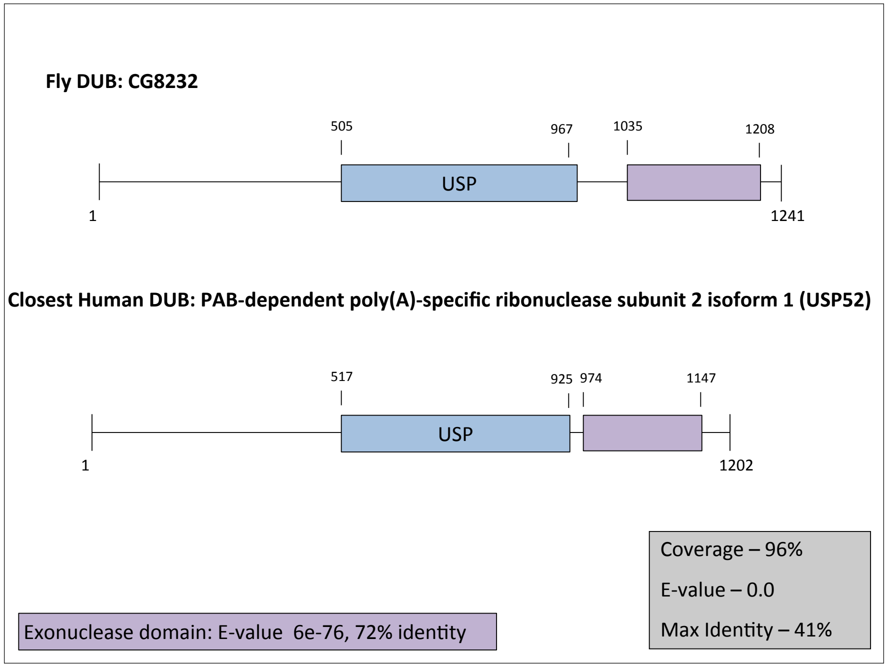
Fly DUB: CG8232
505
967
1035
1208
USP
1
1241
Closest Human DUB: PAB-dependent poly(A)-specific ribonuclease subunit 2 isoform 1 (USP52)
517
925
974
1147
USP
1
1202
Coverage – 96%
E-value – 0.0
Max Identity – 41%
Exonuclease domain: E-value 6e-76, 72% identity

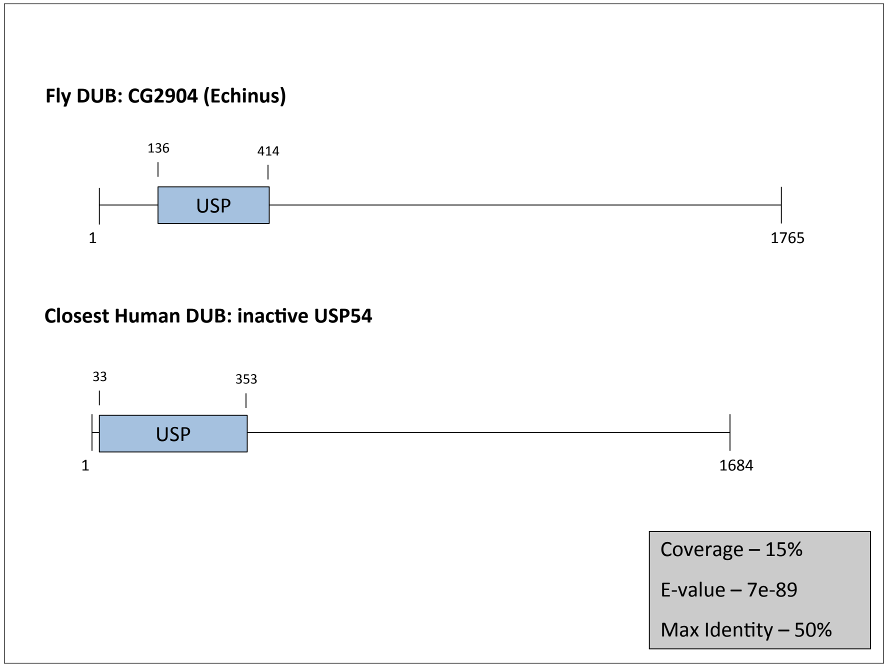
Fly DUB: CG2904 (Echinus)
136
414
USP
1
1765
Closest Human DUB: inactive USP54
33
353
USP
1
1684
Coverage – 15%
E-value – 7e-89
Max Identity – 50%

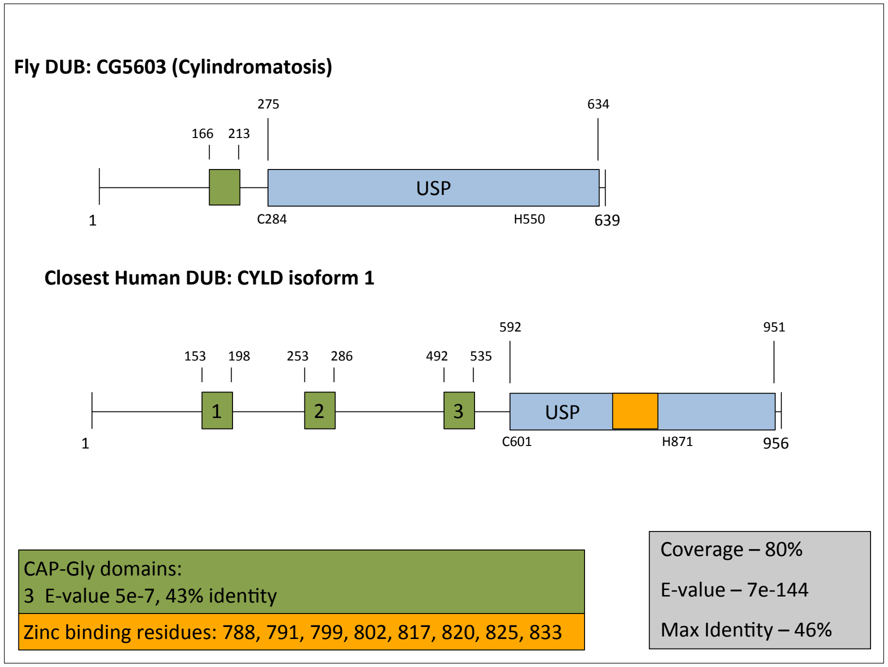
Fly DUB: CG5603 (Cylindromatosis)
275
634
166
213
USP
1
C284
H550
639
Closest Human DUB: CYLD isoform 1
592
951
153
198
253
286
492
535
1
2
3
USP
1
C601
H871
956
CAP-Gly domains:
3 E-value 5e-7, 43% identity
Zinc binding residues: 788, 791, 799, 802, 817, 820, 825, 833
Coverage – 80%
E-value – 7e-144
Max Identity – 46%

# Josephin Domains

**Fly DUB: CG3781**

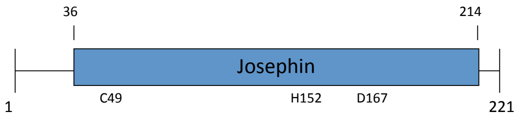


**Closest Human DUB: JOSD2**

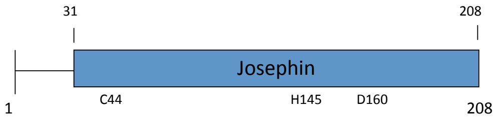


**Second Closest Human DUB: JOSD1**

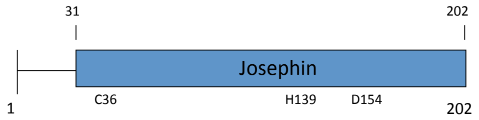


Josephin Domains:
JOSD2 E-value 3e-44, 50% identity
JOSD1 E-value 7e-43, 44% Identity

JOSD2
Coverage – 85%
E-value – 1e-53
Max Identity – 49%

JOSD1
Coverage – 77%
E-value – 1e-48
Max Identity – 45%

# OTU Domains

**Fly DUB: CG12743 (Otu)**

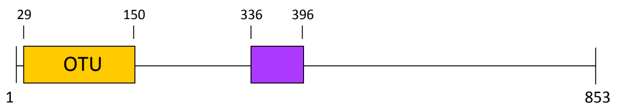


**Closest Human DUB: OTU domain-containing protein 4 (OTUD4), isoform CRA-f**

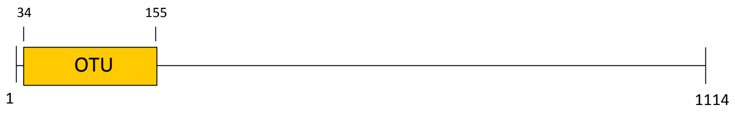


putative Tudor domain by Prosite analysis

Coverage – 56%

E-value – 2e-42

Max Identity – 30%

**Fly DUB: CG9448 (Trabid)**

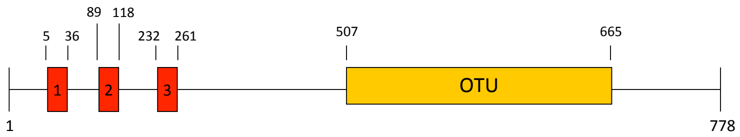


**Closest Human DUB: TRABID**

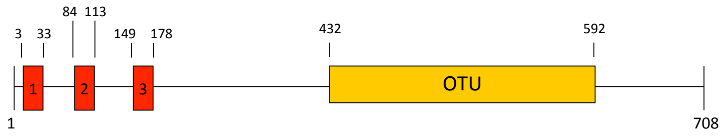


RanBP2-type zinc finger:
1 E-value 5e-12, 67% identity
2 E-value 2e-11, 57% identity
3 E-value 1e-10, 68% identity

OTU Domain: E-value 2e-70, 68% identity

Coverage – 96%

E-value – 0.0

Max Identity – 43%

**Fly DUB: CG4968**

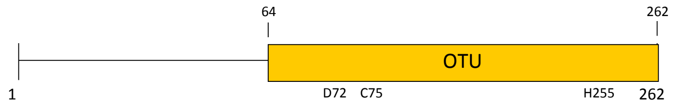


**Closest Human DUB: ubiquitin thioesterase OTUB1**

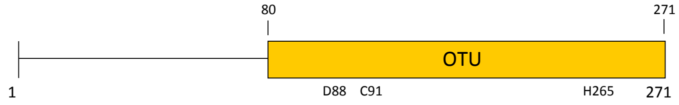


Coverage – 95%

E-value – 8e-98

Max Identity – 56%

**Fly DUB: CG6091**

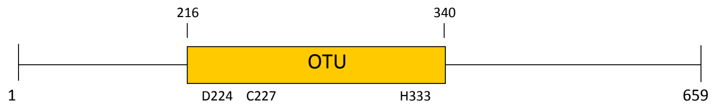


**Closest Human DUB: OTUD5 protein**

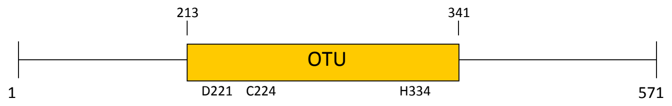


Coverage – 36%

E-value – 1e-76

Max Identity – 48%

**Fly DUB: CG7857**

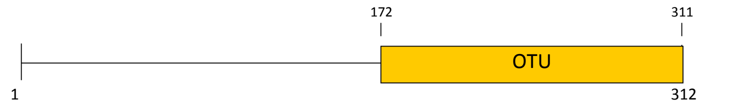


**Closest Human DUB: OTU domain-containing protein 6B, OTU6B**

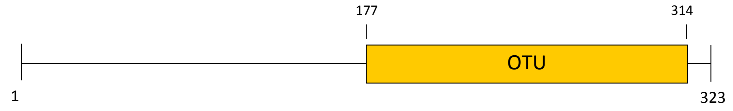


Coverage – 91%

E-value – 8e-61

Max Identity – 39%

**FlyDUB: CG4603**

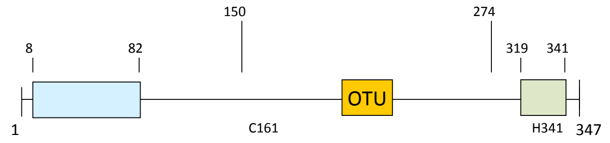


**Closest Human DUB: OTU1/YOD1**

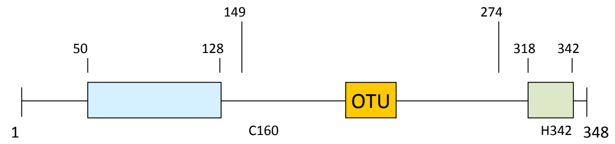


UBX-like domain: E-value 9e-11, 37% identity

C2H2-type zinc finger: E-value 1e-8, 64% identity

Coverage – 98%

E-value – 5e-84

Max Identity – 42%

# MPN Domains

**Fly DUB: CG14884 (Csn5)**

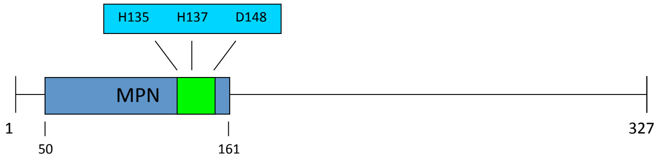


**Closest Human DUB: COP9 signalosome complex subunit 5, SCN5**

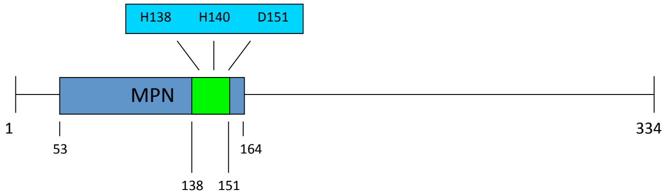


JAMM Motif

Zinc Coordinating Residues

MPN Domain: E-value 1e-55, 83% identity

Coverage – 98%

E-value – 0.0

Max Identity – 75%

**Fly DUB: CG6932 (Csn6)**

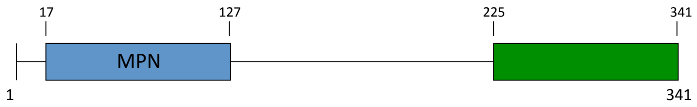


**Closest Human DUB: COP9 signalosome complex subunit 6, CSN6**

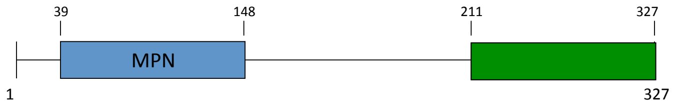


MPN Domain: E-value 2e-30, 60% identity

Interaction with VPR: E-value 2e-46, 64% identity

Coverage – 87%

E-value – 2e-146

Max Identity – 62%

**Fly DUB: CG3416 (Mov34)**

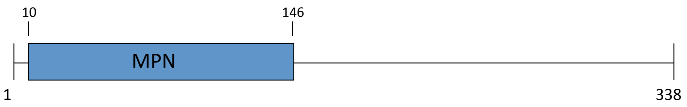


**Closest Human DUB: 26S proteasome non-ATPase regulatory subunit 7, PSMD7**

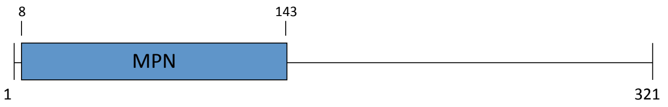


MPN Domain: E-value 6e-75, 90% identity

Coverage – 85%

E-value – 2e-170

Max Identity – 82%

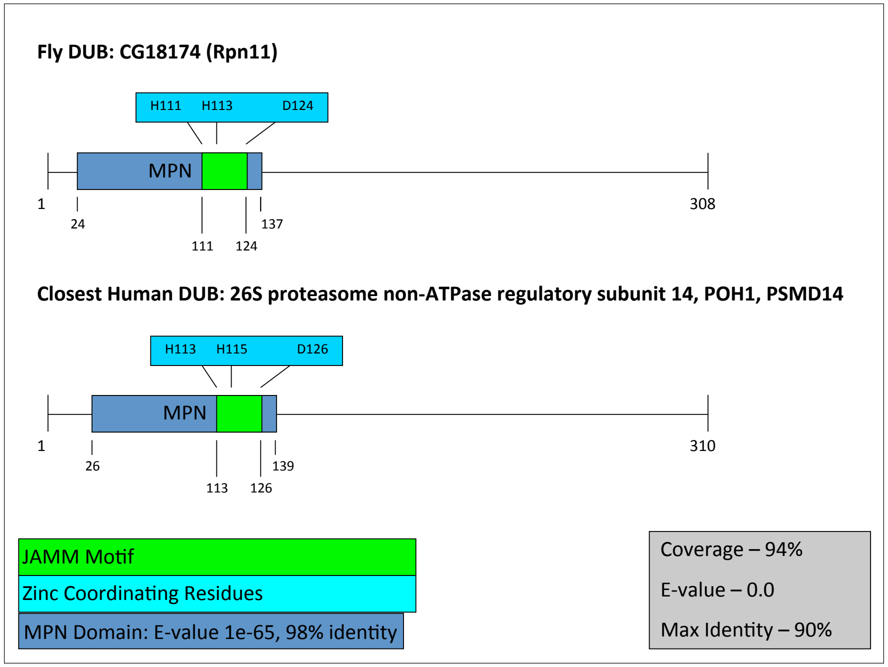
Fly DUB: CG18174 (Rpn11)
H111 H113 D124
MPN
1
24
111 124
137
308
Closest Human DUB: 26S proteasome non-ATPase regulatory subunit 14, POH1, PSMD14
H113 H115 D126
MPN
1
26
113 126
139
310
JAMM Motif
Zinc Coordinating Residues
MPN Domain: E-value 1e-65, 98% identity
Coverage – 94%
E-value – 0.0
Max Identity – 90%

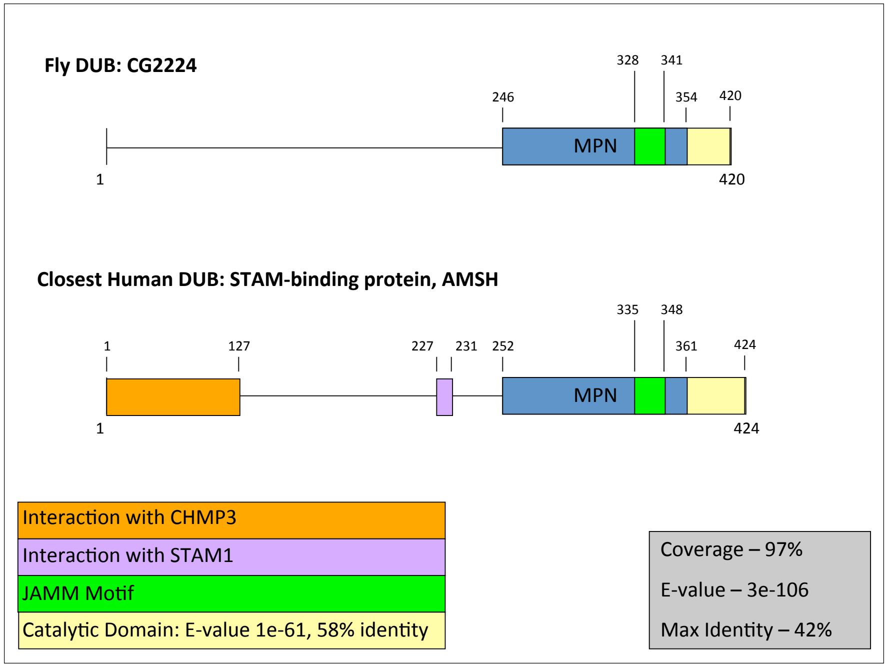
Fly DUB: CG2224
246 328 341 354 420
MPN
1
420
Closest Human DUB: STAM-binding protein, AMSH
1 127 227 231 252 335 348 361 424
MPN
1
424
Interaction with CHMP3
Interaction with STAM1
JAMM Motif
Catalytic Domain: E-value 1e-61, 58% identity
Coverage – 97%
E-value – 3e-106
Max Identity – 42%

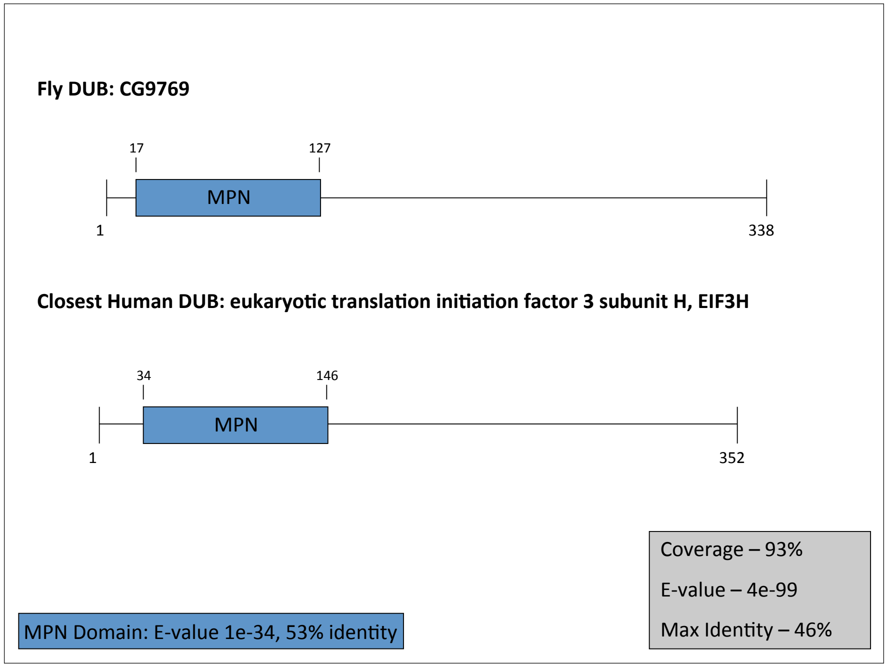
Fly DUB: CG9769
17 127
MPN
1
338
Closest Human DUB: eukaryotic translation initiation factor 3 subunit H, EIF3H
34 146
MPN
1
352
MPN Domain: E-value 1e-34, 53% identity
Coverage – 93%
E-value – 4e-99
Max Identity – 46%

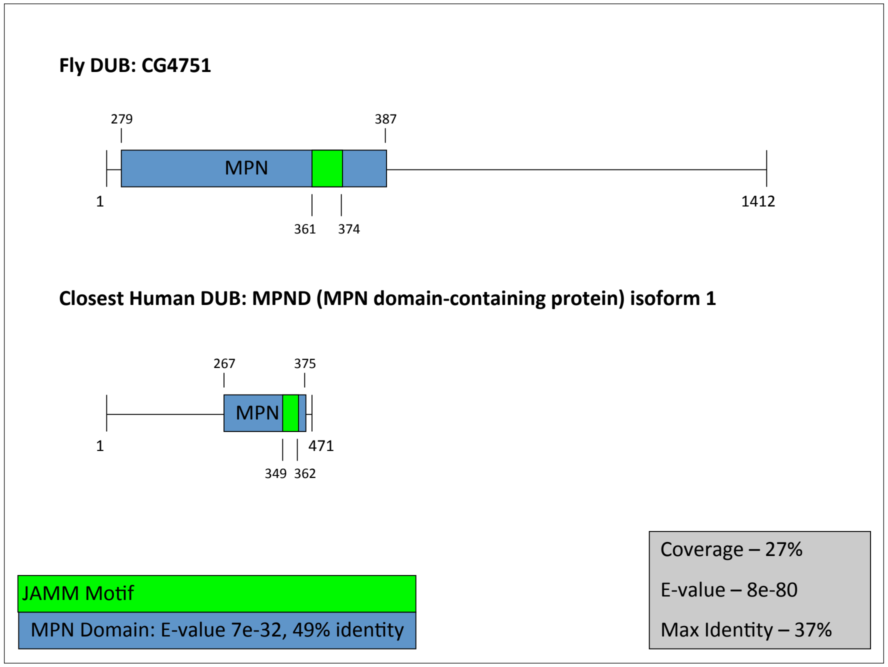
Fly DUB: CG4751
279 387
MPN
1
1412
361 374
Closest Human DUB: MPND (MPN domain-containing protein) isoform 1
267 375
MPN
1
471
349 362
JAMM Motif
MPN Domain: E-value 7e-32, 49% identity
Coverage – 27%
E-value – 8e-80
Max Identity – 37%

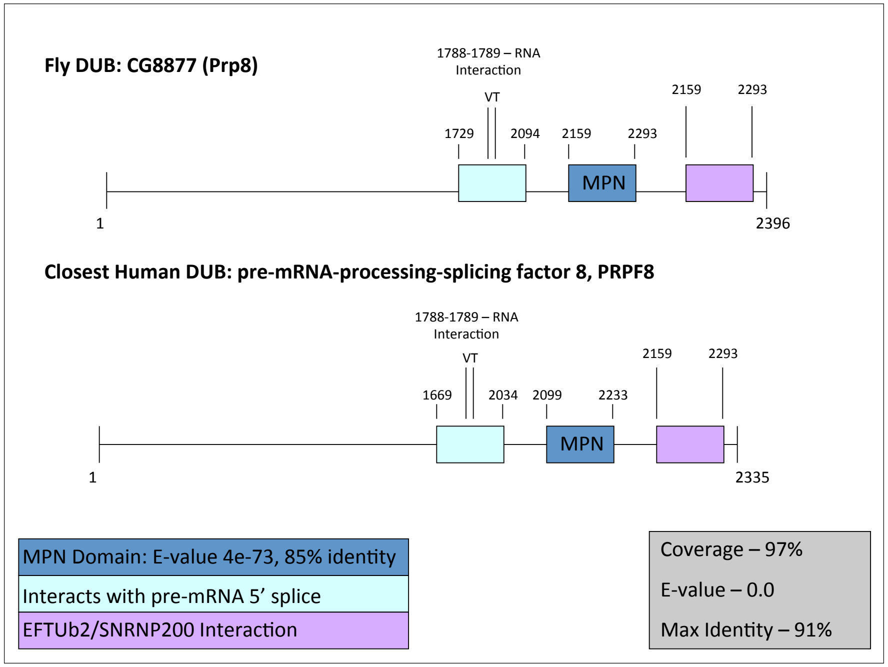
Fly DUB: CG8877 (Prp8)
1788-1789 – RNA Interaction
VT
1729
2094
2159
2293
MPN
2159
2293
1
2396
Closest Human DUB: pre-mRNA-processing-splicing factor 8, PRPF8
1788-1789 – RNA Interaction
VT
1669
2034
2099
2233
MPN
2159
2293
1
2335
MPN Domain: E-value 4e-73, 85% identity
Interacts with pre-mRNA 5' splice
EFTUb2/SNRNP200 Interaction
Coverage – 97%
E-value – 0.0
Max Identity – 91%
