# Supplementary material for: Systematic Analysis of the Physiological Importance of Deubiquitinating Enzymes
Source: PLoS One. 2012 Aug 24;7(8):e43112. doi: 10.1371/journal.pone.0043112 (PMC3427330; doi:10.1371/journal.pone.0043112)
Supplement: File S3 — Quantitative RT-PCR primers. (PDF) [file pone.0043112.s006.pdf]

SUPPLEMENTAL FILE 3

Fly Gene Identifier	Forward	Reverse
CG8445	CAACAACAGTCTCAGCCACAAT	GACGAATATCTCAGCAGTTGTCTC
CG4265	GTGCCGGTAATTGTGTGTAGAA	GAATCCATTCCAAGGTGTCATC
CG3431	ACGGTGCTGGAAATTGGTGTCTCATTG	AAGAAGATGTTCTCCCGATCCAG
CG5798	GCATTACAAGTCACCAACACCTTC	CCAGATTCTTCAGTCCAGTCAGT
CG8334	TTGATCTGTTCTACGGCCAGTT	AGTACTTGCAATCGGAGTTCAGAC
CG12082	GTACGAGATCAAGGACACGTACAG	GTCAGATTAAGCCAGAGGTTGTTG
CG1490	GATTACAATAGCACACGCTACGAG	GTACTTGATCGAGCTATCCGTGA
CG7023	AGCTAGCAATCATCACCAGACAAC	AGCTAGCAATCATCACCAGACAAC
CG5384	ACTCCTGTCAAATTCATTGAGGAC	CAAATATAGACTTCATGGCAGACG
CG8494	CTTGTGGAGTACATAGCAGAGCAG	CTGCTGCTGAAGTGACTGGTATT
CG3016	AATCGGTTACGTTCGCTAAACTAC	GGTAAAGATTCTTGGGGAACATC
CG5603	GAAGTCTCGAATCCTGGAGTATGT	GGCTGGTACAAAAATAGCTCGAC
CG3781	AAATCGGAACTGGACGACTACTG	CAGCTTGGAGTCCAGATTGTAGTA
CG9448	CGGTCCCTATTGTGATTGTGTTAT	GGTAGTTCTCGTAGGTGCAAGTTT
CG4603	ATGGCATACTGCTGAAGAAGGT	CTCAATGCCATAGTAGTTGGACAG
CG4968	GAGCCTTCGCCTACTCCTACTTG	AACCACCACGTAGTCTGAGTAACC
CG2224	AGGTGGTCTACCCCAATGACTTT	GTCTCGATGTTTTTCGAGGTGTT
CG18174	CTCAATCGCCACTACTACTCGAT	CATAACCTTGTCCACCTTCTCCT
CG6932	GAAGGAACAACAGTACAAGCAGGT	AGATCGATTAGGGACTCGAATAGC
CG9769	AAGCTGCTTCAGAAGACCGTAG	ACGGAGTGGATAAGGTCTAGCAG
CG3416	GATAGTGCATCCATTGGTTCTG	ACATATTCTCCAGGTAGTCGTGGT
CG5505	GTGTCCATTACGTTTCAGCACTTC	CTTGAAGGAAATCTGCTTGGTC
CG5486	ACGTATTCCCATCAAAATTCTGTC	TCGCTGATCTGTGATAATGAAAGT
CG5794	GGCCTTTAAGTACTTCACATCGAC	CTCTTCAGAGATCTGGTTCTCTACC
CG4165	GCCGTTCCTACTTAGCGTACTGA	ACTGAGGACACTTTACGCACAGC
CG7288	CAGTCGGACAAACAGTCAACAA	GGCATAAACATTGATCCTCGTC
CG7857	CAGTCCATCAGACACCAACTTATC	GATGAGATGGCCTTCAGTTCTATG
CG15817	ACCTTGCGCTGCTACAGTCTAC	GCTCTTGAGCCTCTTCAATTCT
CG14619	AACGGTCTATCTCTGCTCTCAACT	ATGTTTCAGGGATGACGAGTTC
CG8830	GGGGAATACCTGCTACATGAATAG	GATATCCTAGGAACTCGGAGCTGT
CG6091	CCTGTTTCGCTCTATATCCCTTC	CTGTAGATCTCGGAAATAGCTTGG
CG30421	CTTTAAGGCAGTGGTAGATCGGTA	GGTCGAATGTGTTTGACTGCTT
CG2904	GCTCTGACCTCCCAGAAAAGTCT	GTGTTTGCCGTAGTAGGAAACGAT
CG3430	CAGAAGACGGGAATCCTGAAGATA	CCTTGTCAGCATATTGTCCATCAG
CG2872	GGAATATGATGCAGAGAGTGTGG	CGTAACCACAATCATGTACTGGAC
CG33291	GGAATATGATGCAGAGAGTGTGG	CGTAACCACAATCATGTACTGGAC
CG7939 (rp49 control)	AGATCGTGAAGAAGCGCACCAAG	CACCAGGAACTTCTTGAATCCGG
CG5178 (actin control)	GTCTACAACTCGATCATGAAGTGC	TACTCCTGCTTCGAGATCCACAT
